# Synthesis and fluorescent properties of N(9)-alkylated 2-amino-6-triazolylpurines and 7-deazapurines

**DOI:** 10.3762/bjoc.15.41

**Published:** 2019-02-15

**Authors:** Andrejs Šišuļins, Jonas Bucevičius, Yu-Ting Tseng, Irina Novosjolova, Kaspars Traskovskis, Ērika Bizdēna, Huan-Tsung Chang, Sigitas Tumkevičius, Māris Turks

**Affiliations:** 1 Faculty of Materials Science and Applied Chemistry, Riga Technical University, P. Valdena Str. 3, LV-1048 Riga, Latvia; 2 Department of Organic Chemistry, Faculty of Chemistry and Geosciences, Vilnius University, Naugarduko Str. 24, 03225 Vilnius, Lithuania; 3 Department of Chemistry, National Taiwan University No.1, Section 4, Roosevelt Road, Taipei 106, Taiwan

**Keywords:** 7-deazapurines, fluorescence, nucleophilic aromatic substitution, purines, push–pull systems, pyrrolo[2,3-*d*]pyrimidines

## Abstract

The synthesis of novel fluorescent N(9)-alkylated 2-amino-6-triazolylpurine and 7-deazapurine derivatives is described. A new C(2)-regioselectivity in the nucleophilic aromatic substitution reactions of 9-alkylated-2,6-diazidopurines and 7-deazapurines with secondary amines has been disclosed. The obtained intermediates, 9-alkylated-2-amino-6-azido-(7-deaza)purines, were transformed into the title compounds by CuAAC reaction. The designed compounds belong to the push–pull systems and possess promising fluorescence properties with quantum yields in the range from 28% to 60% in acetonitrile solution. Due to electron-withdrawing properties of purine and 7-deazapurine heterocycles, which were additionally extended by triazole moieties, the compounds with electron-donating groups showed intramolecular charge transfer character (ICT/TICT) of the excited states which was proved by solvatochromic dynamics and supported by DFT calculations. In the 7-deazapurine series this led to increased fluorescence quantum yield (74%) in THF solution. The compounds exhibit low cytotoxicity and as such are useful for the cell labelling studies in the future.

## Introduction

**Figure 1:**
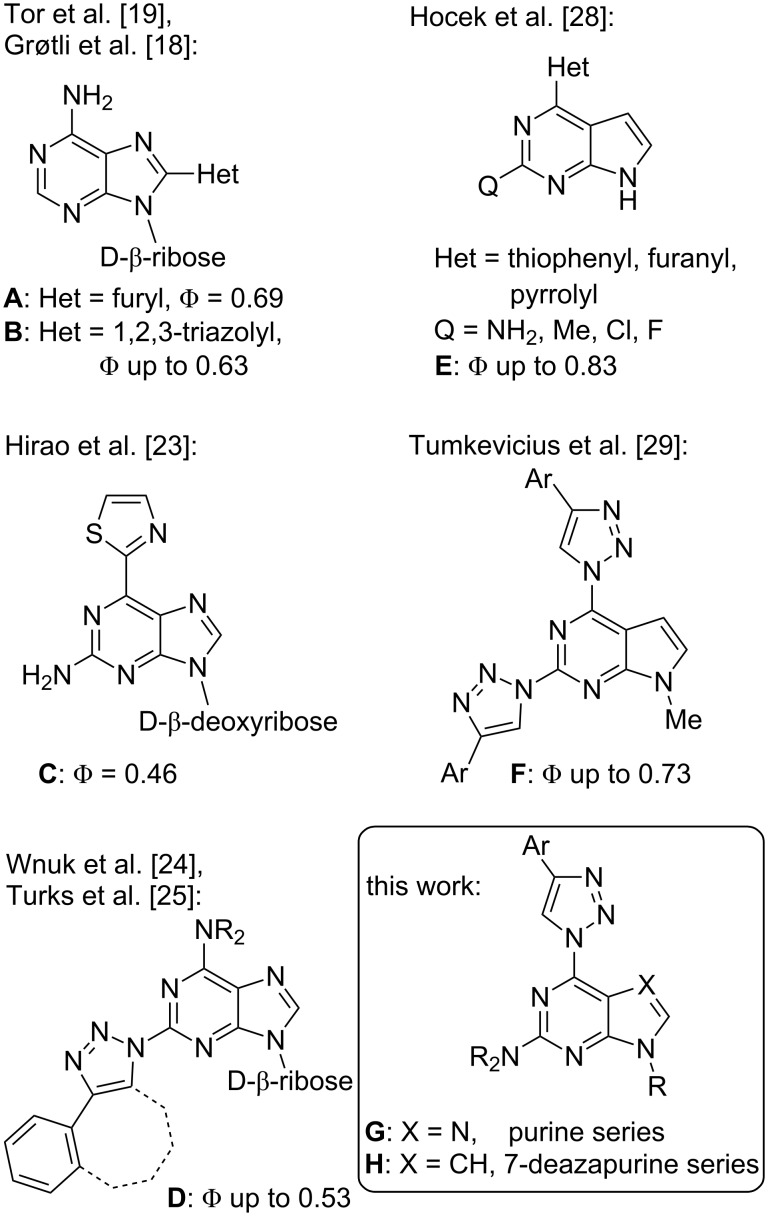
Examples of fluorescent purine/7-deazapurine derivatives.

Purine [1–7] and 7-deazapurine (IUPAC name: pyrrolo[2,3-*d*]pyrimidine) [8–11] derivatives have been progressively studied for decades due to their wide range of biological activities and photophysical properties. Currently, the synthesis of push–pull systems is a promising direction in the development of various fluorescent (7-deaza)purine derivatives [12–16]. The push–pull effect arises by adding electron-donating and electron-withdrawing groups at the opposite ends of π-conjugated systems. Large Stokes shifts and high quantum yields are usually characteristic for this type of molecules. Traditionally, fluorescent purine nucleoside analogs were recognized as valuable fluorescent probes for DNA and RNA research [17]. This paved a way for development of various adenosine and guanosine analogs which in many cases contained an additional substituent at C(8) like compounds **A** and **B** (Figure 1) [18–19]. The latter attachment does not significantly affect the natural Watson–Crick base-pairing abilities of the modified compounds. In this context, other important structural modifications in such a series include 8-vinyladenosine [20], 8-styryladenosine [13,21] and 8-heteroarylguanosine [22] derivatives which revealed good to excellent quantum yields. Useful levels of fluorescence were reported also for purine nucleos(t)ides bearing azole-type substituents at C(2) or C(6) (e.g., 6-thiazol-2-yl derivative **C** [23] and 2-(1,2,3-triazol)-1-yladenosines **D** [24–25] (Figure 1).

7-Deazapurines can be regarded as structurally close congeners to purines with an extra attachment point at their “artificially introduced” C(7). Therefore, it comes as no surprise that also 7-deazapurine derivatives containing heterocyclic substituents at various attachment points [26–27] have been actively studied for their fluorescent properties. Among others, also azole containing compounds of type **E** [28] and **F** [29] have been described.

Similarly, to purine derivatives also 7-deazapurines are used in DNA labeling [30]. On the other hand, modified purines have found also applications as cell imaging tools [31–32], biosensors and sensors for detection of heavy metals [33]. The tendency of these structures to form hydrogen bonds, to respond to medium effects (solvatochromism) [34] and to certain impurities (e.g., coordination complexes with metal ions) make these compound classes very attractive for sensor design [33,35]. It is interesting to note, that push–pull type purine non-nucleos(t)ide derivatives have not been actively studied in the context of materials science. The few available examples include the design of methyl 9-benzyl-2-*N*,*N*-dimethylamino-9*H*-purine-8-carboxylate and the corresponding 2,6-bis(dialkylamino) derivatives as fluorescent materials for preparation of organic light-emitting diodes [12,36–38].

In light of the aforementioned facts, we proceeded to design novel and structurally related classes of 2-amino-6-(1,2,3-triazol-1-yl)purines and 7-deazapurines (Figure 1 compounds of type **G** and **H**) in order to determine and to compare their fluorescent properties. The synthetic approach towards 7-deazapurine derivatives **H** has been recently disclosed by us [39], but the photophysical properties of these compounds have not been studied thus far. In this full account we report the newly developed C(2)-regioselective nucleophilic aromatic substitution of 2,6-diazidopurines, transformation of the intermediate 2-amino-6-azidopurines into the title compounds and comparative photophysical study of originally yet identically substituted fluorescent push–pull purines and 7-deazapurines.

## Results and Discussion

We have recently observed significant fluorescence in the triazolylpurine and triazolyl-7-deazapurine series [25,29,40–41]. Therefore, we developed synthetic methods that can provide structurally related chromophores of the purine and 7-deazapurine series possessing identical substitution pattern (structural congeners **G** and **H**, Figure 1). The easy access to the title compounds opened a possibility for a comparative study of their photophysical properties.

### Synthesis

N(9)-Alkylated-2,6-diazidopurine **2a** was synthesized in two steps from commercially available 2,6-dichloropurine (**1a**) by subsequent alkylation of the N(9) position and azido group introduction in C(2) and C(6) positions. The N(9) position of purine was alkylated in two different ways: 1) using alkyl halides in the presence of a strong base, and 2) using alcohols under Mitsunobu conditions.

**Scheme 1:**
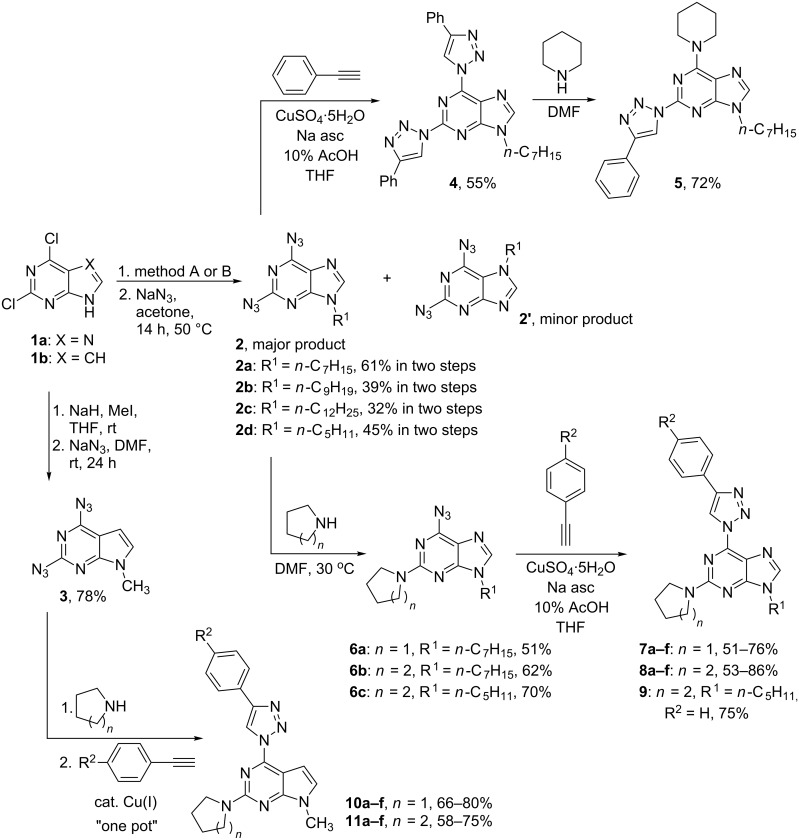
General synthetic routes for the compounds **5**, **7**–**9**, **10** and **11**. Method A: alkyl halogenide, MeCN or DMF, NaH, 1–3 days, 20–55 °C; method B: alcohol, Ph_3_P, THF, DIAD, 1 h, 0→20 °C.

In the first approach (method A, Scheme 1), the purine was deprotonated with a base (NaH or K_2_CO_3_) and alkylated with alkyl halides such as 1-iodoheptane, 1-bromononane and 1-bromododecane. The main disadvantages of this approach are suboptimal yields, which varied from 48% to 52%, and long reaction times. For example, in the case of 1-bromododecane, the reaction required 72 hours to complete. On the other hand, Mitsunobu reaction (method B, Scheme 1) was performed between the corresponding alcohol and 2,6-dichloropurine in anhydrous THF at 0–20 °C for 1–1.5 h, resulting in average yields of 66% for N(9)-alkylated products. Both methodologies produced a mixture of N(9)- and N(7)-alkylated isomers from which the major N(9) product was easily separated by column chromatography [42–47]. Alkylated products were used in S_N_Ar reactions with NaN_3_ in acetone or DMF, giving good to excellent yields of 2,6-diazidopurines **2a–c** in the range from 66 to 93%. In several cases chromatographic separation of the N(9)/(7) isomers was easier at the stage of diazido products **2a** and **2a’**. Diazido products **2** are light and temperature sensitive [48], but can be stored without degradation in refrigerator at −20 °C for a prolonged period of time. In addition, DSC measurements for compounds **2a–c** were done. Diazides **2a–c** melted at 67 °C, 75 °C and 82 °C, respectively, and showed an exothermic effect only if heated above 190 °C (Figures S5–S7 in Supporting Information File 1).

In the 7-deaza series deprotonated starting material **1b** was methylated with MeI and further submitted to S_N_Ar reaction with NaN_3_ according to a previously published procedure [39]. The expected 2,6-diazido-7-deazapurine (**3**) was obtained in 78% yield.

It should be noted that the medium size alkyl chains at N(9) were chosen for several reasons: a) they help to solubilize the target compounds in the organic medium; b) they are considered as a good compromise to achieve both “membrane-like character”, which helps for cell membrane permeability, and sufficient solubility in the aqueous (biological) media [49].

With the key intermediates **2** and **3** in hand, we proceeded with the synthesis and structure elucidation of the designed structures **G** and **H** (Figure 1) which are represented by compounds **7**–**11** in Scheme 1. Firstly, we prepared a regioisomeric compound **5** by repeating the previously elaborated sequence of double CuAAC reaction (**2**→**4**) followed by a S_N_Ar process (**4**→**5**) which showcases the use of 1,2,3-triazoles as leaving groups. It is well established during our previous research in the purine nucleoside series that such an approach provides 6-amino-2-(1,2,3-triazol-1-yl) derivatives [25]. On the other hand, it was also reported that 2,6-diazidopurine nucleosides exhibit opposite regioselectivity with aliphatic thiols, which do S_N_Ar reactions at C(2) position of purines [50–51]. Taking this information into account, we performed an S_N_Ar reaction between 2,6-diazidopurine **2a** and piperidine and obtained the C(2)-substitution product **6b** as the major isomer. To the best of our knowledge, this is the first report on such regioselectivity involving combination of 2,6-diazidopurines and N-nucleophiles.

**Figure 2:**
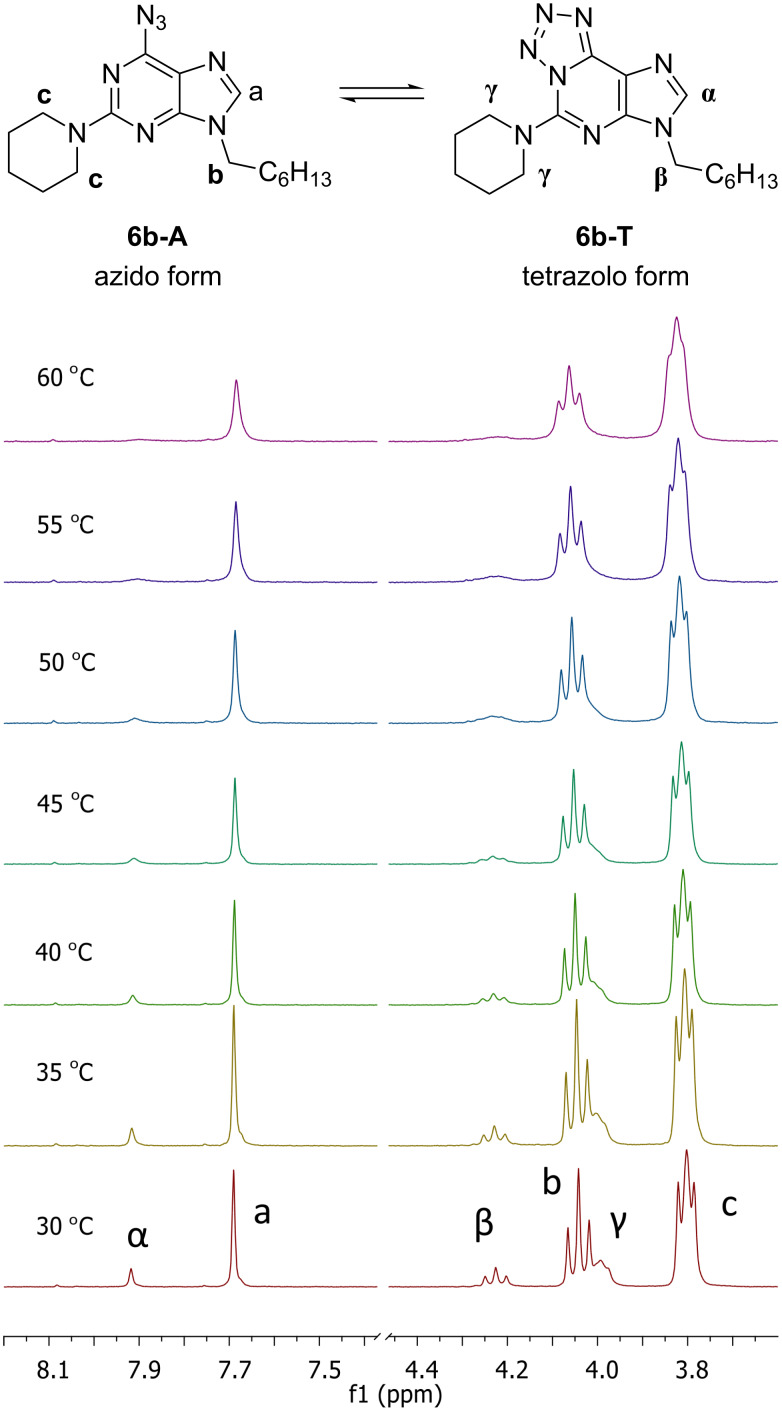
^1^H NMR spectra of compound **6b** in CD_3_CN at different temperatures (300 MHz, *c* = 12.5 mg/mL); a, b, c – signals for azide form **6b-A**; α, β, γ – signals for tetrazole form **6b-T**).

It should be noted that in solution, azides **6** exist in equilibrium between the azidopurine and tetrazolopurine forms. This equilibrium was studied in different deuterated solvents by ^1^H NMR spectroscopy on the example of 6-azido-9-heptyl-2-piperidinopurine **6b**. Experiments were performed in CDCl_3_, THF-*d*_8_, CD_3_CN and DMSO-*d*_6_. The ^1^H NMR spectrum in CD_3_CN showed the presence of the tetrazole form and has been used for NMR studies in the temperature range from 30 to 60 °C using two different concentrations – 12.5 mg/mL and 25.0 mg/mL. Temperature increase caused the increase of the azido form. These results are summarized in Figure 2. The temperature increase caused the disappearance of signals at 7.92 (s, α), 4.23 (t, β) and 4.01–3.95 (m, γ) and the broadening of signals at 7.69 (s, a), 4.04 (t, b) and 3.84–3.74 (m, c). A similar type of the ring–chain tautomerism is expected to occur in 6-azido-2-amino-7-deazapurines (synthetic intermediates on the way **3**→**10/11**), but it was not studied in detail due to their relatively low stability [39]. Regarding the tautomerism in 2,6-diazido-substituted starting materials, it has been studied previously for both purine [41] and 7-deazapurine [52] derivatives. In both cases practically only the diazido forms are observed in chloroform solution, but the proportion of the tetrazole tautomer increases with the increase of solvent polarity. Additionally, X-ray analysis of 2,6-diazidopurine 2'-deoxyribonucleoside revealed the exclusive existence of the azido tautomer in the solid state [41].

The discovered ability of the azido group to be substituted with amines at C(2) position was used for the synthesis of a novel library of 9-alkyl-2-amino-6-triazolylpurine derivatives. Thus, copper-catalyzed azide–alkyne 1,3-dipolar cycloaddition reaction of compounds **6a–c** with different *para*-substituted phenylacetylenes produced the expected compounds **7a–f**, **8a–f** and **9** (Scheme 1). The yields of 1,3-dipolar cycloadditions varied from 51% to 76% in the case of pyrrolidinyl purines **7a–f** and from 53% to 86% for piperidinyl purines **8a–f** (Table 1 and Table 2). To gain an insight into the structure–photophysical properties relationship of the newly obtained structures, various EDG and EWG at the *para*-position of the phenyl rings were installed. It can be concluded that 2,6-diazidopurines are versatile intermediates for the synthesis of both the 2-amino-6-triazolylpurine and 6-amino-2-triazolylpurine derivatives. Comparison of both synthetic methods (**2**→**4**→**5** versus **2**→**6**→**7/8/9**) reveals that the newly developed approach towards 2-amino-6-triazolylpurine derivatives was performed in a more atom economic way. The latter synthesis does not require a double cycloaddition followed by replacement of one of the triazole moieties.

**Table 1:** Diversity of 9-alkyl-2-pyrrolidino-6-triazolylpurine **7** and 7-deazapurine **10** derivatives obtained in reactions **6a**→**7a–f** and **3**→**10a–f** according to Scheme 1.

Entry	General structure	Compound	R	Yield, %

1	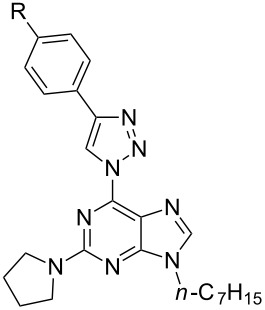 **7**	**7a**	-H	51
2		**7b**	-OMe	57
3		**7c**	-NMe_2_	53
4		**7d**	-F	64
5		**7e**	CF_3_	76
6		**7f**	-CN	63

7	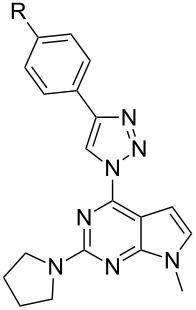 **10**	**10a**	-H	71
8		**10b**	-OMe	66
9		**10c**	-NMe_2_	68
10		**10d**	-F	80
11		**10e**	-CF_3_	76
12		**10f**	-CN	79

**Table 2:** Diversity of 9-alkyl-2-piperidino-6-triazolylpurine **8** and 7-deazapurine **11** derivatives obtained in reactions **6b**→**8a–f** and **3**→**11a–f** according to Scheme 1.

Entry	General structure	Compound	R	Yield, %

1	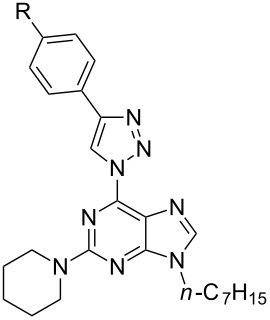 **8**	**8a**	-H	71
2		**8b**	-OMe	60
3		**8c**	-NMe_2_	53
4		**8d**	-F	74
5		**8e**	-CF_3_	84
6		**8f**	-CN	86

7	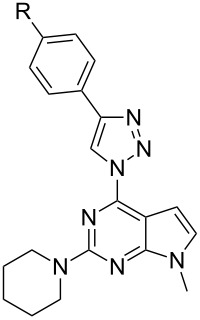 **11**	**11a**	-H	65
8		**11b**	-OMe	60
9		**11c**	-NMe_2_	58
10		**11d**	-F	68
11		**11e**	-CF_3_	63
12		**11f**	-CN	75

**Figure 3:**
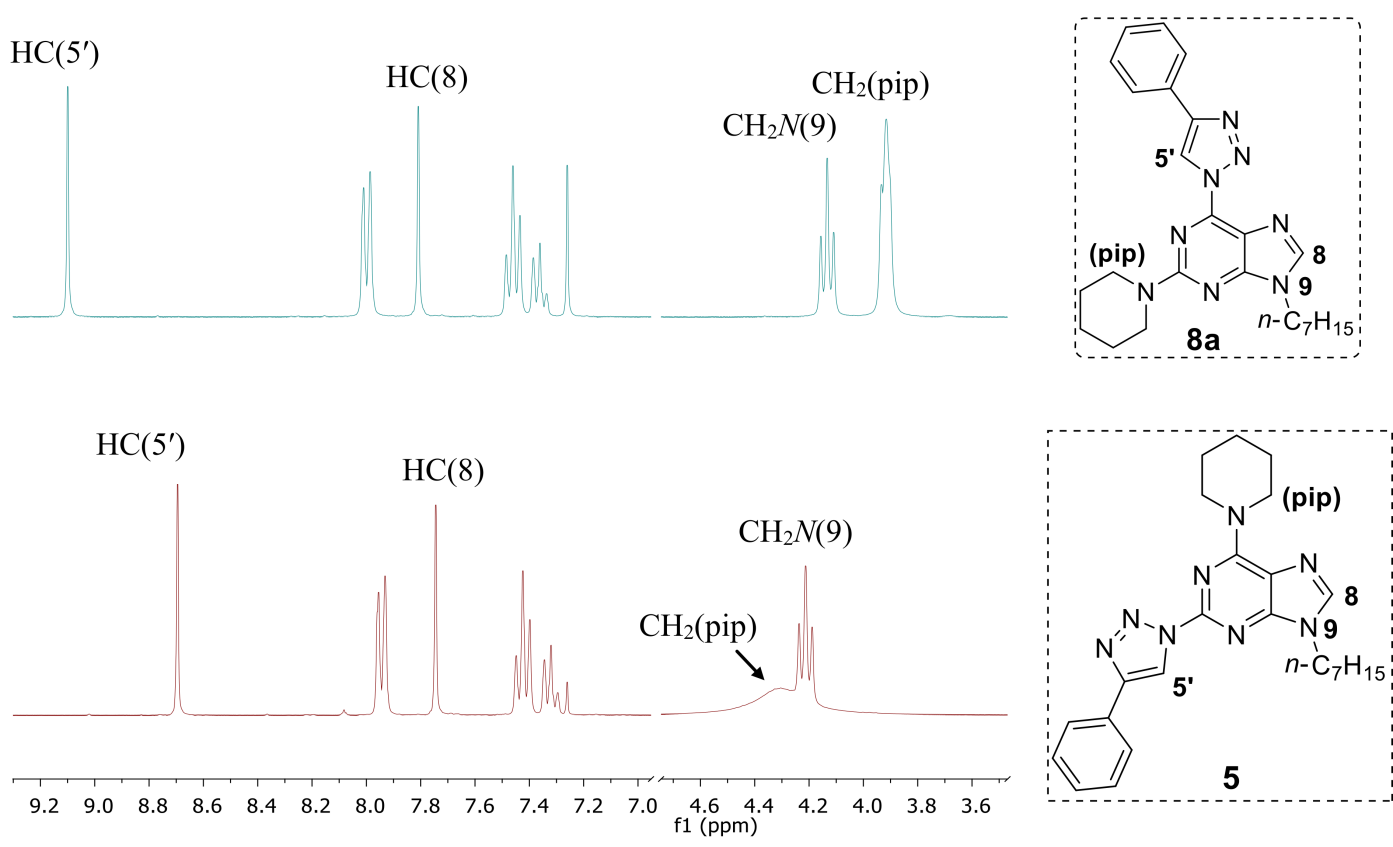
Comparison of ^1^H NMR spectra of compounds **8a** and **5** (300 MHz, CDCl_3_).

**Figure 4:**
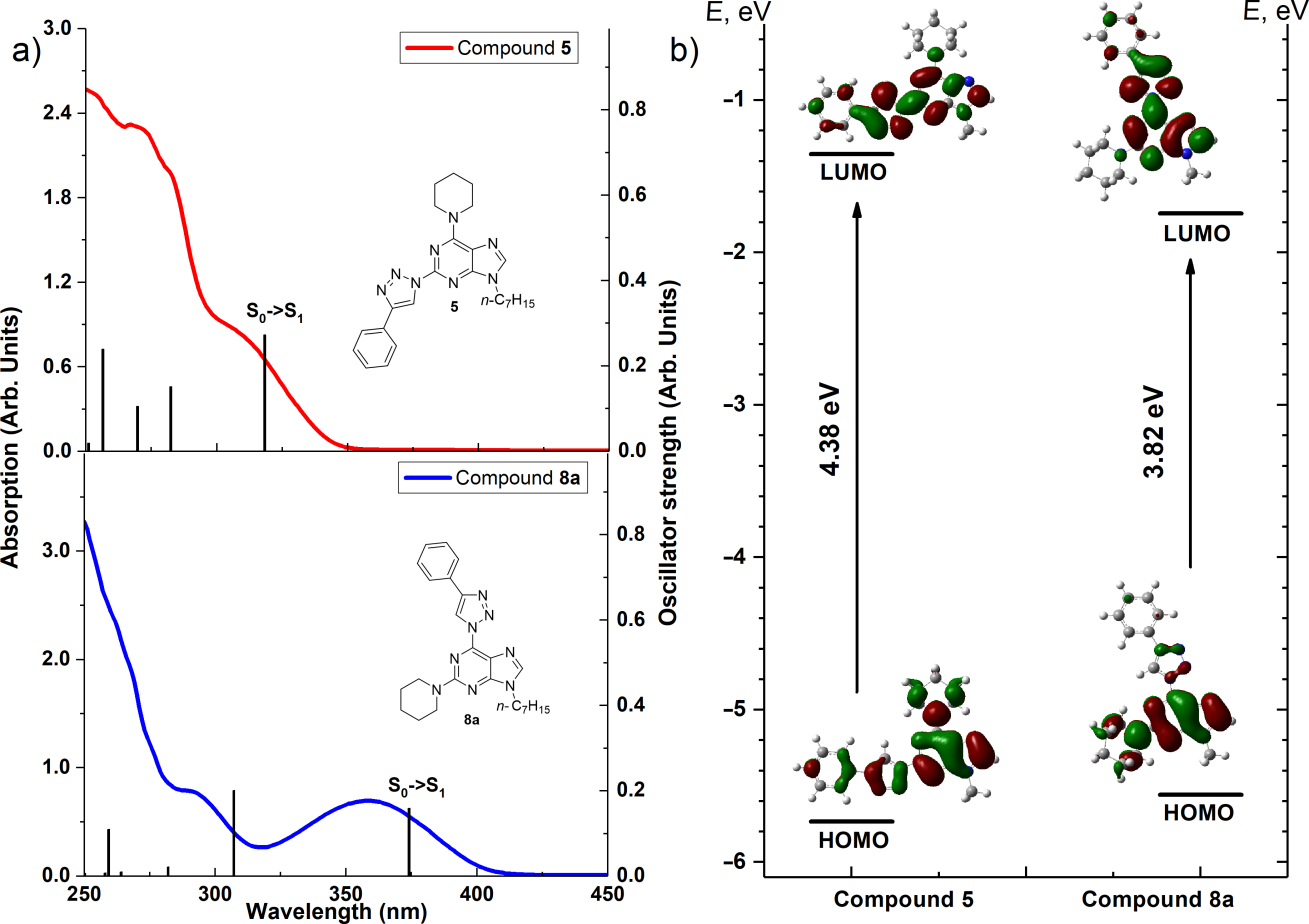
a) Experimental UV–vis absorption spectra (lines) with computed theoretical absorption bands (columns) of compounds **5** and **8a** (*c* = 10^−4^ M). b) Energy diagram and spatial distribution of frontier molecular orbitals (at isosurface value 0.02) of compounds **5** and **8a**.

The final structural proof for the compounds of the series **7a–f** and **8a–f** was obtained by spectral comparison between compound **8a** and regioisomeric compound **5**. The ^1^H NMR comparison showed two main differences in the spectra (Figure 3): 1) the H–C(triazole) signal of product **8a** is shifted downfield from 8.69 to 9.10 ppm and 2) the appearance of a CH_2_ signal near the *N* atom is changed from multiplet at 3.96–3.87 ppm to broad singlet at 4.60–3.95 ppm. Furthermore, a significant difference is observed also in the UV spectra. Product **8a** has an additional absorption maximum at 370 nm (Figure 4).

In the 7-deazapurine series the S_N_Ar reactions between 2,6-diazido-9-methyl-7-deazapurine (**3**) and pyrrolidine or piperidine were more regioselective than in the purine series. In the latter case the purine products **6a–c** had to be chromatographically separated to remove the 2-azido-6-amino isomer as the purification at the stage of final products was not effective. The observed C(2)-selectivity in the 7-deazapurine series allowed to combine the S_N_Ar and CuAAC reactions into an sequential one-pot process producing target products **10** and **11** directly from diazide **3**. The 7-deazapurine structural analogs **10a–f** and **11a–f** to every purine entry were obtained with 58–80% isolated yields.

### UV–vis and fluorescence data

The optical properties of the synthesized compounds were assessed by performing absorption and fluorescence spectroscopy and fluorescence quantum yield measurements. The quantum yields were determined using a fluorescence standard of quinine sulfate in 0.1 M H_2_SO_4_ as a reference [53]. Absorption and fluorescence data of the investigated compounds in MeCN solutions are summarized in Table 3. The newly developed purine and 7-deazapurine derivatives exhibit similar photophysical properties to previously reported push–pull systems containing the same central molecular scaffold (see Table 3 versus Figure 1). There are only few other modified purines and 7-deazapurines known for which higher quantum yields were reported. The latter include Castellano’s purines (QY up to 95%) containing electron-withdrawing substituents at C(8) [36–37] and 2-halo-7-deazapurine derivatives (QY up to 83%) reported by Hocek et al. [28].

**Figure 5:**
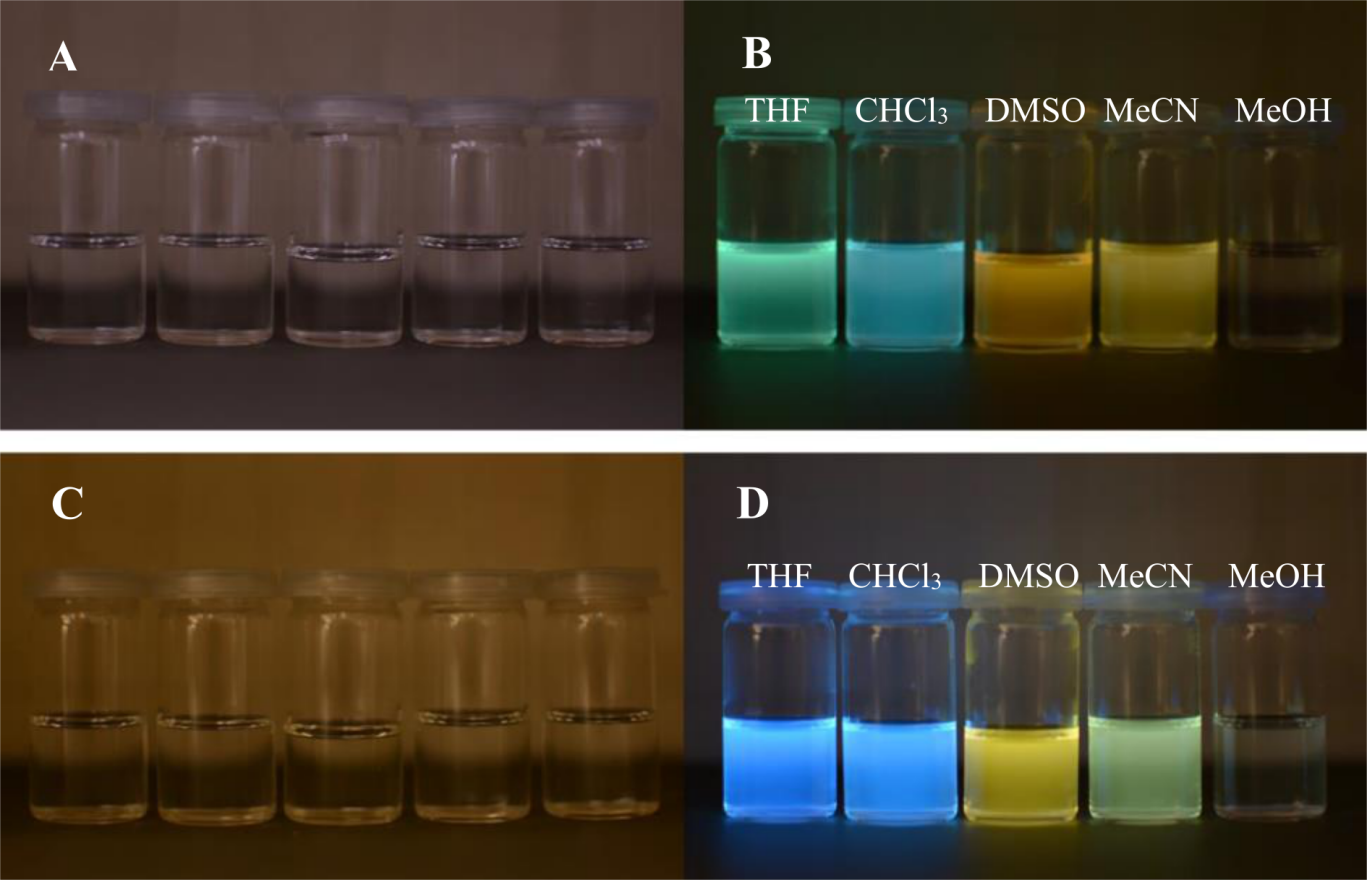
Photos of compound **8c** (A and B) and compound **11c** (C and D) in THF, CHCl_3_, DMSO, MeCN and MeOH before excitation (A and C) and under excitation (B and D) of UV (366 nm).

**Figure 6:**
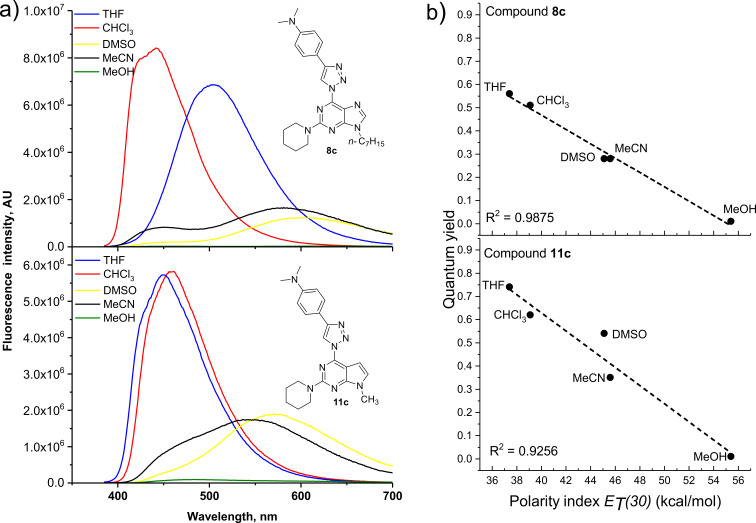
a) Fluorescence spectra of compounds **8c** (λ_exc_ = 360 nm) and **11c** (λ_exc_ = 370 nm) in solvents of different polarity, *c* = 10^−4^ M. b) Quantum yield dependence on the Dimroth–Reichardt polarity parameter *E*_T_(30) with a linear fit for compounds **8c** and **11c**.

**Figure 7:**
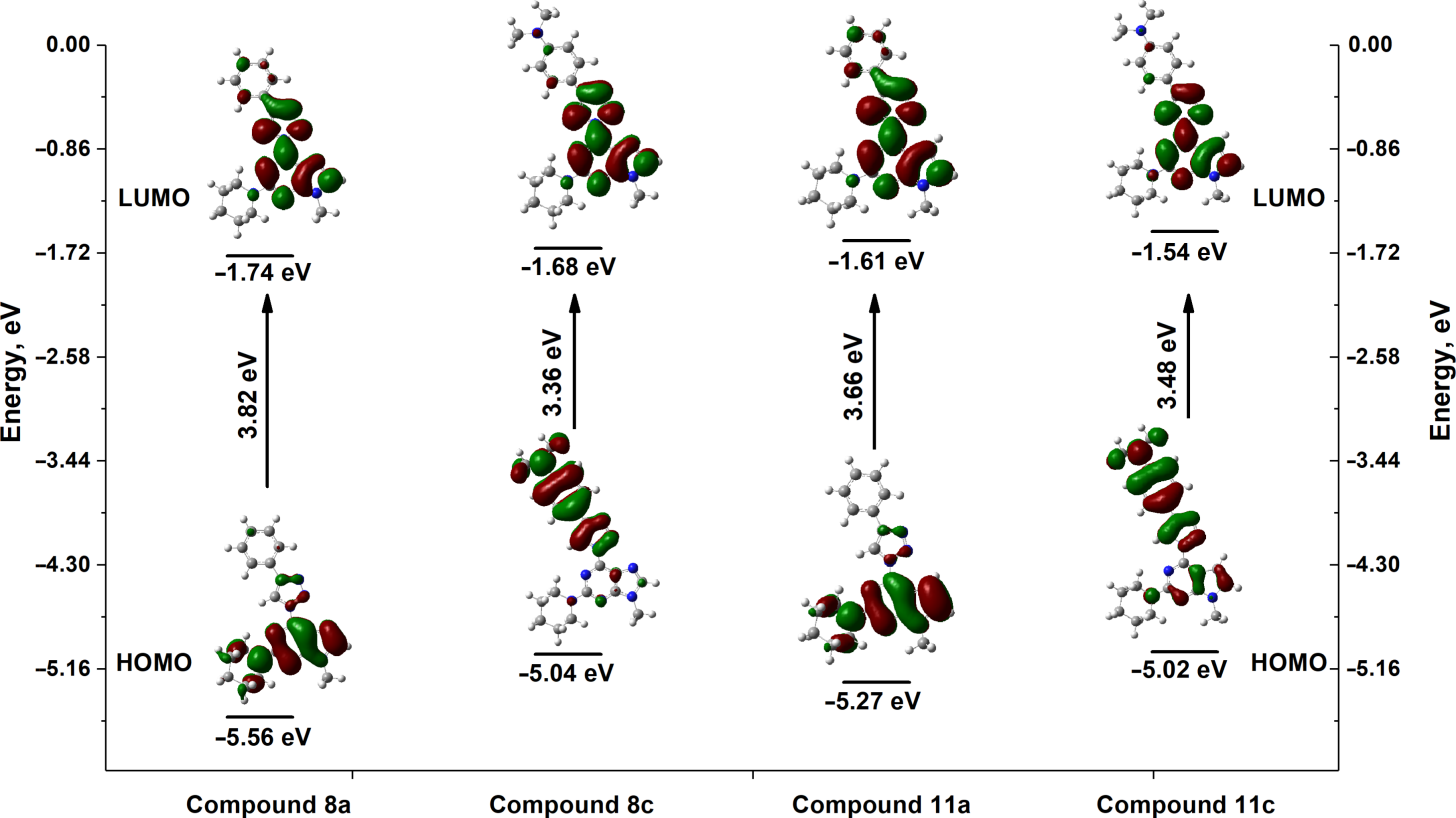
Energy diagram for the frontier molecular orbitals of compounds **8a**, **8c**, **11a** and **11c**.

In our case the lowest-energy absorption band was observed approximately at 360 nm for the purine class compounds (Table 3, entries 1–6 and 13–18), whereas for the 7-deazapurine series (Table 3, entries 7–12 and 19–24) the lowest-energy absorption band was slightly red-shifted by 15 nm to ≈375 nm. A similar trend was observed in the fluorescence spectra of the studied compounds – emission maxima for the purine class compounds was observed at 442–452 nm, whilst it was red-shifted for 7-deazapurine series to 468–472 nm, moreover most of the studied compounds possessed very similar quantum yields 0.56–0.61 for purine and 0.50–0.56 for 7-deazapurine series, with only a few exceptions from a trend (Table 3, entries 3, 9, 12, 15, 21 and 24). Compounds **10f** and **11f** from the 7-deazapurine class (Table 3, entries 12 and 24), which possess -CN substituents on the *para* position of the phenyl ring, showed slightly lower quantum yields of 0.36–0.38 and a red shift of the emission maxima by 10–15 nm, whilst similar purine class compounds (Table 3, entries 6 and 18) remained on a previously discussed trend, thus probably indicating a minor change in electronic structure for compounds **10f** and **11f**. Also, lower quantum yields of 0.28–0.35 and highly red-shifted emission maxima to 528–581 nm were recorded for the compounds possessing strong electron-donating -NMe_2_ groups (Table 3, entries 3, 9, 15, 21). In addition, it was found that these compounds possess a strong positive solvatochromic effect, which was studied in detail in solvents of different polarity (THF, CHCl_3_, DMSO, MeCN and MeOH). The study was performed on two model compounds, **8c** from the purine class and **11c** from the 7-deazapurine class (Figure 5 and Figure 6). As illustrated in Figure 5 and Figure 6 the emission of compounds **8c** and **11c** showed pronounced changes with solvent polarity in terms of emission maxima and quantum yields, though the absorption spectra showed minor or no changes with increasing solvent polarity indicating small dipole moment in the ground state. A good linear correlation between the fluorescence QY and the Dimroth–Reichardt polarity parameter (*E*_T_(30)) [54] was observed (Figure 6) for both classes of compounds. For example, the fluorescence QY of 7-deazapurine **11c** in THF were determined to be 0.76 and non-detectable in methanol. Similarly, QY of purine **8c** dropped from 0.56 to <0.01 by switching between the same solvents (Table 4). Non-linear dependency in increase of emission maxima is observed (Table 4) with increasing polarity parameter (*E*_T_(30)), thus indicating that the sensitivity to solvent polarity is not the only major solute–solvent interaction and the emission maxima are also influenced by other solvent properties such as viscosity or hydrogen bonding. Interestingly, purine class compound **8c** showed a dual-fluorescence character in the solvents of higher polarity (DMSO, MeCN) arising from the locally excited and charge transfer excited states, however, dual-fluorescence was not evident for the 7-deazapurine class, which could be explained by a better stabilization of the ICT excited state by the solvatic shell. A decrease of QY accompanied by a high red shift of the emission maxima with increasing polarity of the surrounding media is a typical characteristic for the enhanced ICT of the excited states with a possible character of TICT [55], which could explain a dual-fluorescence and fluorescence quenching in the high polarity media. The charge transfer nature of the transitions for the compounds **8c** and **11c**, compared to reference compounds **8a** and **11a**, was confirmed by quantum chemical calculations. Computed spatial distributions of FMO’s and comparison of electronic structures revealed that where is almost no change in energy and distribution of LUMO, which is localized over the extended triazolyl(deaza)purine core (Figure 7). However, a major change in HOMO localization and energy is observed: 1) the HOMO is mostly localized on the central (deaza)purine core and piperidino substituent for the reference compounds **8a** and **11a**; 2) the HOMO is mostly distributed over the electron-donating Me_2_N groups, phenyl ring and the 1,2,3-triazolyl moiety for compounds **8c** and **11c**. Thus, these findings support an enhanced ICT character of the excited states for the compounds possessing strong electron-donating Me_2_N groups.

**Table 3:** Photophysical properties of 6-triazolyl compounds **7a–f**, **10a–f**, **8a–f** and **11a–f**.^a^

Entry	Structure	Compound	R	λ_abs_, nm	λ_em_^b^, nm	Δλ, nm	Φ

1	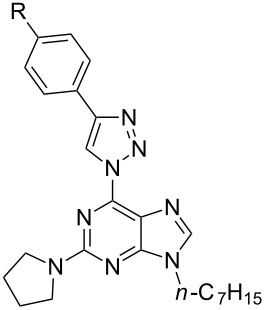 **7**	**7a**	-H	360	444	84	0.59
2		**7b**	-OMe	359	442	83	0.61
3		**7c**	-NMe_2_	361	577	216	0.32
4		**7d**	-F	360	444	84	0.57
5		**7e**	-CF_3_	362	452	90	0.58
6		**7f**	-CN	363	453	90	0.58

7	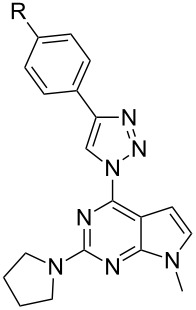 **10**	**10a**	-H	375	472	97	0.52
8		**10b**	-OMe	374	466	92	0.56
9		**10c**	-NMe_2_	369	528	159	0.35
10		**10d**	-F	375	472	97	0.53
11		**10e**	-CF_3_	377	468	91	0.54
12		**10f**	-CN	378	482	104	0.36

13	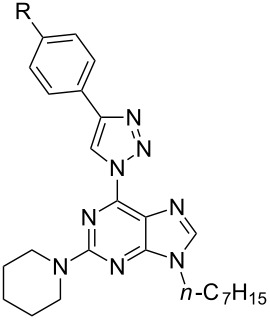 **8**	**8a**	-H	357	445	88	0.57
14		**8b**	-OMe	357	444	87	0.59
15		**8c**	-NMe_2_	360	445; 581	85; 221	0.28^c^
16		**8d**	-F	360	449	89	0.56
17		**8e**	-CF_3_	357	453	96	0.57
18		**8f**	-CN	360	456	96	0.60

19	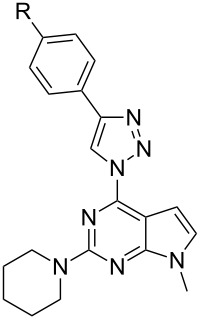 **11**	**11a**	-H	372	472	100	0.54
20		**11b**	-OMe	371	471	100	0.52
21		**11c**	-NMe_2_	368	536	168	0.35^d^
22		**11d**	-F	373	473	100	0.50
23		**11e**	-CF_3_	375	481	106	0.51
24		**11f**	-CN	376	485	109	0.38

^a^All spectra were recorded in MeCN solutions (*c* = 10^−4^ M) at room temperature; ^b^compounds **7** and **8** were excited at 360 nm and compounds **10** and **11** were excited at 370 nm; ^c^fluorescence lifetime: 7.79 ± 0.03 ns; ^d^fluorescence lifetime: 8.77 ± 0.03 ns.

**Table 4:** Emission maxima and quantum yields of compound **8c** and **11c** in solvents of different polarity.

Entry	Compound	Solvent	λ_em_, nm^a^	Δλ	Φ

**1**	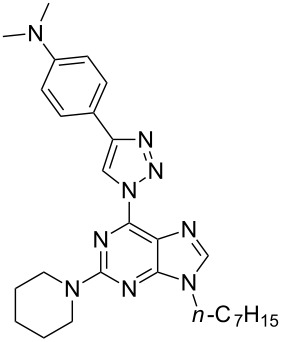 **8c**	THF	504	147	0.56
2		CHCl_3_	442	76	0.51
3		DMSO	443; 604	80; 241	0.28
4		MeCN	445; 581	85; 221	0.28
5		MeOH	460; 619	96; 255	<0.01

6	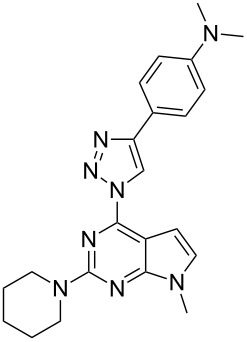 **11c**	THF	449	76	0.74
7		CHCl_3_	461	88	0.62
8		DMSO	572	196	0.54
9		MeCN	536	166	0.35
10		MeOH	-^b^	-^b^	0^b^

^a^Compound **8c** was excited at 360 nm and compound **11c** at 370 nm. ^b^Fluorescence was lower than the detection limit.

In summary, moderate to high quantum yields were observed for the studied purine and 7-deazapurine compounds. According to the expectations, the choice of alkyl chains and tertiary amine (piperidine or pyrrolidine) substituents did not significantly influence fluorescent properties. The preliminary study showed that introduction of other aliphatic amines at C(2) does not significantly affect the photophysical properties of the target substances either. On the other hand, the present synthetic development does not allow yet to introduce arylamino moieties at C(2) due to the diminished nucleophilicity of anilines.

The 7-deazapurine derivatives were characterized with a somewhat larger Stokes shift and bathochromic shift of the lowest energy absorption band in comparison to purine derivatives, but resulted in slightly lower quantum yields. Except for the compounds with strong electron-donating Me_2_N substituents and ICT/TICT character, where QY were slightly higher for 7-deazapurine derivatives. As the emission properties of compounds (**7c**, **8c**, **10c** and **11c**) possessing the ICT/TICT character of the excited states are environment-dependent it makes these fluorophores interesting as potential sensors of the surroundings such as polarity or (micro)viscosity sensors. Other purine and 7-deazapurine derivatives, which efficiently emit light in the blue region with respect to the structural biocompatibility could be applied for the cell staining in fluorescence microscopy.

### Applications in the cell studies

**Figure 8:**
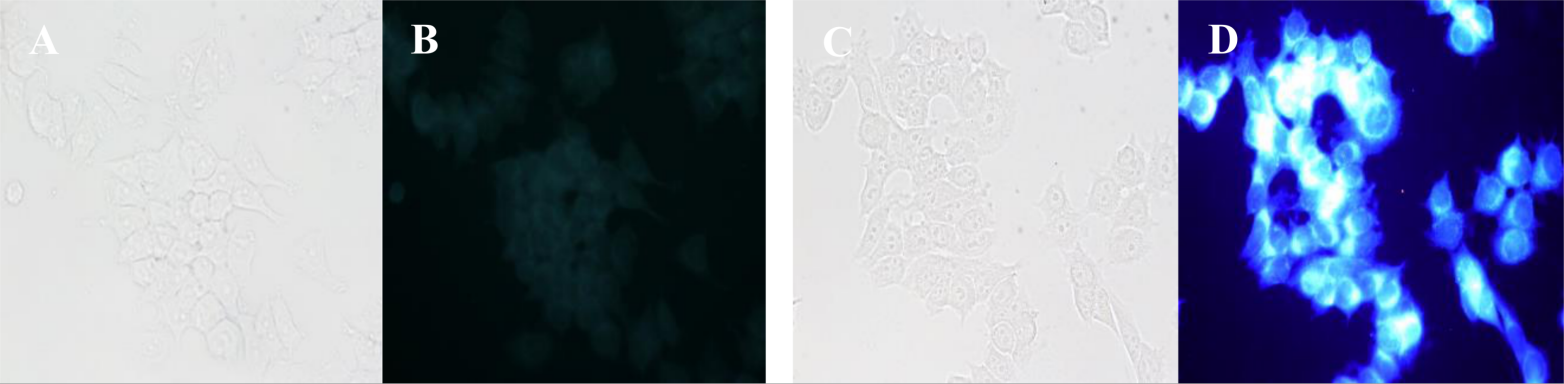
Labeled MCF-7 cells using compound **9** (C,D) and unlabeled MCF-7 cells (A,B) in microscope (2 h, *c*(**9**) = 100 μM, B and D passed separately through filter (365 ± 20 nm)).

The newly prepared compound library with purine and 7-deazapurine derivatives was submitted to the screening of their biological activity. The compounds of interest were studied on two cancer cell lines – luminal A breast cancer cell line MCF7 and triple negative breast cancer cell line MDAMB231. The results were compared with those obtained on normal breast epithelial cell line (MCF-10A). All compounds showed low cytotoxicity on all tested cell lines (for Figure S81 see Supporting Information File 1). Bearing in mind fluorescent properties and low cytotoxicity of the newly obtained compounds, we tested their potential application in cell staining. The preliminary experiments revealed that the compounds went through the cell membrane after 1 h or 2 h incubation period and localized uniformly in the cytoplasm. The representative fluorescent image of the labeled MCF-7 cells with compound **9** is shown in Figure 8 (for other examples see Figure S82 in Supporting Information File 1).

Based on the recent investigation of metabolic labelling of DNA [30,56] by deaza-7-ethenyl-2'-deoxyguanosine and 7-deaza-7-ethenyl-2'-deoxyadenosine or 5-(azidomethyl)-2'-deoxyuridine, we can assume that nucleoside analogs bearing fluorophores of our type would be susceptible to metabolic phosphorylation and incorporation into DNA in the living cells.

Further studies are required to develop the newly described *N*(9)-alkylated-2-amino-6-triazolylpurines and 7-deazapurines as cell labeling agents for biological chemistry applications.

## Conclusion

A group of novel and structurally related N(9)-alkylated 2-amino-6-triazolylpurines and 7-deazapurines was obtained, using the corresponding 2,6-diazido derivatives as key starting materials. We have reported here for the first time that 9-alkyl-2,6-diazidopurines exhibit C(2)-selectivity in nucleophilic aromatic substitution reactions with amines. A similar selectivity was observed also for 9-alkyl-2,6-diazido-7-deazapurines. We have also demonstrated that the synthetic intermediates obtained in the S_N_Ar reaction (e.g., 6-azido-9-heptyl-2-(piperidin-1-yl)purine (**6b**)) exists in both tetrazolo- and azido-tautomeric forms in CD_3_CN solution. The presence of the latter permits the CuAAC reaction with terminal acetylenes and gave a rise to the title compounds.

The obtained compounds possess useful levels of fluorescence in acetonitrile solution with quantum yields ranging from 28% to 60% with emission maxima positioned at 442–581 nm. It should be noted that still the biggest challenge in manufacturing of affordable OLEDs is emission of the color blue. Most of here reported compounds emit blue light and thus are important novel structural entities to be studied in the context of OLEDs in the future. Compounds containing a 4-(4-dimethylaminophenyl)-1,2,3-triazol-1-yl substituent have shown a strong solvatochromic effect including the increase of fluorescence quantum yield to 74% in the case of 7-deazapurine derivative **11c**. The solvent change provided a fluorescence shift from dark blue (≈440 nm) to orange (≈620 nm) color.

Finally, the purines and 7-deazapurines were tested in live cell imaging on breast cancer cell lines MCF-7 and MDAMB231. They are biocompatible, cell-permeable, not cytotoxic and do not influence cell proliferation. Thus, one can predict that the developed purine and 7-deazapurine derivatives possessing the novel substitution pattern may find their application also in cell labeling in the future besides the potential use as materials in organic light emitting diodes. The latter study which uses structurally related fluorescent purine derivatives containing sterically bulky amorphousing groups at position N(9) for formation of fluorescent molecular glasses are underway in our laboratories and will be reported in due course.

## Experimental

### General information

Reactions and purity of the synthesized compounds were monitored by TLC using Silica gel 60 F_254_ aluminum plates (Merck). Visualization was accomplished by UV light. Column chromatography was performed using silica gel 60 (0.040–0.063 mm) (Merck). Yields of products refer to chromatographically and spectroscopically homogeneous materials. Anhydrous methylene chloride, dimethylformamide and acetonitrile were obtained by distillation over CaH_2_, tetrahydrofuran was obtained by distillation over sodium. Commercial reagents were used as received.

NMR spectra were recorded on Bruker Avance 300 or Bruker Ascend 400 spectrometers (300 MHz, 400 MHz for ^1^H and 75 MHz, 100 MHz for ^13^C, respectively). The proton signals for residual non-deuterated solvents (δ 7.26 ppm for CDCl_3_, δ 2.50 ppm for DMSO-*d*_6_) and carbon signals (δ 77.1 ppm for CDCl_3_, δ 39.5 ppm for DMSO-*d*_6_) were used as an internal reference for ^1^H NMR and ^13^C NMR spectra, respectively. Coupling constants are reported in Hz. Chemical shifts of signals are given in ppm and multiplicity assigned as follows: s – singlet, d – doublet, t – triplet, m – multiplet.

The infrared spectra were recorded on a Perkin Elmer Spectrum BX spectrometer. Wavelengths are given in cm^−1^. The UV–vis absorption spectra of all compounds were acquired using a Perkin-Elmer 35 UV–vis spectrometer. Emission spectra were measured on QuantaMaster 40 steady state spectrofluorometer (Photon Technology International, Inc.). Absolute photoluminescence quantum yields were determined using QuantaMaster 40 steady state spectrofluorometer (Photon Technology International, Inc.) equipped with 6 inch integrating sphere by LabSphere, using a florescence standard of quinine sulfate in 0.1 M H_2_SO_4_ as a reference. High-resolution mass spectrometry (HRMS) analyses were carried out on a Dual-ESI Q-TOF 6520 (Agilent Technologies) mass spectrometer and Agilent 1290 Infinity series UPLC system equipped with column Extend C18 RRHD 2.1 × 50 mm, 1.8 µm connected to an Agilent 6230 TOF LC/MS masspectrometer.

For HPLC analysis we used an Agilent Technologies 1200 Series chromatograph equipped with an Agilent XDB-C18 (4.6 × 50 mm, 1.8 µm) column and a Phenomenex Gemini NX (4.6 × 100 mm, 3 µm) column. Eluent A: 0.01 M KH_2_PO_4_ solution with 6% v/v MeCN added; eluent B: 0.1% TFA solution with 5% v/v MeCN added; eluent C – MeCN.

### General procedures and product characterization

2,6-Bistriazolyl derivative **4** was synthesized using previously reported procedure of Cu(I)-catalyzed azide–alkyne cycloaddition reaction on 2,6-diazidopurine derivatives [25]. Synthesis of 7-deazapurine derivatives **3**, **10a**, **11a** and their characterization are described in our preliminary communication [39].

#### Synthesis of 9-alkyl-2,6-diazido-9*H*-purine derivatives **2a–c**

**Alkylation, method A:** A solution of 2,6-dichloropurine (**1a**, 1.0 g, 5.4 mmol, 1.0 equiv) in anhydrous MeCN or anhydrous DMF (30 mL) was cooled to 0 °C and 57% suspension of NaH (0.3 g, 7.0 mmol, 1.3 equiv) was added in small portions (50 mg). The resulting reaction mixture was stirred for 30 min. After that, the corresponding 1-iodoalkane or 1-bromoalkane (11 mmol, 2.1 equiv) was added and the reaction mixture was stirred for 1–3 days at 20–55 °C. The excess of NaH was neutralized with MeOH or EtOH. The reaction mixture was evaporated under reduced pressure and the residue was dissolved in DCM (30 mL), the organic phase was washed with brine (2 × 15 mL) and subsequently dried over anh. Na_2_SO_4_ and evaporated. Silica gel column chromatography (Hex/EtOAc 4:1) provided the desired product.

**Alkylation method B:** A solution of 2,6-dichloropurine (**1a**, 5.0 g, 26.5 mmol, 1.0 equiv), corresponding alcohol (31.7 mmol, 1.2 equiv) and Ph_3_P (9.2 g, 34.9 mmol, 1.3 equiv) in anhydrous THF (30 mL) was cooled to 0 °C. DIAD (6.90 mL, 35.0 mmol, 1.3 equiv) was added dropwise, the mixture was stirred for 1 h at 20 °C, controlled by HPLC, then evaporated to dryness. Subsequently, EtOH (20 mL) was added and the resulting mixture was cooled to −10 °C to form precipitate of Ph_3_PO, which was filtered as a byproduct and the filtrate was evaporated. The column chromatography (DCM/MeCN 10:1) provided the desired resulting product.

**2,6-Dichloro-9-heptyl-9*H*-purine (1a-1)**: Slightly yellow oil; reaction time (method A) – 1 h; yield 5.0 g, 66%. IR (KBr) ν (cm^−1^): 2933, 1802, 1733; ^1^H NMR (300 MHz, CDCl_3_) δ 8.09 (s, 1H, H-C(8)), 4.23 (t, ^3^*J* = 7.2 Hz, 2H, -CH_2_-), 1.88 (quintet, ^3^*J* = 7.2 Hz, 2H, -CH_2_-), 1.36–1.13 (m, 8H, 4 × -CH_2_-), 0.82 (t, ^3^*J* = 6.8 Hz, 3H, -CH_3_) ppm; ^13^C NMR (75.5 MHz, CDCl_3_) δ 153.3, 152.9, 151.7, 145.9, 130.8, 44.7, 31.6, 29.8, 28.6, 26.6, 22.5, 14.0 ppm; HRMS–ESI (*m*/*z*): [M + H]^+^ calcd for C_12_H_17_Cl_2_N_4_, 287.0825; found, 287.0826.

**Azidation:** NaN_3_ (5.88 g, 90.5 mmol, 3.0 equiv) was added to a solution of 9-alkyl-2,6-dichloro-9*H*-purine (30 mmol, 1.0 equiv) in acetone (50 mL) and stirred for 14 h at 50 °C, protected from the daylight. Then, the reaction mixture was evaporated and suspended in water (30 mL). The resulting precipitate was filtered and dried in vacuum.

**2,6-Diazido-9-heptyl-9*H*-purine (2a)**: Colorless solid; reaction time – 14 h; yield 8.4 g, 93%. IR (KBr) ν (cm^−1^): 2932, 2858, 2170, 2123; ^1^H NMR (300 MHz, CDCl_3_) δ 7.87 (s, 1H, H-C(8)), 4.15 (t, ^3^*J* = 7.2 Hz, 2H, -CH_2_-), 1.93–1.77 (m, 2H, -CH_2_-), 1.39–1.15 (m, 8H, 4 × -CH_2_-), 0.84 (t, ^3^*J* = 6.8 Hz, 3H, -CH_3_) ppm; ^13^C NMR (75.5 MHz, CDCl_3_) δ 155.9, 154.1, 153.7, 143.7, 121.5, 44.2, 31.6, 29.8, 28.7, 26.6, 22.6, 14.1 ppm; HRMS–ESI (*m*/*z*): [M + H]^+^ calcd for C_12_H_17_N_10_, 301.1632; found, 301.1646.

**Synthesis of 9-alkyl-6-azido-2-pyrrolidino-9*H*-purine or 9-alkyl-6-azido-2-piperidino-9*H*-purine derivatives 6a,b:** 9-Alkyl-2,6-diazido-9*H*-purine **2** (8.3 mmol, 1.0 equiv) was dissolved in DMF (30 mL), pyrrolidine or piperidine (11.7 mmol, 1.4 equiv) was added and the reaction mixture was stirred isolated from the daylight at 30 °C for 5 h. After that, the mixture was evaporated and silica gel column chromatography (DCM/MeCN 50:1) was used to provide the desired product.

**6-Azido-9-heptyl-2-pyrrolidino-9*H*-purine (6a)**: Slightly brown solid, reaction time – 4 h; yield 0.88 g, 51%. IR (KBr) ν (cm^−1^): 3062, 2927, 2858, 2148, 2122, 1570, 1254; ^1^H NMR (300 MHz, CDCl_3_) δ 7.56 (s, 1H, H-C(8)), 4.03 (t, ^3^*J* = 7.0 Hz, 2H, -CH_2_-), 3.62–3.54 (m, 4H, 2 × -CH_2_-), 2.00–1.92 (m, 4H, 2 × -CH_2_-), 1.83 (quintet, ^3^*J* = 7.0 Hz, 2H, -CH_2_-), 1.37–1.16 (m, 8H, 4 × -CH_2_-), 0.85 (t, ^3^*J* = 6.8 Hz, 3H, -CH_3_) ppm; ^13^C NMR (75.5 MHz, CDCl_3_) δ 157.4, 154.8, 152.4, 140.3, 117.0, 47.0 (2C)^*^, 43.4, 31.7, 29.6, 28.8, 26.6, 25.6 (2C)^*^, 22.7, 14.1 ppm; HRMS–ESI (*m*/*z*): [M + H]^+^ calcd for C_16_H_25_N_8_, 329.2197; found, 329.2195.

**Synthesis of 9-alkyl-2-pyrrolidino-6-(1,2,3-triazol-1-yl)purine derivatives 7a–f and 9-alkyl-2-piperidino-6-(1,2,3-triazol-1-yl)purine derivatives 8a–f (typical procedure):** Alkyne (1.2 equiv) and 10% AcOH water solution (1 mL) were added to a solution of compound **6a** (200 mg, 0.61 mmol, 1.0 equiv) in THF (7 mL). The flask was isolated from daylight and CuSO_4_∙5H_2_O (10 mol %) and sodium ascorbate (20 mol %) were added. The reaction mixture was heated for 20 h at 50 °C. The reaction mixture was cooled to ambient temperature and the precipitated product (bright yellow/green in color) was filtered. The product on the filter was washed with water (5 mL) and MTBE (3 × 5 mL). Then the product was transferred into a flask and dissolved in CHCl_3_ (7 mL). H_2_S gas was bubbled through the latter solution until dark brown/black suspension appeared. The resulting mixture was filtered through celite, the filtrate was evaporated under reduced pressure and dried in vacuo. Products can be further purified by silica gel column chromatography, if required.

**9-Heptyl-6-(4-phenyl-1*H*-1,2,3-triazol-1-yl)-2-pyrrolidino-9*H*-purine (7a)**: Slightly yellow solid, yield 134 mg, 51%. IR (KBr) ν (cm^−1^): 2952, 2924, 2857, 1622, 1540; ^1^H NMR (300 MHz, 70 °C, DMSO-*d*_6_ + D_2_O) δ 9.36 (s, 1H, H-C(triazole)), 8.29 (s, 1H, H-C(8)), 8.01 (d, ^3^*J* = 7.2 Hz, 2H, Ar), 7.50 (t, ^3^*J* = 7.2 Hz, 2H, Ar), 7.40 (t, ^3^*J* = 7.2 Hz, 1H, Ar), 4.15 (t, ^3^*J* = 7.0 Hz, 2H, -CH_2_-), 3.71–3.55 (m, 4H, 2 × -CH_2_-), 2.08–1.93 (m, 4H, 2 × -CH_2_-), 1.88 (quintet, ^3^*J* = 7.0 Hz, 2H, -CH_2_-), 1.40–1.16 (m, 8H, 4 × -CH_2_-), 0.84 (t, ^3^*J* = 7.0 Hz, 3H, -CH_3_) ppm; ^13^C NMR (75.5 MHz, 70 °C, DMSO-*d*_6_ + D_2_O) δ 156.5, 156.3, 146.4, 143.9, 143.7, 129.6, 128.7, 128.2, 125.5, 120.0, 114.4, 46.6, 42.8, 30.7, 28.4, 27.7, 25.6, 24.7, 21.6, 13.4 ppm; HRMS–ESI (*m*/*z*): [M + Na]^+^ calcd for C_24_H_30_N_8_Na, 453.2478; found, 453.2477.

**Synthesis of 2-dialkylamino-9-methyl-6-[4-(4-substituted phenyl)-1,2,3-triazol-1-yl]-7-deazapurines 10a–f, 11a–f (Analogous as described in** [39]**, a typical procedure):** A mixture of azide **3** (86 mg, 0.4 mmol) and secondary amine (1.2 mmol) in CH_3_CN (2 mL) was protected from daylight and stirred for 8–24 h at 40 °C. After completion of the S_N_Ar reaction (TLC control), the reaction mixture was cooled to rt and the corresponding alkyne (0.52 mmol), DIPEA (70 µL, 0.4 mmol), AcOH (23 µL, 0.4 mmol) and CuI (15 mg, 0.08 mmol) were added. The reaction mixture was stirred under argon atmosphere at rt for 8–10 h (TLC control). Then the reaction mixture was poured into aqueous 10% ammonia solution (25 mL), stirred for 10 minutes and extracted with CHCl_3_ (3 × 20 mL). The combined organic layers were washed with water (2 × 30 mL), dried over anhyd Na_2_SO_4_ and filtered. After removal of the solvent in vacuum, the crude products were purified by silica gel column chromatography (CHCl_3_/EtOAc 6:1) to afford compounds **10a–f**, **11a–f**.

**6-[4-(4-Methoxyphenyl)-1,2,3-triazol-1-yl]-9-methyl-2-pyrrolidino-7-deazapurine (10b)**: Yellow solid, mp 258 °C dec, yield 99 mg, 66%. ^1^H NMR (400 MHz, CDCl_3_) δ 2.01–2.11 (m, 4H, piperidine 2 × CH_2_), 3.65–3.72 (m, 4H, piepridine 2 × CH_2_), 3.74 (s, 3H, CH_3_), 3.88 (s, 3H, OCH_3_), 6.88 (d, *J* = 3.6 Hz, 1H, 6-H), 7.02 (d, *J* = 8.8 Hz, 2H, ArH), 7.08 (d, *J* = 3.6 Hz, 5-H, 1H), 7.92 ( d, *J* = 8.8 Hz, 2H,ArH), 8.79 ( s, 1H,triazole-H); ^13^C NMR (100 MHz, CDCl_3_) δ 25.6, 30.7, 46.9, 55.3, 99.5, 101.6, 114.3, 116.2, 123.1, 126.7, 127.3, 146.7, 147.0, 156.6, 156.8, 159.8; HRMS–ESI (*m*/*z*): [M + H]^+^ calcd for C_20_H_22_N_7_O, 376.1880; found, 376.1887.

### Quantum chemical calculations

The initial molecular geometries were generated by using a molecular mechanics method (force field MMF94, steepest descent algorithm) and systematic conformational analysis as implemented in Avogadro 1.1.1 software. The minimum energy conformers found by molecular mechanics were further optimized with the Gaussian 09 program package [57] by the means of DFT using B3LYP exchange–correlation hybrid functional together with the 6-31G** basis set including the polarizable continuum model in MeCN. Further, harmonic vibrational frequencies were calculated to verify the stability of optimized geometries. All the calculated vibrational frequencies were real (positive), which indicates the true minimum of the total energy on the potential energy hypersurface. The theoretical absorption bands were obtained by the means of time-dependent extension of DFT (TDDFT). Spatial distributions of electron density of calculated HOMOs and LUMOs were obtained at an isosurface value of 0.02 where red and green colors stand for the positive and negative signs of the molecular orbital wave function, respectively.

### Experimental procedure for cell studies

The Human breast adenocarcinoma cell line MCF-7, and normal mammary epithelial cell line MCF10A were ordered from Food Industry Research and Development Institute (Taiwan). MCF-7 cells were cultured in RPMI medium supplemented with fetal bovine serum (10%), antibiotic–antimycotic (1.0%). MCF-10A cells were cultured in α-MEM supplemented with FBS (10%), and antibiotic–antimycotic (1.0%). All cells were cultured in an environment equilibrated with 5% CO_2_ at 37 °C. The cell number and viability of the cells were determined by applying the trypan blue exclusion method and Alamar Blue assay, respectively. Following the separated incubation of MCF-7 and MCF-10A cells (7.0 × 10^4^ cells per well) in a culture medium for 24 h at 37 °C containing 5% CO_2_, each of the culture media were replaced with 500 μL of cell culture medium containing the compound, and then further cultured for an additional 1 h. Those cells were washed twice with 1× PBS and fixed to the membrane using 4% paraformaldehyde in 5.0 mM sodium phosphate buffer (pH 7.4; 1.0 mL) for 10 min. The fluorescence images of cancer cells were acquired with a fluorescence microscope (Olympus IX 71, Center Valley, PA, USA). Light from an Hg lamp (100 W) passed separately through filter (365 ± 20 nm) before it was used to excite the compound. The emission filters used in this study are long pass filters, with cut-on wavelengths at 420 nm (Semrock, Rochester, NY, USA).

## Supporting Information

File 1 Full experimental procedures, emission spectra, DSC data, and copies of ^1^H and ^13^C NMR spectra.
