# Supplementary material for: Synthesis and fluorescent properties of N(9)-alkylated 2-amino-6-triazolylpurines and 7-deazapurines
Source: Beilstein J Org Chem. 2019 Feb 15;15:474–89. doi: 10.3762/bjoc.15.41 (PMC6404417; doi:10.3762/bjoc.15.41)
Supplement: File 1 — Full experimental procedures, emission spectra, DSC data, and copies of 1H and 13C NMR spectra. [file Beilstein_J_Org_Chem-15-474-s001.pdf]

BEILSTEIN JOURNAL OF ORGANIC CHEMISTRY

Supporting Information

for

Synthesis and fluorescent properties of N(9)-alkylated 2-amino-6-triazolylpurines and 7-deazapurines

Andrejs Šišuļins, Jonas Bucevičius, Yu-Ting Tseng, Irina Novosjolova, Kaspars Traskovskis, Ērika Bizdēna, Huan-Tsung Chang, Sigitas Tumkevičius and Māris Turks

Beilstein J. Org. Chem. **2019,** *15,* 474–489. doi:10.3762/bjoc.15.41

Full experimental procedures, emission spectra, DSC data, and copies of ^{1}H and ^{13}C NMR spectra

License and Terms: This is a supporting information file under the terms of the Creative Commons Attribution License (http://creativecommons.org/licenses/by/4.0). Please note that the reuse, redistribution and reproduction in particular requires that the authors and source are credited.
The license is subject to the *Beilstein Journal of Organic Chemistry* terms and conditions: (https://www.beilstein-journals.org/bjoc)

Experimental part

General information

Reactions and purity of the synthesized compounds were monitored by TLC using Silica gel 60 F_{254} aluminum plates (Merck). Visualization was accomplished by UV light. Column chromatography was performed using Silica gel 60 (0.040–0.063 mm) (Merck). Yields of products refer to chromatographically and spectroscopically homogeneous materials. Anhydrous methylene chloride, dimethylformamide and acetonitrile were obtained by distillation over CaH_2, tetrahydrofuran was obtained by distillation over sodium. Commercial reagents were used as received.

NMR spectra were recorded on Bruker Avance 300 or Bruker Ascend 400 spectrometers (300 MHz, 400 MHz for ^{1}H and 75 MHz, 100 MHz for ^{13}C, respectively). The proton signals for residual non-deuterated solvents (δ 7.26 ppm for $CDCl_3$, δ 2.50 ppm for DMSO-d_6) and carbon signals (δ 77.1 ppm for $CDCl_3$, δ 39.5 ppm for DMSO-d_6) were used as an internal reference for ^{1}H NMR and ^{13}C NMR spectra, respectively. Coupling constants are reported in Hz. Chemical shifts of signals are given in ppm and multiplicity assigned as follows: s – singlet, d – doublet, t – triplet, m – multiplet.

The infrared spectra were recorded on Perkin Elmer Spectrum BX. Wavelengths are given in cm^{-1}. The UV–vis absorption spectra of all compounds were acquired using Perkin-Elmer 35 UV–vis spectrometer. Emission spectra were measured on QuantaMaster 40 steady state spectrofluorometer (Photon Technology International, Inc.). Absolute photoluminescence quantum yields were determined using QuantaMaster 40 steady state spectrofluorometer (Photon Technology International, Inc.) equipped with 6 inch integrating sphere by LabSphere, using a florescence standard of quinine sulfate in 0.1 M H_2SO_4 as a reference. High resolution mass spectrometry (HRMS) analyses were carried out on a Dual-ESI Q-TOF 6520 (Agilent Technologies) mass spectrometer and Agilent 1290 Infinity series UPLC system equipped with column Extend C18 RRHD 2.1×50 mm, 1.8 µm connected to Agilent 6230 TOF LC/MS masspectrometer.

For HPLC analysis we used Agilent Technologies 1200 Series chromatograph equipped with Agilent XDB-C18 (4.6 × 50 mm, 1.8 μm) column and Phenomenex Gemini NX (4.6 × 100 mm, 3 μm) column. Eluent A: 0.01 M KH_2PO_4 solution with 6% v/v MeCN added; eluent B: 0.1 % TFA solution with 5% v/v MeCN added; eluent C – MeCN.

General procedures and product characterization.

2,6-Bistriazolyl derivative **4** was synthesized using previously reported procedure of Cu(I)-catalyzed azide-alkyne cycloaddition reaction on 2,6-diazidopurine derivatives [1]. Synthesis of 7-deazapurine derivatives **3**, **10a**, **11a** and their characterization are described in our preliminary communication [2].

Synthesis of 9-alkyl-2,6-diazido-9H-purine derivatives 2a-c

Alkylation

Method A: A solution of 2,6-dichloropurine **1a** (1.0 g, 5.4 mmol, 1.0 equiv.) in anhydrous MeCN or anhydrous DMF (30 mL) was cooled to 0 °C and 57% suspension of NaH (0.3 g, 7.0 mmol, 1.3 equiv) was added in small portions (50 mg). The resulting reaction mixture was stirred for 30 min. After that, the corresponding 1-iodo-alkane or 1-bromo-alkane (11 mmol, 2.1 equiv) was added and the reaction mixture was stirred for 1–3 days at 20–55 °C. The excess of NaH was neutralized with MeOH or EtOH. The reaction mixture was evaporated under the reduced pressure and the residue was dissolved in DCM (30 mL), the organic phase was washed with brine (2 × 15 mL) and subsequently dried over anh. Na_2SO_4 and evaporated. Silica gel column chromatography (Hex/EtOAc 4:1) provided desired product.

Method B: A solution of 2,6-dichloropurine **1a** (5.0 g, 26.5 mmol, 1.0 equiv), corresponding alcohol (31.7 mmol, 1.2 equiv) and Ph_3P (9.2 g, 34.9 mmol, 1.3 equiv) in anhydrous THF (30 mL) was cooled to 0 °C. DIAD (6.90 mL, 35.0 mmol, 1.3 equiv) was added dropwise, the mixture was stirred for 1 h at 20 °C, controlled by HPLC, then evaporated to dryness. Subsequently, EtOH (20 mL) was added and

the resulting mixture was cooled to −10 °C to form precipitate of Ph_3PO, which was filtered as a by-product and the filtrate was evaporated. The column chromatography (DCM/MeCN 10:1) provided the desired resulting product.

2,6-Dichloro-9-heptyl-9*H*-purine (1a-1): slightly yellow oil; reaction time (method A) – 1 h; yield 5.0 g, 66%. IR (KBr) ν (cm^{-1}): 2933, 1802, 1733. ^{1}H NMR (300 MHz, $CDCl_3$) δ (ppm): 8.09 (s, 1H, H-C(8)), 4.23 (t, 2H, ^3J = 7.2 Hz, (-CH_2-)), 1.88 (quintet, 2H, ^3J = 7.2 Hz, (-CH_2-)), 1.36–1.13 (m, 8H, 4×(-CH_2-)), 0.82 (t, 3H, ^3J = 6.8 Hz, (-CH_3)). ^{13}C NMR (75.5 MHz, $CDCl_3$) δ (ppm): 153.3, 152.9, 151.7, 145.9, 130.8, 44.7, 31.6, 29.8, 28.6, 26.6, 22.5, 14.0. HRMS (ESI): calcd for [$C_{12}H_{16}Cl_2N_4$+H^+] 287.0825, found 287.0826.

2,6-Dichloro-9-nonyl-9*H*-purine (1a-2): colorless solid; reaction time (method B) – 24 h; mp 42–43 ^{o}C; yield 3.5 g, 52%. IR (KBr) ν (cm^{-1}): 2963, 2923, 2852, 1811. ^{1}H NMR (300 MHz, DMSO-d_6) δ (ppm): 8.75 (s, 1H, H-C(8)), 4.24 (t, 2H, ^3J = 7.1 Hz, (-CH_2-)), 1.83 (quintet, 2H, ^3J = 7.1 Hz, (-CH_2-)), 1.36 – 1.10 (m, 12H, 6×(-CH_2-)), 0.83 (t, 3H, ^3J = 7.1 Hz, (-CH_3)). ^{13}C NMR (75.5 MHz, DMSO-d_6) δ (ppm): 153.4, 150.8, 149.5, 148.4, 130.4, 43.9, 31.2, 28.8, 28.7, 28.5, 28.3, 25.9, 22.0, 13.8. HRMS (ESI): calcd for [$C_{14}H_{20}Cl_2N_4$+H^+] 315.1138, found 315.1138.

2,6-Dichloro-9-dodecyl-9*H*-purine (1a-3): yellowish solid; reaction time (method B) – 72 h; mp 63–65 ^{o}C; yield 4.5 g, 48%. IR (KBr) ν (cm^{-1}): 2955, 2917, 2850, 1736. ^{1}H NMR (300 MHz, $CDCl_3$) δ (ppm): 8.09 (s, 1H, H-C(8)), 4.25 (t, 2H, ^3J = 7.2 Hz, (-CH_2-)), 1.91 (quintet, 2H, ^3J = 7.2 Hz, (-CH_2-)), 1.39–1.17 (m, 18H, 9×(-CH_2-)), 0.87 (t, 3H, ^3J = 7.0 Hz, (-CH_3)). ^{13}C NMR (75.5 MHz, $CDCl_3$) δ (ppm): 153.3, 153.0, 151.8, 145.9, 130.9, 44.8, 32.0, 29.9, 29.7 (2C)*, 29.6, 29.5, 29.4, 29.0, 26.7, 22.8, 14.2. HRMS (ESI): calcd for [$C_{17}H_{26}Cl_2N_4$+Na^+] 379.1432, found 379.139.

*This signal was assigned from HSQC spectrum.

Azidation

NaN_3 (5.88 g, 90.5 mmol, 3.0 equiv) was added to a solution of 9-alkyl-2,6-dichloro-9*H*-purine (30 mmol, 1.0 equiv) in acetone (50 mL) and stirred for 14 h at 50 °C, protected from the daylight. Then, the reaction mixture was evaporated and suspended in water (30 mL). The resulting precipitate was filtered and dried in vacuum.

2,6-Diazido-9-heptyl-9*H*-purine (2a): colorless solid; reaction time – 14 h; yield 8.4 g, 93%. IR (KBr) ν (cm^{-1}): 2932, 2858, 2170, 2123. ^{1}H NMR (300 MHz, $CDCl_3$) δ (ppm): 7.87 (s, 1H, H-C(8)), 4.15 (t, 2H, ^3J = 7.2 Hz, (-CH_2-)), 1.93–1.77 (m, 2H, (-CH_2-)), 1.39–1.15 (m, 8H, 4×(-CH_2-)), 0.84 (t, 3H, ^3J = 6.8 Hz, (-CH_3)). ^{13}C NMR (75.5 MHz, $CDCl_3$) δ (ppm): 155.9, 154.1, 153.7, 143.7, 121.5, 44.2, 31.6, 29.8, 28.7, 26.6, 22.6, 14.1. HRMS (ESI): calcd for [$C_{12}H_{16}N_{10}+H^+$] 301.1632, found 301.1646.

2,6-Diazido-9-nonyl-9*H*-purine (2b): colorless solid, reaction time – 2 h, mp 72–74 °C; yield 1.5 g, 75%. IR (KBr) ν (cm^{-1}): 2922, 2852, 2166, 2133. ^{1}H NMR (300 MHz, DMSO-d_6) δ (ppm): 8.45 (s, 1H, H-C(8)), 4.15 (t, 2H, ^3J = 7.1 Hz, (-CH_2-)), 1.80 (quintet, 2H, ^3J = 7.1 Hz, (-CH_2-)), 1.34–1.10 (m, 12H, 6×(-CH_2-)), 0.83 (t, 3H, ^3J = 7.1 Hz, (-CH_3)). ^{13}C NMR (75.5 MHz, DMSO-d_6) δ (ppm): 154.4, 153.8, 152.3, 145.7, 121.0, 43.4, 31.2, 28.8, 28.7, 28.5, 28.3, 25.8, 22.0, 13.8. HRMS (ESI): calcd for [$C_{14}H_{20}N_{10}+H^+$] 329.1945, found 329.1948.

2,6-Diazido-9-dodecyl-9*H*-purine (2c): slightly yellow solid, reaction time – 1 h; yield 10.2 g, 66%. IR (KBr) ν (cm^{-1}): 2922, 2849, 2170, 2124. ^{1}H NMR (300 MHz, $CDCl_3$) δ (ppm): 7.87 (s, 1H, H-C(8)), 4.15 (t, 2H, ^3J = 7.2 Hz, (-CH_2-)), 1.85 (quintet, 2H, ^3J = 7.2 Hz, (-CH_2-)), 1.34–1.18 (m, 18H, 9×(-CH_2-)), 0.85 (t, 3H, ^3J = 7.0 Hz, (-CH_3)). ^{13}C NMR (75.5 MHz, $CDCl_3$) δ (ppm): 155.9, 154.1, 153.7, 143.7, 121.5, 44.2, 32.0, 29.8, 29.6 (2C)†, 29.5, 29.4, 29.3, 29.0, 26.6, 22.7, 14.2. HRMS (ESI): calcd for [$C_{17}H_{26}N_{10}+H^+$] 371.2415, found 371.2436.

† This signal was assigned from HSQC spectrum.

Synthesis of 9-alkyl-6-amino-2-triazolylpurine derivative 5

Formation of 2,6-bis(triazolyl)purine derivative

2,6-Bis(4-phenyl-1*H*-1,2,3-triazol-1-yl)-9-heptyl-9*H*-purine (4). Phenylacetylene (0.35 mL, 3.19 mmol, 3.2 equiv) and 10% aqueous solution of AcOH (1 mL) were added to a solution of diazide **2a** (0.30 g, 1.00 mmol, 1.0 equiv) in THF (15 mL). The flask was protected from the daylight and $CuSO_4 \cdot 5H_2O$ (75 mg, 0.30 mmol, 30 mol %) and sodium ascorbate (120 mg, 0.61 mmol, 60 mol %) were added. The reaction mixture was stirred for 1.5 h at 50 °C. Then the mixture was evaporated under reduced pressure to dryness and the residue was suspended in 5% aqueous solution of EDTA (50 mL). The resulting suspension was filtered and the solid on the filter was washed with water (10 mL). The obtained crude product was further purified by silica gel column chromatography (MeCN/DCM gradient 5%→10%) and yielded compound **4** (278 mg, 55%) as a colorless solid. IR (KBr) ν (cm^{-1}): 2929, 2857, 1617, 1587. ^{1}H NMR (300 MHz, $CDCl_3$) δ (ppm): 9.31 (s, 1H, H-C(triazole)), 8.94 (s, 1H, H-C(triazole)), 8.30 (s, 1H, H-C(8)), 8.07–7.97 (2d, 4H, ^3J = 8.2 Hz, Ar), 7.55 – 7.45 (m, 4H, Ar), 7.45–7.35 (m, 2H, Ar), 4.45 (t, 2H, ^3J = 7.2 Hz, (-CH_2-)), 2.04 (quintet, 2H, ^3J = 7.2 Hz, (-CH_2-)), 1.48–1.35 (m, 8H, 4×(-CH_2-)), 0.88 (t, 3H, ^3J = 6.9 Hz, (-CH_3)). ^{13}C NMR (75.5 MHz, $CDCl_3$) δ (ppm): 155.9, 148.7, 148.4, 148.2, 147.3, 145.4, 129.9, 129.6, 129.1, 129.0, 128.8 (2C)[‡], 126.4, 126.2, 122.2, 119.6, 119.0, 44.9, 31.7, 29.9, 28.8, 26.7, 22.6, 14.1. HRMS (ESI): calcd for [$C_{28}H_{28}N_{10}+Na^+$] 527.2396, found 527.2391.

C(6)-Selective S_NAr reaction on 2,6-bis(triazolyl)purine derivative

9-Heptyl-2-(4-phenyl-1*H*-1,2,3-triazol-1-yl)-6-piperidino-9*H*-purine (5). Piperidine (0.10 mL, 1.01 mmol, 3.4 equiv) was added to a solution of 2,6-bis(triazolyl)derivative **4** (0.15 g, 0.30 mmol, 1.0 equiv) in DMF (5 mL) and the resulting reaction mixture was stirred for 1 h at 20 °C. Then it was evaporated to dryness, the residue was dissolved in DCM (20 mL) and the organic phase was washed

‡ This signal was assigned from HSQC spectrum.

with brine (3 × 5 mL) and water (5 mL). The organic phase was dried over anh. Na_2SO_4, filtered and evaporated under reduced pressure. The obtained crude product was further purified by silica gel column chromatography (DCM/MeCN 20:1) and yielded compound **5** (101 mg, 72%) as a colorless solid. IR (KBr) ν (cm^{-1}): 2931, 2855, 1597. ^1H NMR (300 MHz, 70 ^oC, DMSO-d_6) δ (ppm): 9.10 (s, 1H, H-C(triazole)), 8.19 (s, 1H, H-C(8)), 8.00 (d, 2H, ^3J = 7.7 Hz, Ar), 7.47 (t, 2H, ^3J = 7.7 Hz, Ar), 7.37 (t, 1H, ^3J = 7.4 Hz, Ar), 4.38 – 4.25 (br s, 4H, 2×(-CH_2-)), 4.21 (t, 2H, ^3J = 7.2 Hz, (-CH_2-)), 1.88 (quintet, 2H, ^3J = 7.0 Hz, (-CH_2-)), 1.78–1.59 (m, 6H, 3×(-CH_2-)), 1.36–1.15 (m, 8H, 4×(-CH_2-)), 0.82 (t, 3H, ^3J = 7.0 Hz, (-CH_3)). ^{13}C NMR (75.5 MHz, 70 °C, DMSO-d_6) δ (ppm): 153.0, 151.0, 148.3, 146.0, 140.2, 130.0, 128.4, 127.7, 125.3, 119.3, 118.2, 45.6, 42.8, 30.7, 28.8, 27.6, 25.6, 25.3, 23.7, 21.5, 13.3. HRMS (ESI): calcd for $[C_{25}H_{32}N_8+H^+]$ 445.2823, found 445.2820, calcd for $[C_{25}H_{32}N_8+Na^+]$ 467.2648, found 467.2642.

Synthesis of 9-alkyl-6-azido-2-pyrrolidino-9*H*-purine or 9-alkyl-6-azido-2- piperidino-9*H*-purine derivatives 6a,b

9-Alkyl-2,6-diazido-9*H*-purine **2** (8.3 mmol, 1.0 equiv) was dissolved in DMF (30 mL), pyrrolidine or piperidine (11.7 mmol, 1.4 equiv) was added and the reaction mixture was stirred isolated from the daylight at 30 °C for 5 h. After that, the mixture was evaporated and silica gel column chromatography (DCM/MeCN 50:1) was used to provide the desired product.

6-Azido-9-heptyl-2-pyrrolidino-9*H*-purine (6a): slightly brown solid, reaction time – 4 h; yield 0.88 g, 51%. IR (KBr) ν (cm^{-1}): 3062, 2927, 2858, 2148, 2122, 1570, 1254. ^1H NMR (300 MHz, $CDCl_3$) δ (ppm): 7.56 (s, 1H, H-C(8)), 4.03 (t, 2H, ^3J = 7.0 Hz, (-CH_2-)), 3.62–3.54 (m, 4H, 2×(-CH_2-)), 2.00 – 1.92 (m, 4H, 2×(-CH_2-)), 1.83 (quintet, 2H, ^3J = 7.0 Hz, (-CH_2-)), 1.37– 1.16 (m, 8H, 4×(-CH_2-)), 0.85 (t, 3H, ^3J = 6.8 Hz, (-CH_3)). ^{13}C NMR (75.5 MHz, $CDCl_3$) δ (ppm): 157.4, 154.8, 152.4, 140.3, 117.0,

47.0 (2C)*, 43.4, 31.7, 29.6, 28.8, 26.6, 25.6 (2C)*, 22.7, 14.1. HRMS (ESI): calcd for [$C_{16}H_{24}N_8$+H^+] 329.2197, found 329.2195.

6-Azido-9-heptyl-2-piperidino-9*H*-purine (6b): slightly brown solid, reaction time – 5 h; yield 1.8 g, 62%. IR (KBr) ν (cm^{-1}): 2933, 2856, 2121, 1621, 1572. ^{1}H NMR (300 MHz, $CDCl_3$) δ (ppm): 7.58 (s, 1H, H-C(8)), 4.03 (t, 2H, ^3J = 7.0 Hz, (-CH_2-)), 3.87–3.74 (m, 4H, 2×(-CH_2-)), 1.83 (quintet, 2H, ^3J = 7.1 Hz, (-CH_2-)), 1.74–1.55 (m, 6H, 3×(-CH_2-)), 1.39– 1.16 (m, 8H, 4×(-CH_2-)), 0.86 (t, 3H, ^3J = 6.8 Hz, (-CH_3)). ^{13}C NMR (75.5 MHz, $CDCl_3$) δ (ppm): 158.6, 154.9, 152.6, 140.8, 117.2, 45.5, 43.4, 31.7, 29.7, 28.8, 26.6, 25.8, 25.0, 22.7, 14.1. HRMS (ESI): calcd for [$C_{17}H_{26}N_8$+H^+] 343.2353, found 343.2371.

Synthesis of 9-alkyl-2-pyrrolidino-6-(1,2,3-triazol-1-yl)purine derivatives 7a–f

Alkyne (1.2 equiv) and 10% AcOH water solution (1 mL) were added to a solution of compound **6a** (200 mg, 0.61 mmol, 1.0 equiv) in THF (7 mL). The flask was isolated from daylight and $CuSO_4·5H_2O$ (10 mol %) and sodium ascorbate (20 mol %) were added. The reaction mixture was heated for 20 h at 50 °C. The reaction mixture was cooled to ambient temperature and the precipitated product (bright yellow/green in color) was filtered. The product on the filter was washed with water (5 mL) and MTBE (3 × 5 mL). Then the product was transferred into a flask and dissolved in $CHCl_3$ (7 mL). H_2S gas was bubbled through the latter solution until a dark brown/black suspension appeared. The resulting mixture was filtered through celite, the filtrate was evaporated under reduced pressure and dried in vacuo. Products can be further purified by silica gel column chromatography, if required.

9-Heptyl-6-(4-phenyl-1*H*-1,2,3-triazol-1-yl)-2-pyrrolidino-9*H*-purine (7a): slightly yellow solid, yield 134 mg, 51%. IR (KBr) ν (cm^{-1}): 2952, 2924, 2857, 1622, 1540. ^{1}H NMR (300 MHz, 70 °C, DMSO-d_6 + D_2O) δ (ppm): 9.36 (s, 1H, H-C(triazole)), 8.29 (s, 1H, H-C(8)), 8.01 (d, 2H, ^3J = 7.2 Hz,

Ar), 7.50 (t, 2H, ^3J = 7.2 Hz, Ar), 7.40 (t, 1H, ^3J = 7.2 Hz, Ar), 4.15 (t, 2H, ^3J = 7.0 Hz, (-CH_2-)), 3.71–3.55 (m, 4H, 2×(-CH_2-)), 2.08–1.93 (m, 4H, 2×(-CH_2-)), 1.88 (quintet, 2H, ^3J = 7.0 Hz, (-CH_2-)), 1.40–1.16 (m, 8H, 4×(-CH_2-)), 0.84 (t, 3H, ^3J = 7.0 Hz, (-CH_3)). ^{13}C NMR (75.5 MHz, 70 °C, DMSO-d_6 + D_2O) δ (ppm): 156.5, 156.3, 146.4, 143.9, 143.7, 129.6, 128.7, 128.2, 125.5, 120.0, 114.4, 46.6, 42.8, 30.7, 28.4, 27.7, 25.6, 24.7, 21.6, 13.4. HRMS (ESI): calcd for [$C_{24}H_{30}N_8$+Na^+] 453.2478, found 453.2477.

9-Heptyl-6-(4-(4-methoxyphenyl)-1*H*-1,2,3-triazol-1-yl)-2-pyrrolidino-9*H*-purine (7b): slightly yellow solid, yield 160 mg, 57%. IR (KBr) ν (cm^{-1}): 2955, 2927, 2866, 1621, 1559, 1544. ^{1}H NMR (300 MHz, $CDCl_3$) δ (ppm): 8.99 (s, 1H, H-C(triazole)), 7.92 (d, 2H, ^3J = 8.7 Hz, Ar), 7.79 (s, 1H, H-C(8)), 7.00 (d, 2H, ^3J = 8.7 Hz, Ar), 4.13 (t, 2H, ^3J = 7.1 Hz, (-CH_2-)), 3.85 (s, 3H, (-CH_3)), 3.75–3.63 (m, 4H, 2×(-CH_2-)), 2.10–1.97 (m, 4H, 2×(-CH_2-)), 1.96–1.83 (m, 2H, (-CH_2-)), 1.39–1.19 (m, 8H, 4×(-CH_2-)), 0.87 (t, 3H, ^3J = 7.0 Hz, (-CH_3)). ^{13}C NMR (75.5 MHz, $CDCl_3$) δ (ppm): 159.9, 157.3, 157.0, 147.3, 145.1, 142.4, 127.6, 123.2, 118.3, 115.6, 114.3, 55.5, 47.3, 43.6, 31.7, 29.6, 28.8, 26.7, 25.7, 22.7, 14.1. HRMS (ESI): calcd for [$C_{25}H_{32}N_8O$+Na^+] 483.2571, found 483.2589.

9-Heptyl-6-(4-(4-(dimethylamino)phenyl)-1*H*-1,2,3-triazol-1-yl)-2-pyrrolidino-9*H*-purine (7c): yellow solid, yield 153 mg, 53%. IR (KBr) ν (cm^{-1}): 2947, 2924, 2852, 1621, 1563, 1544. ^{1}H NMR (300 MHz, $CDCl_3$) δ (ppm): 8.96 (s, 1H, H-C(triazole)), 7.90 (d, 2H, ^3J = 8.6 Hz, Ar), 7.80 (s, 1H, H-C(8)), 6.93 (d, 2H, ^3J = 8.6 Hz, Ar), 4.13 (t, 2H, ^3J = 7.0 Hz, (-CH_2-)), 3.76 – 3.64 (m, 4H, 2×(-CH_2-)), 3.02 (s, 6H, 2×(-CH_3)), 2.09–1.97 (m, 4H, 2×(-CH_2-)), 1.90 (quintet, 2H, ^3J = 7.0 Hz, (-CH_2-)), 1.41–1.19 (m, 8H, 4×(-CH_2-)), 0.87 (t, 3H, ^3J = 7.0 Hz, (-CH_3)). ^{13}C NMR (75.5 MHz, $CDCl_3$) δ (ppm): 157.3, 156.9, 149.8, 147.7, 145.1, 142.4, 127.4, 120.2, 117.8, 115.6, 113.6, 47.3, 43.6, 41.3, 31.7, 29.7, 28.8, 26.7, 25.7, 22.7, 14.2. HRMS (ESI): calcd for [$C_{26}H_{35}N_9$+Na^+] 496.2897, found 496.2901.

6-(4-(4-Fluorophenyl)-1*H*-1,2,3-triazol-1-yl)-9-heptyl-2-pyrrolidino-9*H*-purine (7d): slightly yellow solid, yield 174 mg, 64%. IR (KBr) ν (cm^{-1}): 2957, 2927, 2858, 1625, 1559, 1540. ^{1}H NMR

(300 MHz, DMSO-d_6) δ (ppm): 9.37 (s, 1H, H-C(triazole)), 8.22 (s, 1H, H-C(8)), 8.05 (dd, 2H, $^3J_{H-H}$ = 8.6 Hz, $^4J_{H-F}$ = 5.5 Hz, Ar), 7.31 (t, 2H, $^3J_{H-H}$ = $^3J_{H-F}$ = 8.6 Hz, Ar), 4.15 (t, 2H, ^3J = 7.0 Hz, (-CH_2-)), 3.64 (t, 4H, ^3J = 6.5 Hz, 2×(-CH_2-)), 2.06–1.95 (m, 4H, 2×(-CH_2-)), 1.89 (quintet, 2H, ^3J = 7.0 Hz, (-CH_2-)), 1.40–1.18 (m, 8H, 4×(-CH_2-)), 0.85 (t, 3H, ^3J = 7.0 Hz, (-CH_3)). ^{13}C NMR (75.5 MHz, DMSO-d_6) δ (ppm): 161.9 (D, $^1J_{C-F}$ = 245 Hz), 156.4, 156.3, 145.3, 143.9, 143.7, 127.5 (D, $^3J_{C-F}$ = 8 Hz), 126.2 (D, $^4J_{C-F}$ = 3 Hz), 120.0, 115.5 (D, $^2J_{C-F}$ = 21 Hz), 115.1, 46.5, 42.5, 30.6, 28.3, 27.6, 25.5, 24.6, 21.5, 13.3. HRMS (ESI): calcd for [$C_{24}H_{29}FN_8$+Na^+] 471.2383, found 471.2384.

6-(4-(4-(Trifluoromethyl)phenyl)-1*H*-1,2,3-triazol-1-yl)-9-heptyl-2-pyrrolidino-9*H*-purine (7e): slightly yellow solid, yield 230 mg, 76%. IR (KBr) ν (cm^{-1}): 2949, 2928, 2867, 1630, 1566, 1542. ^{1}H NMR (300 MHz, $CDCl_3$) δ (ppm): 9.17 (s, 1H, H-C(triazole)), 8.12 (d, 2H, ^3J = 8.0 Hz, Ar), 7.82 (s, 1H, H-C(8)), 7.72 (d, 2H, ^3J = 8.0 Hz, Ar), 4.15 (t, 2H, ^3J = 7.0 Hz, (-CH_2-)), 3.80–3.62 (m, 4H, 2×(-CH_2-)), 2.12–1.98 (m, 4H, 2×(-CH_2-)), 1.91 (quintet, 2H, ^3J = 7.0 Hz, (-CH_2-)), 1.42–1.18 (m, 8H, 4×(-CH_2-)), 0.88 (t, 3H, ^3J = 7.0 Hz, (-CH_3)). ^{13}C NMR (75.5 MHz, $CDCl_3$) δ (ppm): 157.2, 157.1, 146.1, 144.8, 142.7, 133.9, 130.1 (T, $^2J_{C-F}$ = 33 Hz), 126.4, 125.9 (Q, $^3J_{C-F}$ = 4 Hz), 124.3 (D, $^1J_{C-F}$ = 272 Hz), 120.1, 115.6, 47.3, 43.7, 31.7, 29.6, 28.8, 26.7, 25.7, 22.7, 14.2. HRMS (ESI): calcd for [$C_{25}H_{29}F_3N_8$+Na^+] 521.2362, found 521.2357.

6-(4-(4-Cyanophenyl)-1*H*-1,2,3-triazol-1-yl)-9-heptyl-2-pyrrolidino-9*H*-purine (7f): slightly yellow solid, yield 175 mg, 63%. IR (KBr) ν (cm^{-1}): 2960, 2928, 2854, 2223, 1627, 1562, 1545. ^{1}H NMR (300 MHz, $CDCl_3$) δ (ppm): 9.18 (s, 1H, H-C(triazole)), 8.10 (d, 2H, ^3J = 8.3 Hz, Ar), 7.81 (s, 1H, H-C(8)), 7.73 (d, 2H, ^3J = 8.3 Hz, Ar), 4.15 (t, 2H, ^3J = 7.0 Hz, (-CH_2-)), 3.77–3.60 (m, 4H, 2×(-CH_2-)), 2.12–1.97 (m, 4H, 2×(-CH_2-)), 1.92 (quintet, 2H, ^3J = 7.0 Hz, (-CH_2-)), 1.44–1.18 (m, 8H, 4×(-CH_2-)), 0.87 (t, 3H, ^3J = 7.0 Hz, (-CH_3)). ^{13}C NMR (75.5 MHz, $CDCl_3$) δ (ppm): 157.2, 157.1, 145.6, 144.6, 142.7, 134.8, 132.8, 126.6, 120.6, 118.9, 115.6, 111.8, 47.3, 43.7, 31.7, 29.6, 28.8, 26.7, 25.7, 22.7, 14.1. HRMS (ESI): calcd for [$C_{25}H_{29}N_9$+Na^+] 478.2446, found 478.2442.

Synthesis of 9-alkyl-2-piperidino-6-(1,2,3-triazol-1-yl)purine derivatives 8a-f

Alkyne (0.88 mmol, 1.2 equiv) and 10% AcOH water solution (1 mL) were added to a solution of compound **6b** (250 mg, 0.73 mmol, 1.0 equiv) in THF (7 mL), flask was protected from the daylight and the catalyst – $CuSO_4{\cdot}5H_2O$ (10 mol %) and sodium ascorbate (20 mol %) were added. The reaction was stirred for 8 h at 20 °C and the precipitated product (bright yellow/green in color) was filtered. The product on the filter was washed with water (5 mL) and MTBE (3 × 5 mL). Then the product was transferred into a flask and dissolved in $CHCl_3$ (7 mL). H_2S gas was bubbled through the latter solution until dark brown/black suspension appeared. The resulting mixture was filtered through celite, the filtrate was evaporated under reduced pressure and dried in vacuo. Products can be further purified by silica gel column chromatography, if required.

9-Heptyl-6-(4-phenyl-1*H*-1,2,3-triazol-1-yl)-2-piperidino-9*H*-purine (8a): slightly yellow solid, reaction time – 5 h; yield 230 mg, 71%. IR (KBr) ν (cm^{-1}): 2928, 2855, 1629, 1565, 1539. ^1H NMR (300 MHz, $CDCl_3$) δ (ppm): 9.10 (s, 1H, H-C(triazole)), 8.01 (d, 2H, ^3J = 7.4 Hz, Ar), 7.81 (s, 1H, H-C(8)), 7.46 (t, 2H, ^3J = 7.4 Hz, Ar), 7.36 (t, 1H, ^3J = 7.4 Hz, Ar), 4.13 (t, 2H, ^3J = 7.0 Hz, (-CH_2-)), 3.96 – 3.88 (m, 4H, 2×(-CH_2-)), 1.90 (quintet, 2H, ^3J = 7.0 Hz, (-CH_2-)), 1.76–1.60 (m, 6H, 3×(-CH_2-)), 1.43–1.20 (m, 8H, 4×(-CH_2-)), 0.88 (t, 3H, ^3J = 6.8 Hz, (-CH_3)). ^{13}C NMR (75.5 MHz, $CDCl_3$) δ (ppm): 158.5, 157.0, 147.6, 145.1, 142.8, 130.4, 128.9, 128.5, 126.3, 119.4, 115.7, 45.7, 43.6, 31.7, 29.7, 28.8, 26.7, 25.9, 25.0, 22.7, 14.2. HRMS (ESI): calcd for [$C_{25}H_{32}N_8$+Na^+] 467.2648, found 467.2647.

9-Heptyl-6-(4-(4-methoxyphenyl)-1*H*-1,2,3-triazol-1-yl)-2-piperidino-9*H*-purine (8b): slightly yellow solid, reaction time – 24 h; yield 164 mg, 60%. IR (KBr) ν (cm^{-1}): 2925, 2857, 1627, 1562. ^1H NMR (300 MHz, $CDCl_3$) δ (ppm): 8.99 (s, 1H, H-C(triazole)), 7.92 (d, 2H, ^3J = 8.6 Hz, Ar), 7.80 (s, 1H, H-C(8)), 6.99 (d, 2H, ^3J = 8.6 Hz, Ar), 4.12 (t, 2H, ^3J = 7.1 Hz, (-CH_2-)), 3.95–3.86 (m, 4H, 2×(-CH_2-)), 3.84 (s, 3H, (-CH_3)), 1.88 (quintet, 2H, ^3J = 6.9 Hz, (-CH_2-)), 1.76–1.58 (m, 6H, 3×(-CH_2-)),

1.41–1.17 (m, 8H, 4×(-CH_2-)), 0.87 (t, 3H, ^3J = 6.9 Hz, (-CH_3)). ^{13}C NMR (75.5 MHz, $CDCl_3$) δ (ppm): 159.9, 158.5, 156.9, 147.4, 145.1, 142.8, 127.6, 123.1, 118.4, 115.6, 114.3, 55.5, 45.6, 43.5, 31.7, 29.7, 28.8, 26.7, 25.9, 25.0, 22.7, 14.1. HRMS (ESI): calcd for [$C_{26}H_{34}N_8O$+Na+] 497.2753, found 497.2742.

9-Heptyl-6-(4-(4-(dimethylamino)phenyl)-1*H*-1,2,3-triazol-1-yl)-2-piperidino-9*H*-purine (8c): yellow solid, reaction time – 12 h; yield 188 mg, 53%. IR (KBr) ν (cm^{-1}): 2930, 2853, 1622, 1562. ^{1}H NMR (300 MHz, $CDCl_3$) δ (ppm): 8.94 (s, 1H, H-C(triazole)), 7.88 (d, 2H, ^3J = 8.7 Hz, Ar), 7.79 (s, 1H, H-C(8)), 6.81 (d, 2H, ^3J = 8.7 Hz, Ar), 4.12 (t, 2H, ^3J = 7.0 Hz, (-CH_2-)), 3.99–3.84 (m, 4H, 2×(-CH_2-)), 3.00 (s, 6H, 2×(-CH_3)), 1.89 (quintet, 2H, ^3J = 7.0 Hz, (-CH_2-)), 1.77–1.60 (m, 6H, 3×(-CH_2-)), 1.41–1.20 (m, 8H, 4×(-CH_2-)), 0.88 (t, 3H, ^3J = 6.9 Hz, (-CH_3)). ^{13}C NMR (75.5 MHz, $CDCl_3$) δ (ppm): 158.5, 156.9, 150.7, 148.1, 145.2, 142.6, 127.3, 118.6, 117.6, 115.7, 112.6, 45.7, 43.5, 40.6, 31.7, 29.7, 28.8, 26.7, 25.9, 25.0, 22.7, 14.2. HRMS (ESI): calcd for [$C_{27}H_{37}N_9$+Na^+] 510.3070, found 510.3064.

6-(4-(4-Fluorophenyl)-1*H*-1,2,3-triazol-1-yl)-9-heptyl-2-piperidino-9*H*-purine (8d): slightly yellow solid, reaction time – 16 h; yield 260 mg, 74%. IR (KBr) ν (cm^{-1}): 2930, 2856, 1630, 1566, 1541. ^{1}H NMR (300 MHz, $CDCl_3$) δ (ppm): 9.05 (s, 1H, H-C(triazole)), 8.01–7.91 (dd, 2H, $^3J_{H\text{-}H}$ = 8.6 Hz, $^4J_{H\text{-}F}$ = 5.4 Hz, Ar), 7.81 (s, 1H, H-C(8)), 7.14 (t, 2H, $^3J_{H\text{-}F}$ = $^3J_{H\text{-}H}$ = 8.6 Hz, Ar), 4.13 (t, 2H, ^3J = 7.0 Hz, (-CH_2-)), 3.97–3.84 (m, 4H, 2×(-CH_2-)), 1.90 (quintet, 2H, ^3J = 7.0 Hz, (-CH_2-)), 1.78–1.60 (m, 6H, 3×(-CH_2-)), 1.42–1.18 (m, 8H, 4×(-CH_2-)), 0.88 (t, 3H, ^3J = 6.9 Hz, (-CH_3)). ^{13}C NMR (75.5 MHz, $CDCl_3$) δ (ppm): 163.1 (D, $^1J_{C\text{-}F}$ = 247 Hz), 158.5, 157.0, 146.7, 145.0, 142.9, 128.1 (D, $^3J_{C\text{-}F}$ = 8 Hz), 126.6 (D, $^4J_{C\text{-}F}$ = 3 Hz), 119.1, 115.9 (D, $^2J_{C\text{-}F}$ = 22 Hz), 115.7, 45.7, 43.6, 31.7, 29.7, 28.8, 26.7, 25.9, 25.0, 22.7, 14.2. HRMS (ESI): calcd for [$C_{25}H_{31}FN_8$+Na^+] 485.2553, found 485.2545.

6-(4-(4-(Trifluoromethyl)phenyl)-1*H*-1,2,3-triazol-1-yl)-9-heptyl-2-piperidino-9*H*-purine (8e): yellow solid, reaction time – 12 h; yield 313 mg, 84%. IR (KBr) ν (cm^{-1}): 2930, 2855, 1631, 1571,

1539. ^{1}H NMR (300 MHz, $CDCl_3$) δ (ppm): 9.18 (s, 1H, H-C(triazole)), 8.13 (d, 2H, ^{3}J = 8.0 Hz, Ar), 7.82 (s, 1H, H-C(8)), 7.72 (d, 2H, ^{3}J = 8.0 Hz, Ar), 4.14 (t, 2H, ^{3}J = 6.9 Hz, ($-CH_2-$)), 4.00 – 3.85 (m, 4H, 2×($-CH_2-$)), 1.90 (quintet, 2H, ^{3}J = 6.9 Hz, ($-CH_2-$)), 1.80–1.60 (m, 6H, 3×($-CH_2-$)), 1.44–1.19 (m, 8H, 4×($-CH_2-$)), 0.88 (t, 3H, ^{3}J = 6.9 Hz, ($-CH_3$)). ^{13}C NMR (75.5 MHz, $CDCl_3$) δ (ppm): 158.5, 157.1, 146.3, 144.9, 143.0, 133.9, 130.4 (quartet, $^{2}J_{C-F}$ = 33 Hz, Ar), 126.5, 126.0 (quartet, $^{3}J_{C-F}$ = 4 Hz, Ar), 124.3 (D, $^{1}J_{C-F}$ = 272 Hz, ($-CF_3$)), 120.3, 115.7, 45.7, 43.7, 31.7, 29.7, 28.8, 26.7, 25.9, 25.0, 22.7, 14.1. HRMS (ESI): calcd for [$C_{26}H_{31}F_3N_8$+H^+] 513.2697, found 513.2686.

6-(4-(4-Cyanophenyl)-1*H*-1,2,3-triazol-1-yl)-9-heptyl-2-piperidino-9*H*-purine (8f): slightly yellow solid, reaction time – 8 h; yield 0.3 g, 86%. IR (KBr) ν (cm^{-1}): 2931, 2856, 2225, 1630, 1564, 1541. ^{1}H NMR (300 MHz, $CDCl_3$) δ (ppm): 9.18 (s, 1H, H-C(triazole)), 8.11 (d, 2H, ^{3}J = 8.4 Hz, Ar), 7.81 (s, 1H, H-C(8)), 7.73 (d, 2H, ^{3}J = 8.4 Hz, Ar), 4.13 (t, 2H, ^{3}J = 7.2 Hz, ($-CH_2-$)), 3.96–3.85 (m, 4H, 2×($-CH_2-$)), 1.89 (quintet, 2H, ^{3}J = 6.9 Hz, ($-CH_2-$)), 1.78–1.60 (m, 6H, 3×($-CH_2-$)), 1.42–1.18 (m, 8H, 4×($-CH_2-$)), 0.87 (t, 3H, ^{3}J = 6.9 Hz, ($-CH_3$)). ^{13}C NMR (75.5 MHz, $CDCl_3$) δ (ppm): 158.4, 157.1, 145.7, 144.7, 143.0, 134.8, 132.8, 126.6, 120.7, 118.9, 115.6, 111.8, 45.7, 43.6, 31.7, 29.7, 28.8, 26.7, 25.9, 24.9, 22.7, 14.1. HRMS (ESI): calcd for [$C_{26}H_{31}N_9$+Na^+] 492.2600, found 492.2600.

Synthesis of 9-pentyl-6-(4-phenyl-1*H*-1,2,3-triazol-1-yl)-2-piperidino-9*H*-purine (9)

A solution of 2,6-dichloropurine (**1a**, 1.9 g, 10.0 mmol, 1.0 equiv) in anhydrous MeCN was cooled to 0 ^{o}C, 57% suspension of NaH (0.44 g, 11.0 mmol, 1.1 equiv) was added in small portions (50 mg) and the mixture was stirred for 30 min. 1-Bromopentane (1.24 mL, 10.0 mmol, 1.0 equiv) was added and the resulting mixture was stirred at 50 °C for 2 h. The excess of NaH was neutralized with MeOH. The mixture was filtered and evaporated under reduced pressure. The residue was dissolved in EtOAc (30 mL), the organic phase was washed with water (1 × 15 mL) and brine (2 × 15 mL) and subsequently dried over anh. Na_2SO_4 and evaporated. Silica gel column chromatography (1–10 % EtOH in DCM)

provided desired 2,6-dichloro-9-pentyl-9*H*-purine in 56% yield (1.4 g). Then 2,6-dichloro-9-pentyl-9*H*-purine (0.6 g, 2.3 mmol, 1.0 equiv) was dissolved in EtOH (20 mL), solution of NaN_3 (0.6 g, 9.2 mmol, 4.0 equiv) in water (5 mL) was added and the resulting reaction mixture was stirred at 70 °C for 1 h. Then it was evaporated and the residue was crystalized from water, giving 2,6-diazido-9-pentyl-9*H*-purine (**2d**) in 80% yield (0.5 g). Obtained 2,6-diazido derivative (0.5 g, 1.8 mmol, 1.0 equiv) was dissolved in DMF (5 mL), piperidine (0.34 mL, 2.7 mmol, 1.5 equiv) was added and the mixture was stirred at room temperature for 2.5 h. Then the mixture was poured in water (5 mL) and extracted with EtOAc (1 × 20 mL). The EtOAc layer was washed with brine (3 × 15 mL), subsequently dried over anh. Na_2SO_4 and evaporated. Silica gel column chromatography (toluene/EtOAc 2:1) provided desired 6-azido-9-pentyl-2-piperidino-9*H*-purine (**6c**) in 70 % yield (0.4 g) which was used in the following "click" reaction. Phenylacetylene (0.21 mL, 2.0 mmol, 2.0 equiv) and 10% AcOH water solution (1 mL) were added to a solution of 6-azido-9-pentyl-2-piperidino-9*H*-purine (0.3 g, 1.0 mmol, 1.0 equiv) in DMF (3 mL), flask was protected from the daylight and the catalyst – $CuSO_4{\cdot}5H_2O$ (10 mol %) and sodium ascorbate (20 mol %) were added. The reaction was stirred for 7 h at 90 °C and then the mixture was poured to ice-water (50 mL) and left to precipitate for 12 h in refrigerator. The resulting precipitate was filtered and re-crystalized from EtOH, giving 9-pentyl-6-(4-phenyl-1*H*-1,2,3-triazol-1-yl)-2-piperidino-9*H*-purine (**9**) as a colorless solid, mp = 161–162 ^{o}C; yield 306 mg, 75%. IR (KBr) ν (cm^{-1}): 3140, 2936, 2852, 1753, 1628. ^{1}H NMR (300 MHz, 70 °C, DMSO-d_6) δ (ppm): 9.41 (s, 1H, H-C(triazole)), 8.26 (s, 1H, H-C(8)), 8.03 (d, 2H, ^3J = 7.4 Hz, Ar), 7.51 (t, 2H, ^3J = 7.4 Hz, Ar), 7.41 (t, 1H, ^3J = 7.4 Hz, Ar), 4.16 (t, 2H, ^3J = 7.1 Hz, (-CH_2-)), 3.95–3.80 (m, 4H, 2×(-CH_2-)), 1.89 (quintet, 2H, ^3J = 7.1 Hz, (-CH_2-)), 1.75–1.56 (m, 6H, 3×(-CH_2-)), 1.44–1.23 (m, 4H, 2×(-CH_2-)), 0.88 (t, 3H, ^3J = 7.1 Hz, (-CH_3)). ^{13}C NMR (75.5 MHz, 70 °C, DMSO-d_6) δ (ppm): 157.5, 156.4, 146.3, 144.1, 144.0, 129.6, 128.5, 128.0, 125.4, 120.1, 115.1, 44.7, 42.5, 28.1, 27.8, 24.9, 23.9, 21.0, 13.2. HRMS (ESI):

calcd for [$C_{23}H_{28}N_8+H^+$] 417.2510, found 417.2488 (5.2 ppm), calcd for [$C_{23}H_{28}N_8+Na^+$] 439.2329, found 439.2310.

Synthesis of 2-dialkylamino-9-methyl-6-[4-(4-substituted phenyl)-1,2,3-triazol-1-yl]-7-deazapurines 10a–f, 11a–f (Analogous as described in [2])

A mixture of azide **3** (86 mg, 0.4 mmol) and secondary amine (1.2 mmol) in CH_3CN (2 mL) was protected from daylight and stirred for 8–24 h at 40 °C. After completion of the S_NAr reaction (TLC control), the reaction mixture was cooled to rt and corresponding alkyne (0.52 mmol), DIPEA (70 µL, 0.4 mmol), AcOH (23 µL, 0.4 mmol) and CuI (15 mg, 0.08 mmol) were added. The reaction mixture was stirred under argon atmosphere at rt for 8–10 h (TLC control). Then the reaction mixture was poured into aqueous 10% ammonia solution (25 mL), stirred for 10 minutes and extracted with $CHCl_3$ (3 × 20 mL). The combined organic layers were washed with water (2 × 30 mL), dried over anhyd Na_2SO_4 and filtered. After removal of the solvent in vacuum, the crude products were purified by silica gel column chromatography ($CHCl_3$ – EtOAc, 6:1) to afford compounds **10a–f, 11a–f**.

6-[4-(4-Methoxyphenyl)-1,2,3-triazol-1-yl]-9-methyl-2-pyrrolidino-7-deazapurine (10b): yellow solid, mp 258 ^{o}C dec, yield 99 mg, 66%. ^{1}H NMR (400 MHz, $CDCl_3$): δ 2.01-2.11 (4H, m, piperidine 2xCH_2), 3.65-3.72 (4H, m, piepridine 2xCH_2), 3.74 (3H, s, CH_3), 3.88 (3H, s, OCH_3) , 6.88 (1H, d, *J* = 3.6 Hz, 6-H), 7.02 (2H, d, *J* = 8.8 Hz, ArH), 7.08 (1H, d, *J* = 3.6 Hz, 5-H), 7.92 (2H, d, *J* = 8.8 Hz, ArH), 8.79 (1H, s, triazole-H). ^{13}C NMR (100 MHz, $CDCl_3$): δ 25.6, 30.7, 46.9, 55.3, 99.5, 101.6, 114.3, 116.2, 123.1, 126.7, 127.3, 146.7, 147.0, 156.6, 156.8, 159.8. HRMS (ESI): *m/z* [M+H]$^+$ calculated for $C_{20}H_{22}N_7O$: 376.1880; found 376.1887.

9-Methyl-6-[4-(4-(dimethylamino)phenyl)-1,2,3-triazol-1-yl]-2-pyrrolidino-7-deazapurine (10c): yellow solid, mp 256 ^{o}C dec, yield 106 mg, 68%. ^{1}H NMR (400 MHz, $CDCl_3$): δ 2.05 (4H, m, pyrolidine 2xCH_2), 3.03 (6H, s, $N(CH_3)_2$), 3.70 (4H, m, pyrolidine 2xCH_2), 3.74 (3H, s, CH_3), 6.84

(2H, d, J = 8.8 Hz, ArH), 6.86 (1H, d, J = 3.6 Hz, 6-H), 7.08 (1H, d, J = 3.6 Hz, 5-H), 7.86 (2H, d, J = 8.8 Hz, ArH), 8.73 (1H, s, triazole-H). ^{13}C NMR (100 MHz, $CDCl_3$): δ 25.6, 30.7, 40.5, 46.9, 99.5, 101.7, 112.5, 115.4, 118.6, 126.5, 126.9, 147.1, 147.3, 150.5, 156.6, 156.9. HRMS (ESI): *m/z* $[M+H]^+$ calculated for $C_{21}H_{25}N_8$: 389.2197; found 389.2202.

6-[4-(4-Fluorophenyl)-1,2,3-triazol-1-yl]-9-methyl-2-pyrrolidino-7-deazapurine (10d): yellow solid, mp 197 ^{o}C dec, yield 116 mg, 80%. ^{1}H NMR (400 MHz, $CDCl_3$): δ 2.06 (4H, m, pyrolidine 2xCH_2), 3.70 (4H, m, pyrolidine 2xCH_2), 3.75 (3H, s, CH_3), 6.89 (1H, d, J = 3.6 Hz, 6-H), 7.06 (1H, d, J = 3.6 Hz, 5-H), 7.17 (2H, dd, ^3J = 8.8 Hz, $^3J_{H-F}$ = 8.8 Hz, ArH), 7.96 (2H, dd, ^3J = 8.8 Hz, $^4J_{H-F}$ = 5.2 Hz, ArH), 8.83 (1H, s, triazole-H). ^{13}C NMR (100 MHz, $CDCl_3$): δ 25.5, 30.7, 46.9, 99.5, 101.5, 115.8 (d, $^2J_{C-F}$ = 22 Hz), 116.8, 126.6 (d, $^4J_{C-F}$ = 3 Hz), 126.8, 127.7 (d, $^3J_{C-F}$ = 8 Hz), 145.9, 146.8, 156.7, 156.8, 162.8 (d, $^1J_{C-F}$ = 246 Hz). HRMS (ESI): *m/z* $[M+H]^+$ calculated for $C_{19}H_{19}FN_7$: 364.1680; found 364.1681.

6-{4-[4-(Trifluoromethyl)phenyl]-1,2,3-triazol-1-yl}-9-methyl-2-pyrrolidino-7-deazapurine (10e): yellow solid, mp 246 ^{o}C dec, yield 125 mg, 76%. ^{1}H NMR (400 MHz, $CDCl_3$): δ 2.05-2.08 (4H, m, pyrollidine 2xCH_2), 3.68-3.73 (4H, m, pyrollidine 2xCH_2), 3.76 (3H, s, CH_3), 6.89 (1H, d, J = 3.6 Hz, 6-H), 7.06 (1H, d, J = 3.6 Hz, 5-H), 7.73 (2H, d, J = 8.0 Hz, ArH), 8.09 (2H, d, J = 8.0 Hz), 8.93 (1H, s, triazole-H). ^{13}C NMR (100 MHz, $CDCl_3$): δ 25.5, 30.9, 47.0, 99.5, 101.5, 117.9, 124.1 (q, $^1J_{C-F}$ = 270 Hz), 125.8 (q, $^3J_{C-F}$ = 4 Hz), 126.0, 127.0, 130.2(q, $^2J_{C-F}$ = 32 Hz), 133.7, 145.4, 146.7, 156.4, 156.6. HRMS (ESI): *m/z* $[M+H]^+$ calculated for $C_{20}H_{19}FN_7$: 414.1649; found 414.1649.

6-[4-(4-Cyanophenyl)-1,2,3-triazol-1-yl]-9-methyl-2-pyrrolidino-7-deazapurine (10f): yellow solid, mp 259 ^{o}C dec, yield 117 mg, 79%. ^{1}H NMR (400 MHz, $CDCl_3$): δ 2.01-2.09 (4H, m, pyrolidine 2xCH_2), 3.63-3.71 (4H, m, pyrolidine 2xCH_2), 3.75 (3H, s, CH_3), 6.89 (1H, d, J = 3.6 Hz, 6-H), 7.03 (1H, d, J = 3.6 Hz, 5-H), 7.73 (2H, d, J = 8.4 Hz, ArH), 8.06 (2H, d, J = 8.4 Hz, ArH), 8.93 (1H, s, triazole-H). ^{13}C NMR (100 MHz, $CDCl_3$): δ 25.5, 30.8, 46.9, 99.5, 101.4, 111.6, 118.3, 118.7, 126.2,

127.1, 132.7, 134.7, 144.9, 146.5, 156.74, 156.76. HRMS (ESI): *m/z* $[M+H]^+$ calculated for $C_{20}H_{19}N_8$: 371.1727; found 371.1726.

6-[4-(4-Methoxyphenyl)-1,2,3-triazol-1-yl]-9-methyl-2-piperidino-7-deazapurine (11b): yellow solid, mp 206 ^oC dec, yield 93 mg, 60%. ^1H NMR (400 MHz, $CDCl_3$): δ 1.63-1.78 (6H, m, piperidine 3xCH_2), 3.74 (3H, s, CH_3), 3.88 (3H, s, OCH_3), 3.91 (4H, m, piperidine 2xCH_2), 6.91 (1H, d, J = 3.6 Hz, 6-H), 7.02 (2H, d, J = 9.2 Hz, ArH) 7.07 (1H, d, J = 3.6 Hz, 5-H), 7.93 (2H, d, J = 9.2 Hz, ArH), 8.76 (1H, s, triazole-H). ^{13}C NMR (100 MHz, $CDCl_3$): δ 24.9, 25.7, 30.7, 45.5, 55.3, 99.8, 101.7, 114.3, 116.2, 123.0, 127.1, 127.3, 146.7, 147.1, 156.5, 158.1, 159.8. HRMS (ESI): *m/z* $[M+H]^+$ calculated for $C_{21}H_{24}N_7O$: 390.2037; found 390.2049.

9-Methyl-6-[4-(4-(dimethylamino)phenyl)-1,2,3-triazol-1-yl]-2-piperidino-7-deazapurine (11c): yellow solid, mp 230 ^oC dec, yield 93 mg, 58%. ^1H NMR (400 MHZ, $CDCl_3$): δ 1.65-1.77 (6H, m, piperidine 3xCH_2), 3.03 (6H, s, $N(CH_3)_2$) 3.75 (3H, s, CH_3), 3.91 (4H, m, piperidine 2xNCH_2), 6.84 (d, J = 8.8 Hz, ArH), 6.90 (1H, d, J = 3.6 Hz, 6-H), 7.08 (1H, d, J = 3.6 Hz, 5-H), 7.87 (2H, d, J = 8.8 Hz, ArH), 8.72 (1H, s, triazole-H). ^{13}C NMR (100 MHz, $CDCl_3$): δ 25.0, 25.8, 30.8, 40.5, 45.53, 99.8, 101.9, 112.5, 115.4, 118.5, 126.9, 127.1, 147.30, 147.35, 150.5, 156.5. 158.1. HRMS (ESI): *m/z* $[M+H]^+$ calculated for $C_{22}H_{27}N_8$: 403.2353; found 403.2349.

6-[4-(4-Fluorophenyl)-1,2,3-triazol-1-yl]-9-methyl-2-piperidino-7-deazapurine (11d): yellow solid, mp 191-193 ^oC, yield 102 mg, 68%. ^1H NMR (400 MHz, $CDCl_3$): δ 1.64-1.75 (6H, m, piperidine 3xCH_2), 3.74 (3H, s, CH_3), 3.90 (4H, m, piperidine 2xCH_2), 6.91 (1H, d, J = 3.6 Hz, 6-H), 7.06 (1H, d, J = 3.6 Hz, 5-H), 7.17 (2H, dd, ^3J = 8.8 Hz, $^3J_{H-F}$ = 8.8 Hz, ArH), 7.96 (2H, dd, ^3J = 8.8 Hz, $^4J_{H-F}$ = 5.2 Hz, ArH), 8.81 (1H, s, triazole-H). ^{13}C NMR (100 MHz, $CDCl_3$): δ 24.9, 25.7, 30.8, 45.5, 99.8, 101.6, 115.8 (d, $^2J_{C-F}$ = 22 Hz), 116.8, 126.5 (d, $^4J_{C-F}$ = 3 Hz), 127.3, 127.7 (d, $^3J_{C-F}$ = 9 Hz), 146.0, 147.0, 156.6, 158.1, 162.8 (d, $^1J_{C-F}$ = 246 Hz). HRMS (ESI): *m/z* $[M+H]^+$ calculated for $C_{20}H_{21}FN_7$: 378.1837; found 378.1836.

6-{4-[4-(Trifluoromethyl)phenyl]-1,2,3-triazol-1-yl}-9-methyl-2-piperidino-7-deazapurine (11e): yellow solid, mp 228 °C dec, yield 107 mg, 63%. ^{1}H NMR (400 MHz, $CDCl_3$): δ 1.66-1.78 (6H, m, piperidine 3xCH_2), 3.75 (3H, s, CH_3), 3.91 (4H, m, piperidine 2xCH_2), 6.92 (1H, d, *J* = 3.6 Hz, 6-H), 7.05 (1H, d, *J* = 3.6 Hz, 5-H), 7.74 (2H, d, *J* = 8.4 Hz, ArH), 8.11 (2H, d, *J* = 8.4 Hz, ArH), 8.92 (1H, s, triazole-H). ^{13}C NMR (100 MHz, $CDCl_3$): δ 24.9, 25.7, 30.8, 45.5, 99.8, 101.5, 117.9, 124.1 (q, $^1J_{C-F}$ = 270 Hz), 125.8 (q, $^3J_{C-F}$ = 4 Hz), 126.1, 127.5, 130.2 (q, $^2J_{C-F}$ = 32 Hz), 133.8, 145.4, 146.8, 156.6, 158.0. HRMS (ESI): *m/z* $[M+H]^+$ calculated for $C_{21}H_{21}F_3N_7$: 428.1805; found 428.1806

6-[4-(4-Cyanophenyl)-1,2,3-triazol-1-yl]-9-methyl-2-piperidino-7-deazapurine (11f): orange solid, mp 254 °C dec, yield 115 mg, 75%. ^{1}H NMR (400 MHZ, $CDCl_3$): δ 1.66-1.78 (6H, m, piperidine 3xCH_2), 3.76 (3H, s, CH_3), 3.91 (4H, m, piperidine 2xCH_2), 6.93 (1H, d, *J* = 3.6 Hz, 6-H), 7.04 (1H, d, *J* = 3.6 Hz, 5-H), 7.77 (2H, d, *J* = 8.4 Hz, ArH), 8.11 (2H, d, *J* = 8.4 Hz, ArH), 8.95 (1H, s, triazole-H). ^{13}C NMR (100 MHz, $CDCl_3$): δ 24.9, 27.8, 30.8, 45.5, 99.8, 101.5, 111.7, 118.3, 118.7, 126.3, 127.6, 132.7, 134.7, 145.1, 146.7, 156.7, 158.0. HRMS (ESI): *m/z* $[M+H]^+$ calculated for $C_{21}H_{21}N_8$: 385.1884; found 385.1891.

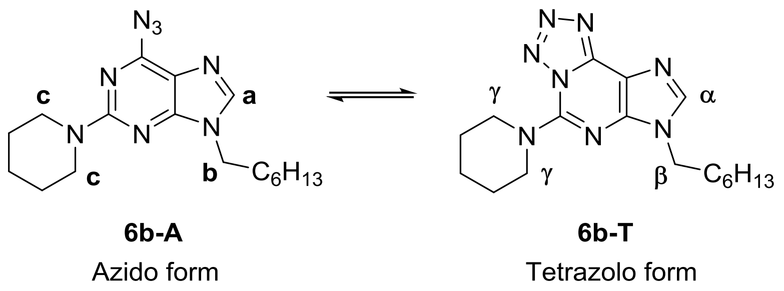

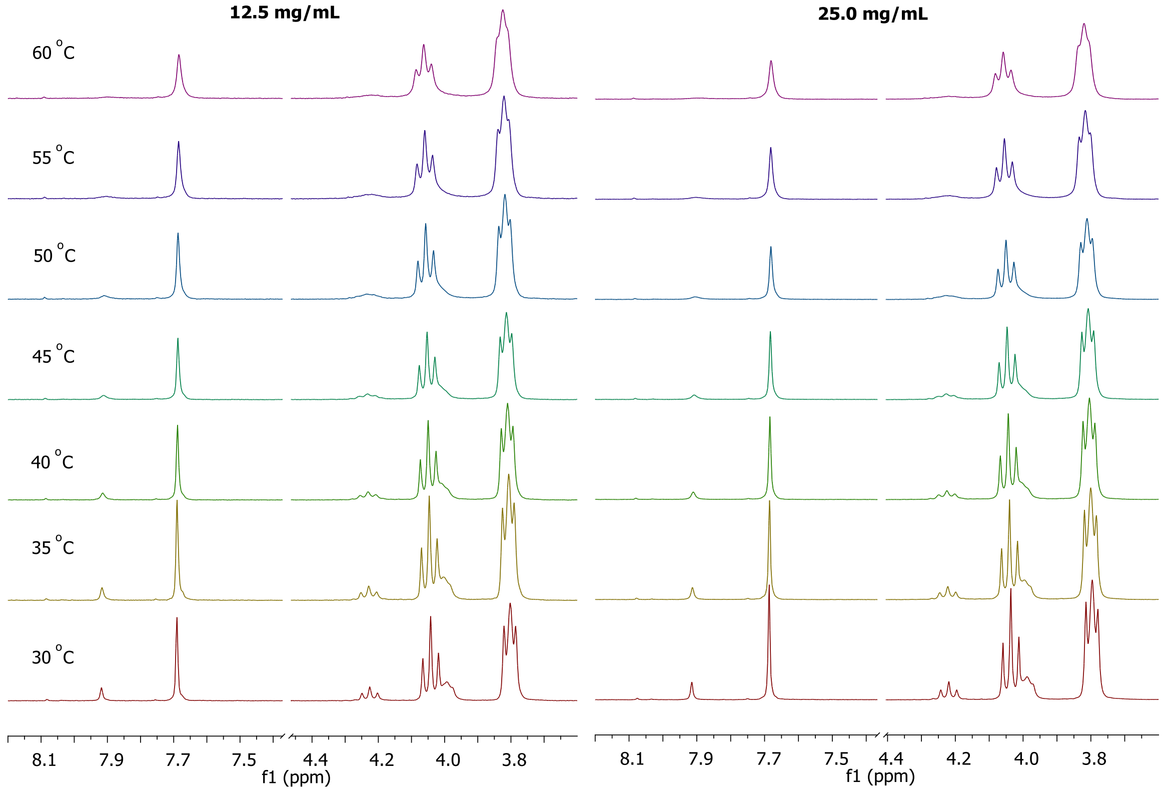

Figure S1: ^{1}H NMR spectra of compound **6b** in CD_3CN in different temperatures (300 MHz).

Table S1: The ratio of tetrazole form in different temperatures for compound **6b**, %.

Temperature, °C	Ratio of tetrazole (7.92 ppm), %	
	12.5 mg/mL	25.0 mg/mL
30	17	16
35	16	15
40	15	14
45	14	12
50	12	10
55	11	8
60	8	6

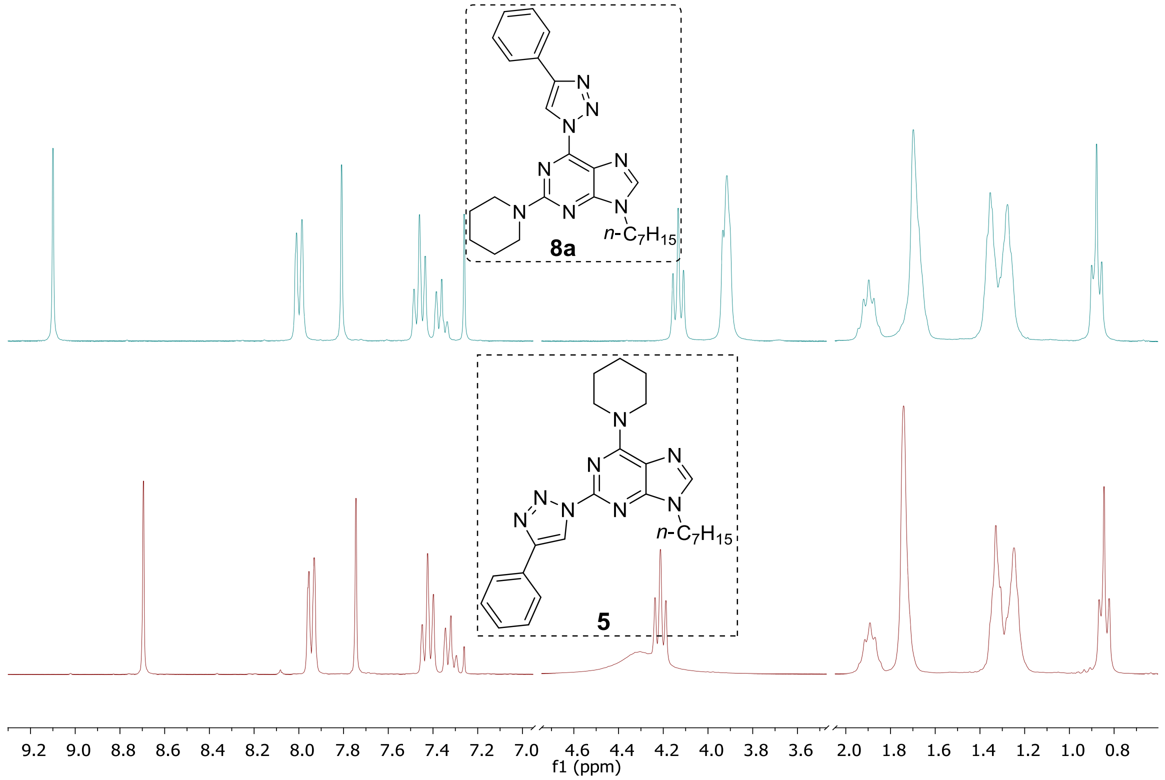

Figure S2: Comparison of ^{1}H NMR spectra of compounds **8a** and **5** (300 MHz, $CDCl_3$), full spectra.

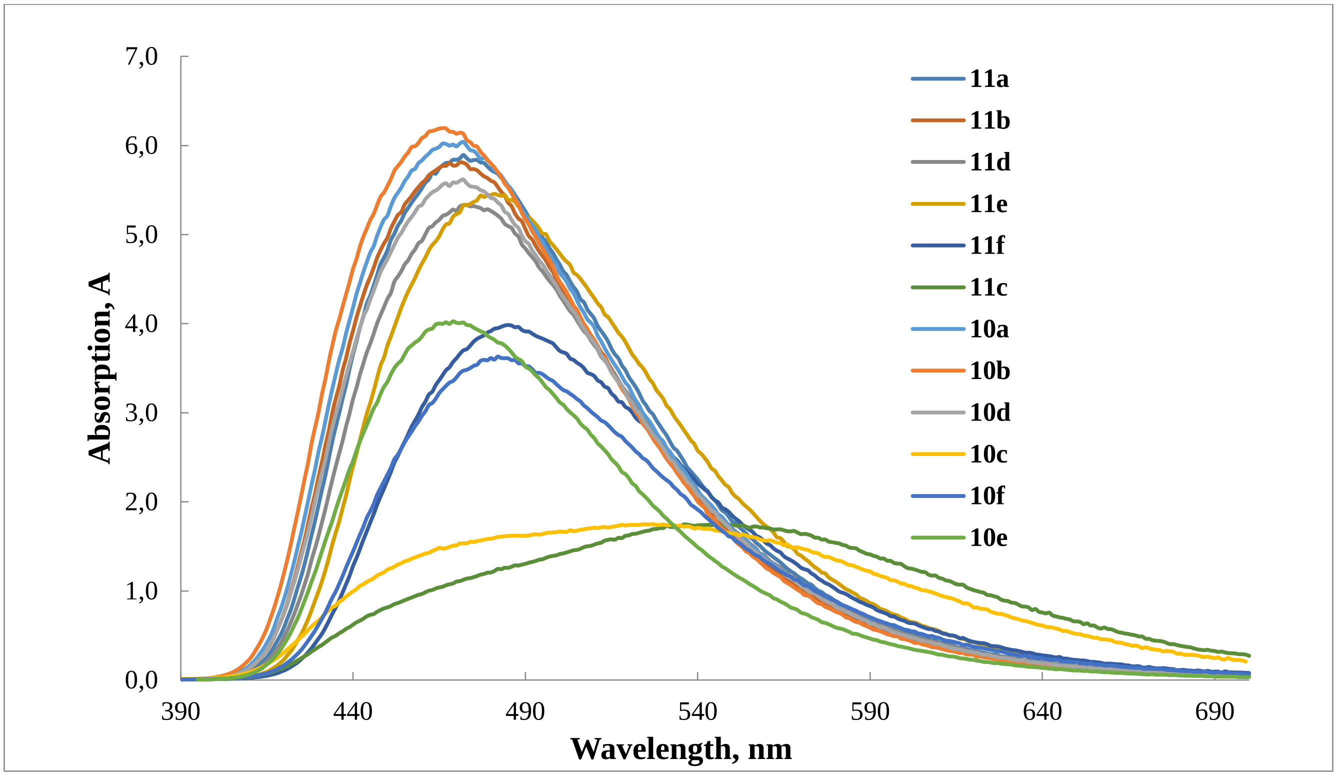

Figure S3: Emission spectra for 7-deazacompouds (c = 10^{-4} M, c = 0.5 × 10^{-4} M for compound **10c**; solvent – MeCN).

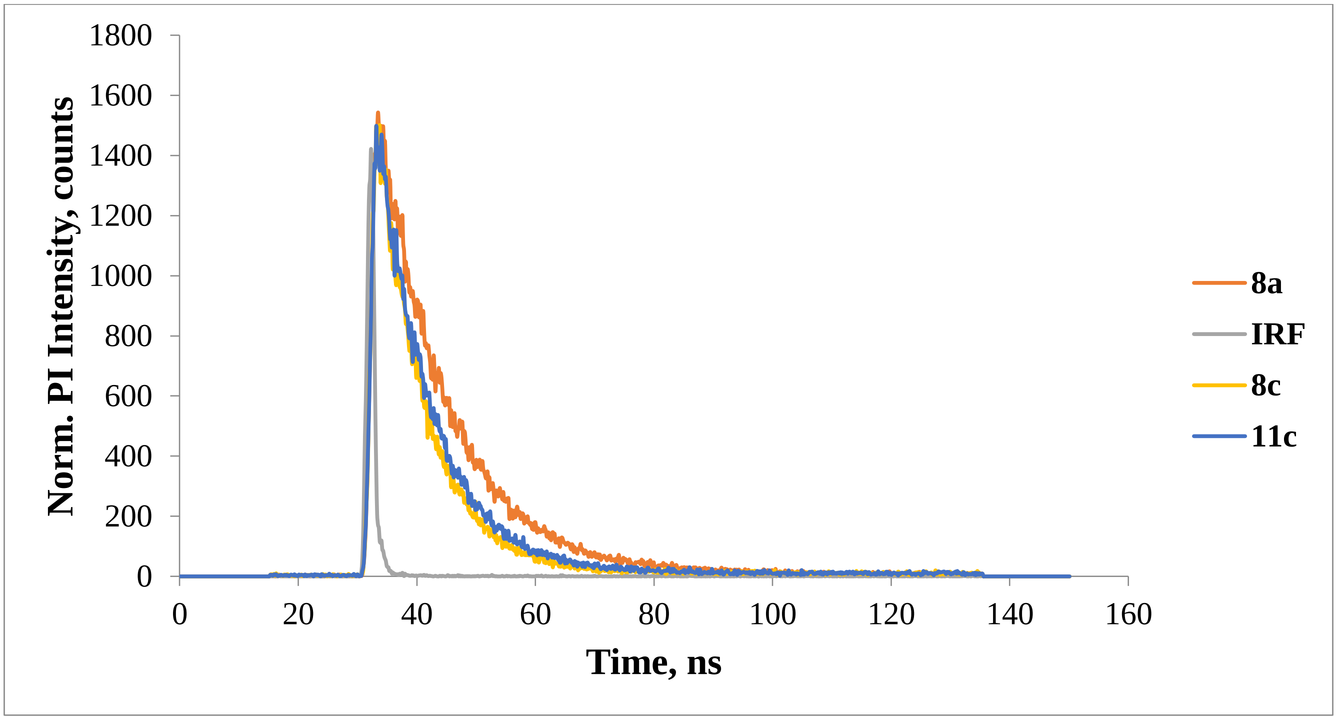

Figure S4: Measurements of fluorescence decay times of compounds **8a**, **8c** and **11c** in MeCN (c = 10^{-4} M).

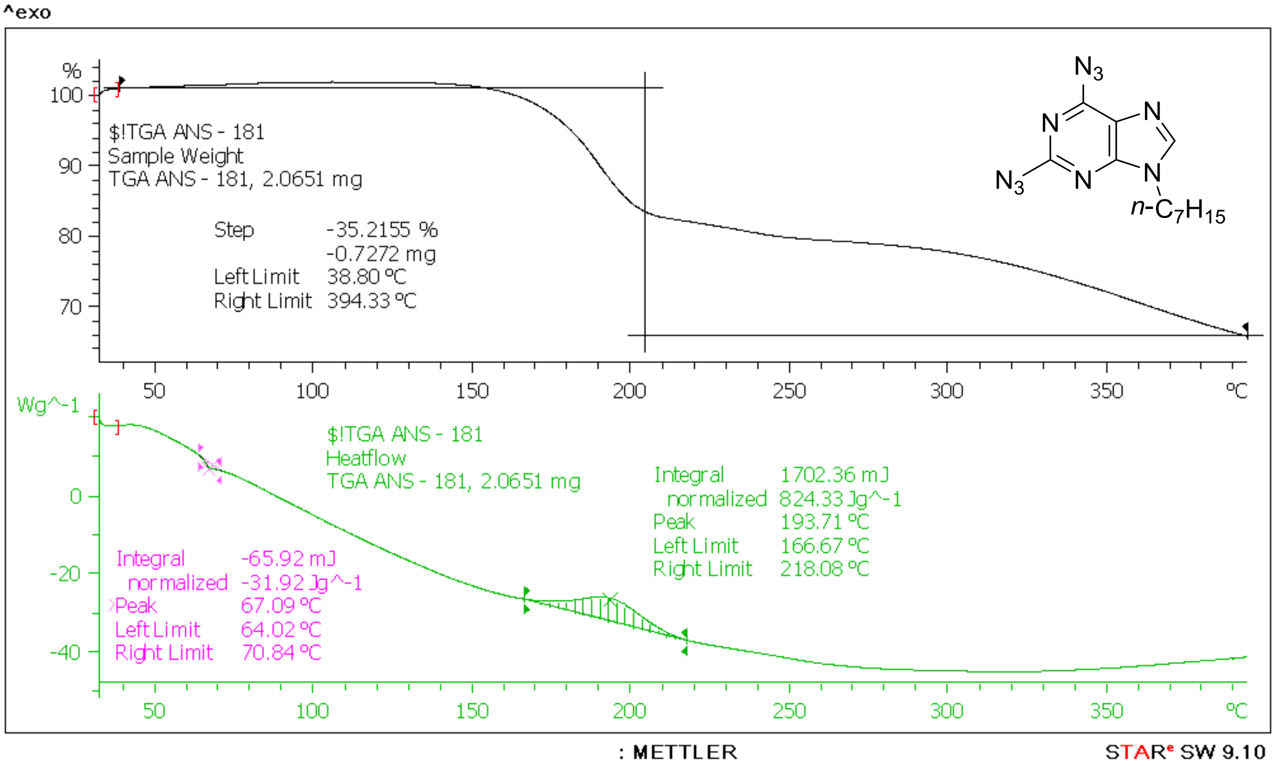

Figure S5: DSC data for compound **2a**.

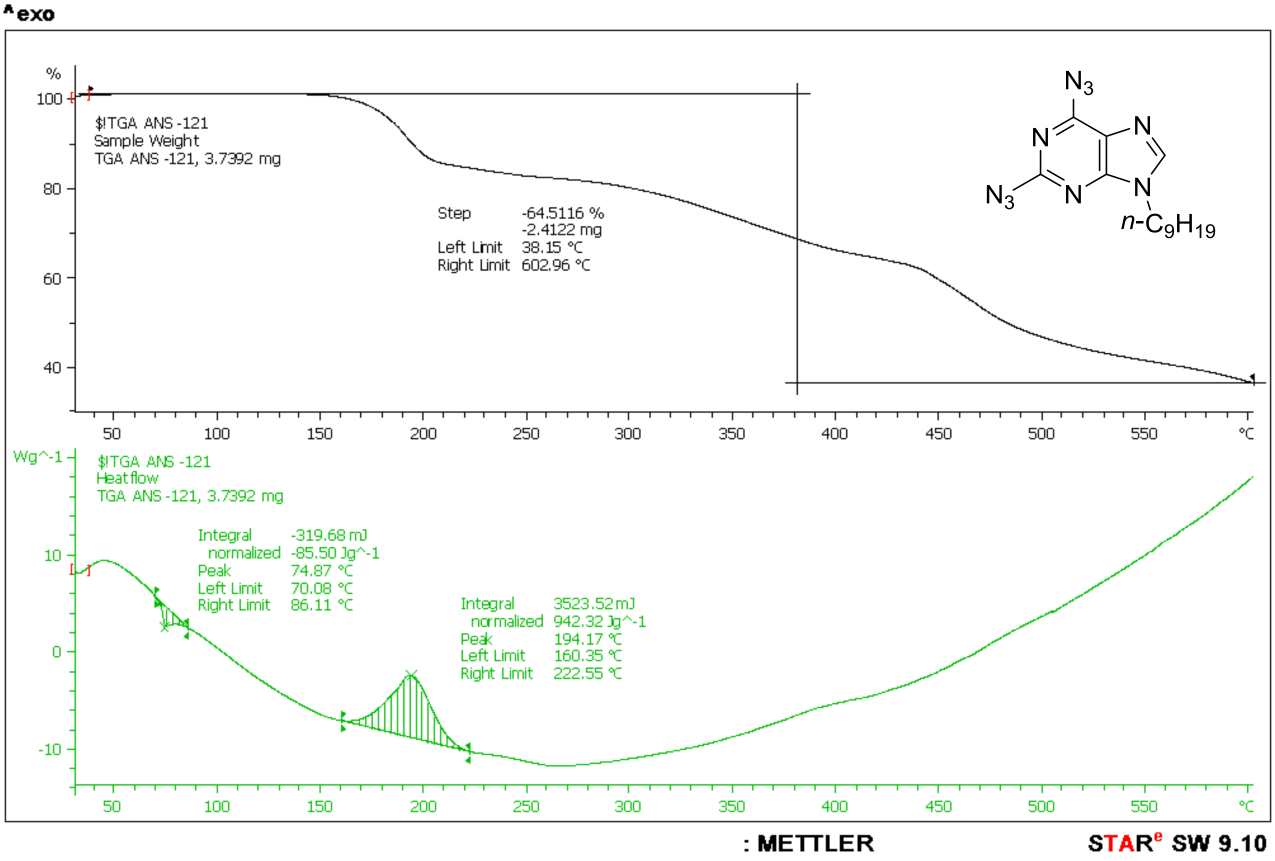

Figure S6: DSC data for compound **2b**.

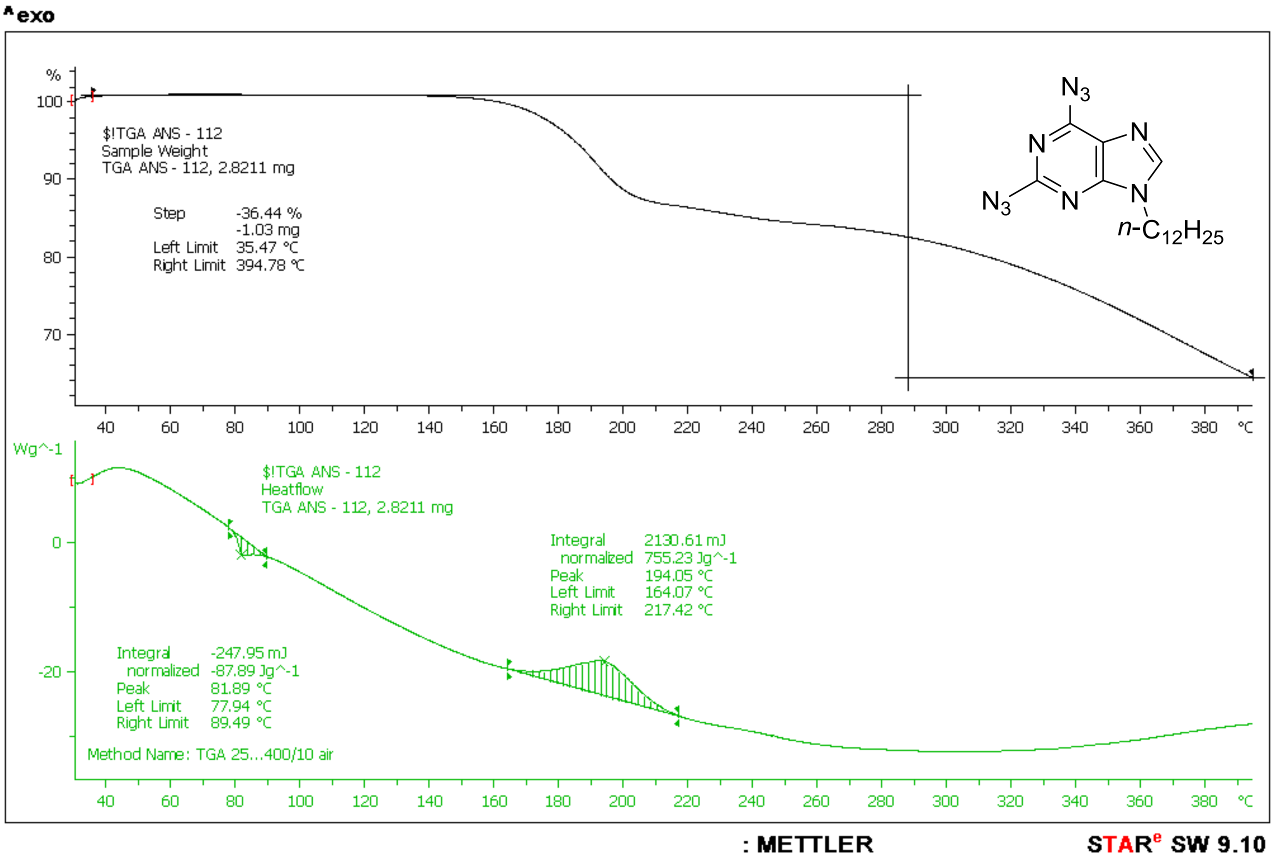

Figure S7: DSC data for compound **2c**.

Copies of ^{1}H and ^{13}C NMR spectra

2,6-Dichloro-9-heptyl-9*H*-purine (1a-1)

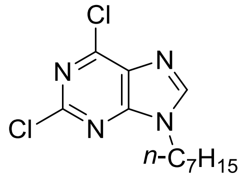

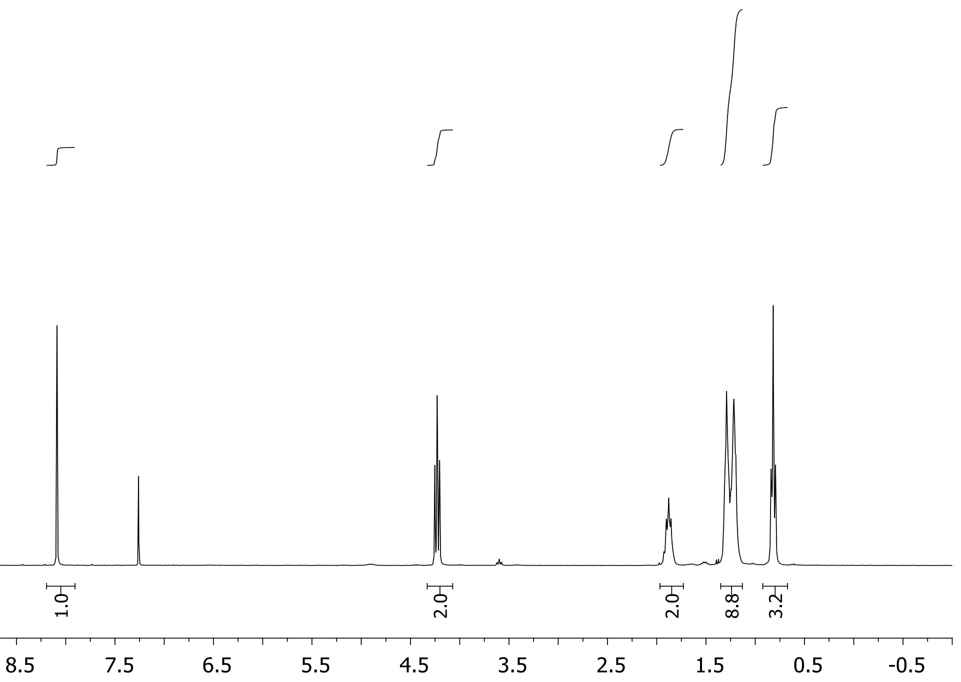

Figure S8: ^{1}H NMR (300 MHz, $CDCl_3$) spectrum.

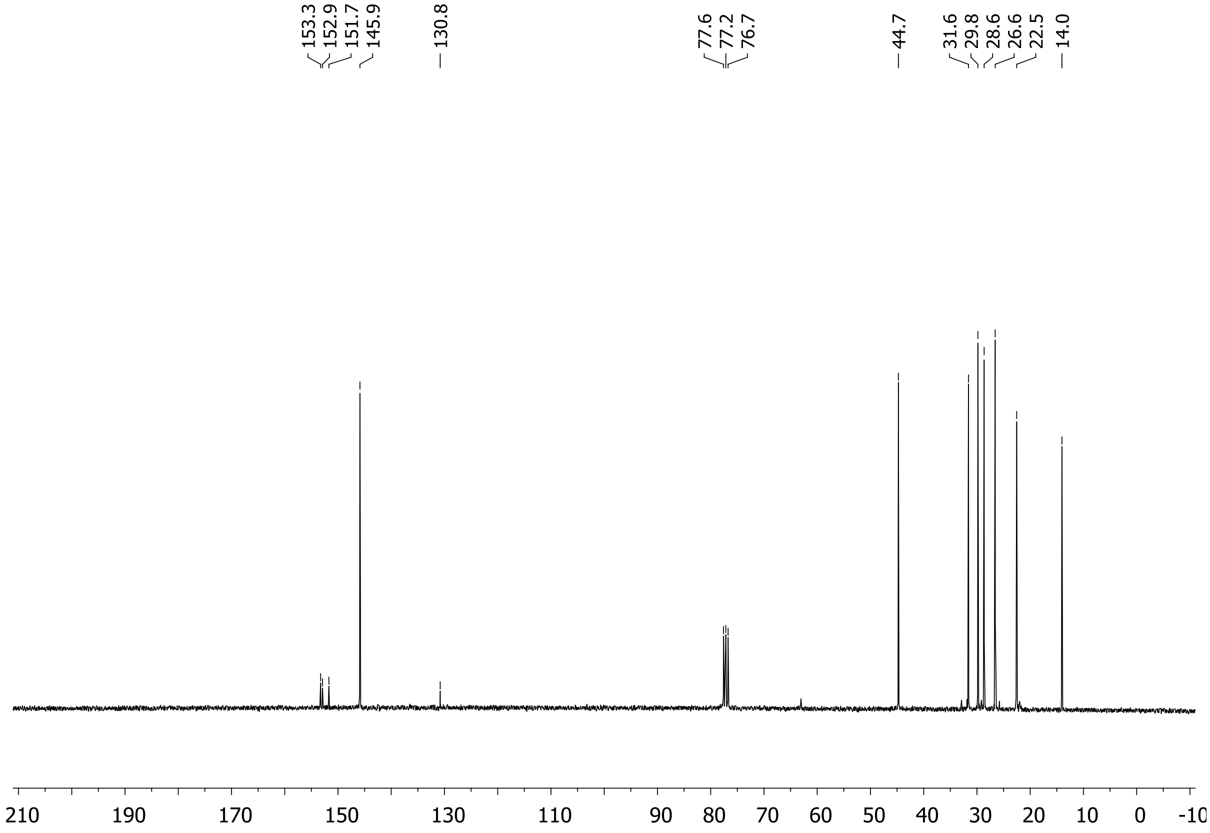

Figure S9: ^{13}C NMR (75.5 MHz, $CDCl_3$) spectrum.

2,6-Dichloro-9-nonyl-9*H*-purine (1a-2)

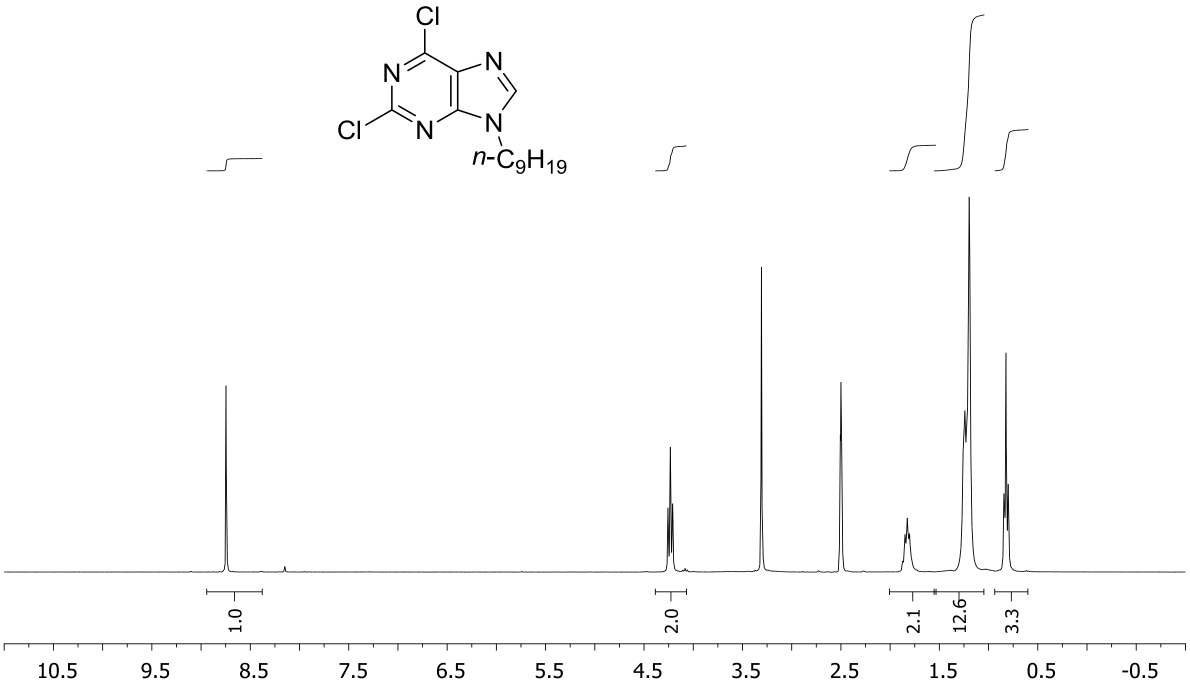

Figure S10: ^{1}H NMR (300 MHz, DMSO-d_6) spectrum.

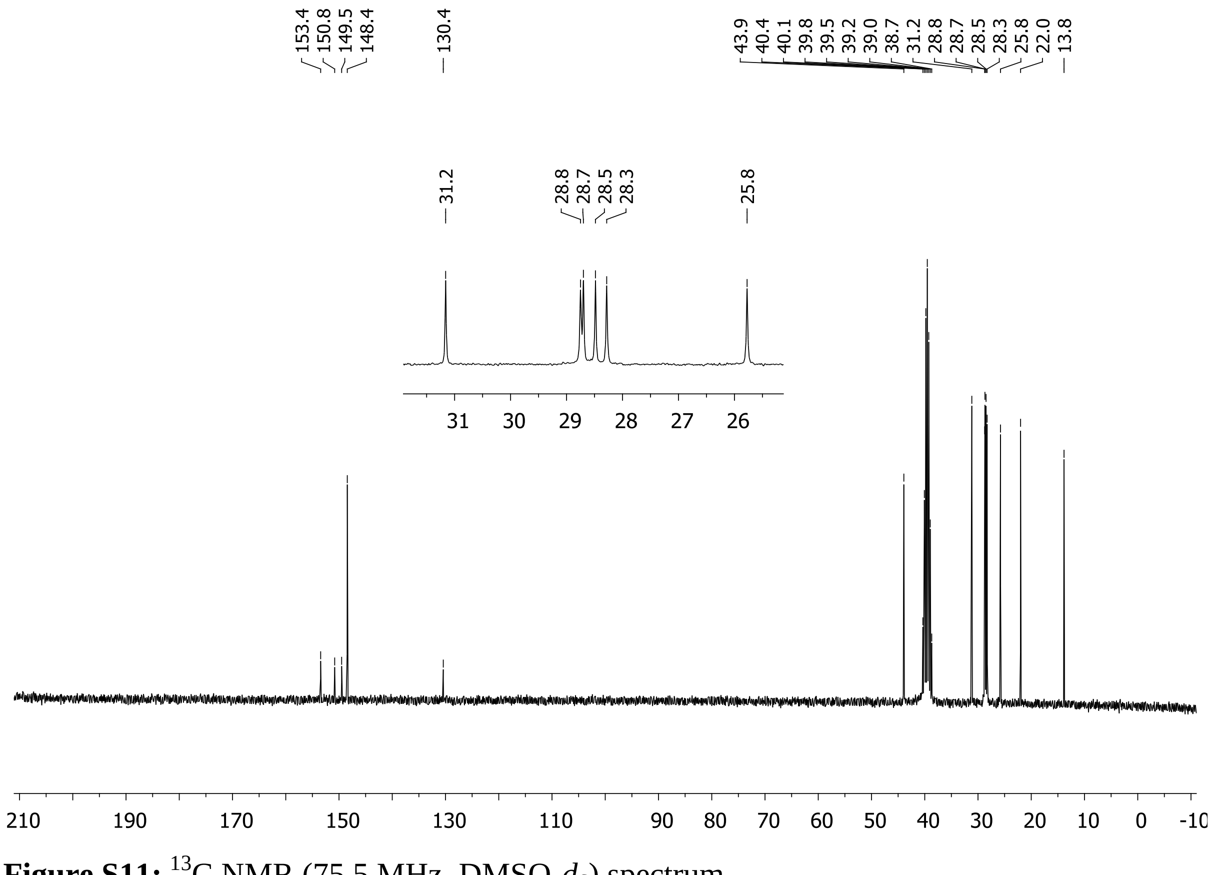

Figure S11: ^{13}C NMR (75.5 MHz, DMSO-d_6) spectrum.

2,6-Dichloro-9-dodecyl-9*H*-purine (1a-3)

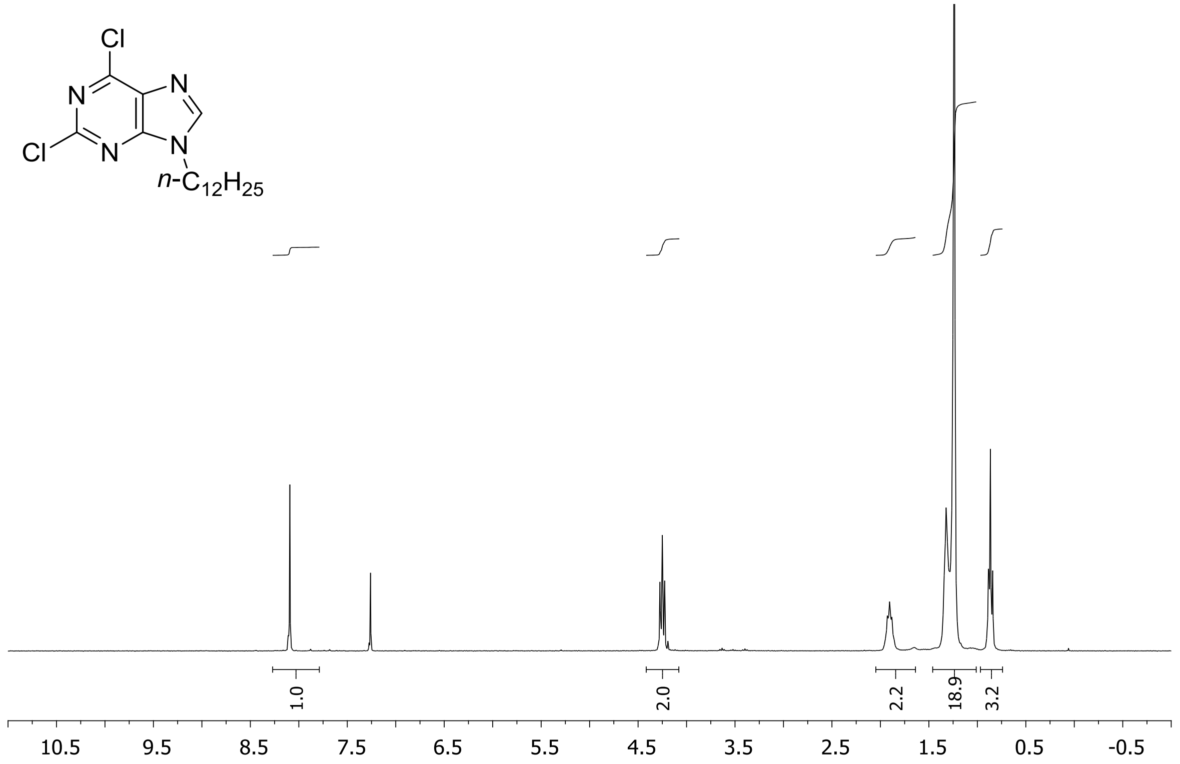

Figure S12: ^{1}H NMR (300 MHz, $CDCl_3$) spectrum.

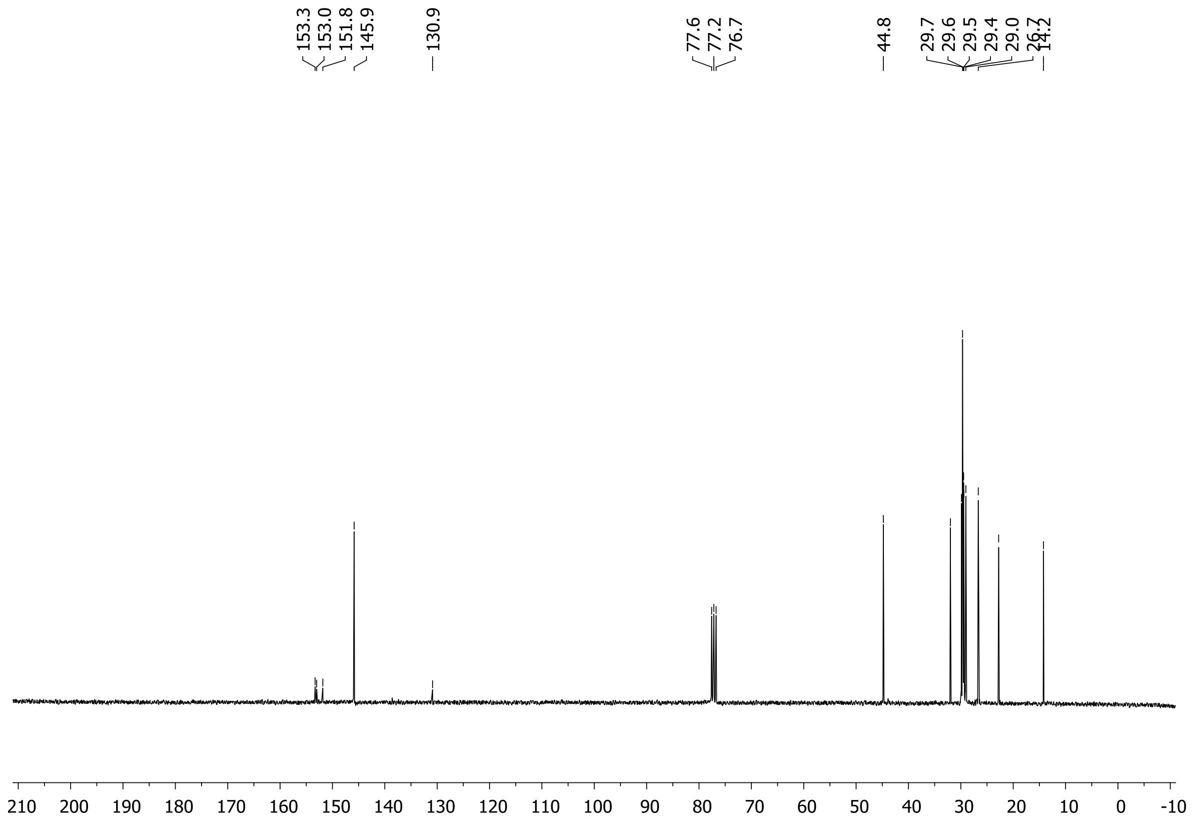

Figure S13: ^{13}C NMR (75.5 MHz, $CDCl_3$) spectrum.

2,6-Diazido-9-heptyl-9*H*-purine (2a)

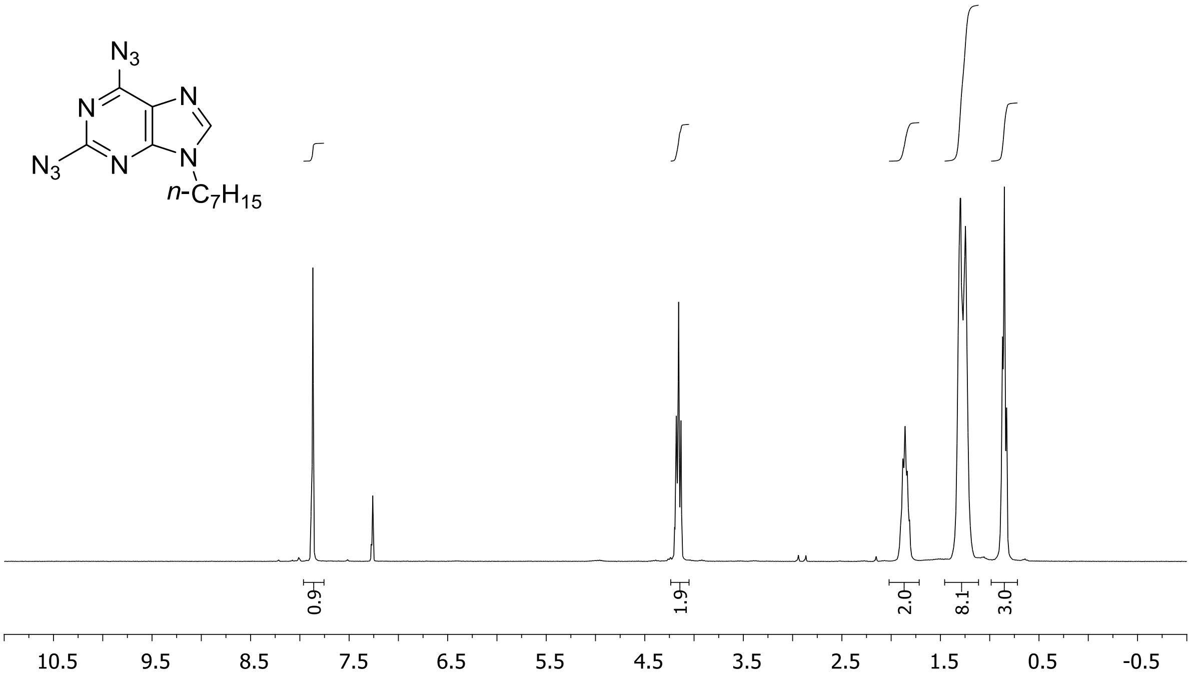

Figure S14: ^{1}H NMR (300 MHz, $CDCl_3$) spectrum.

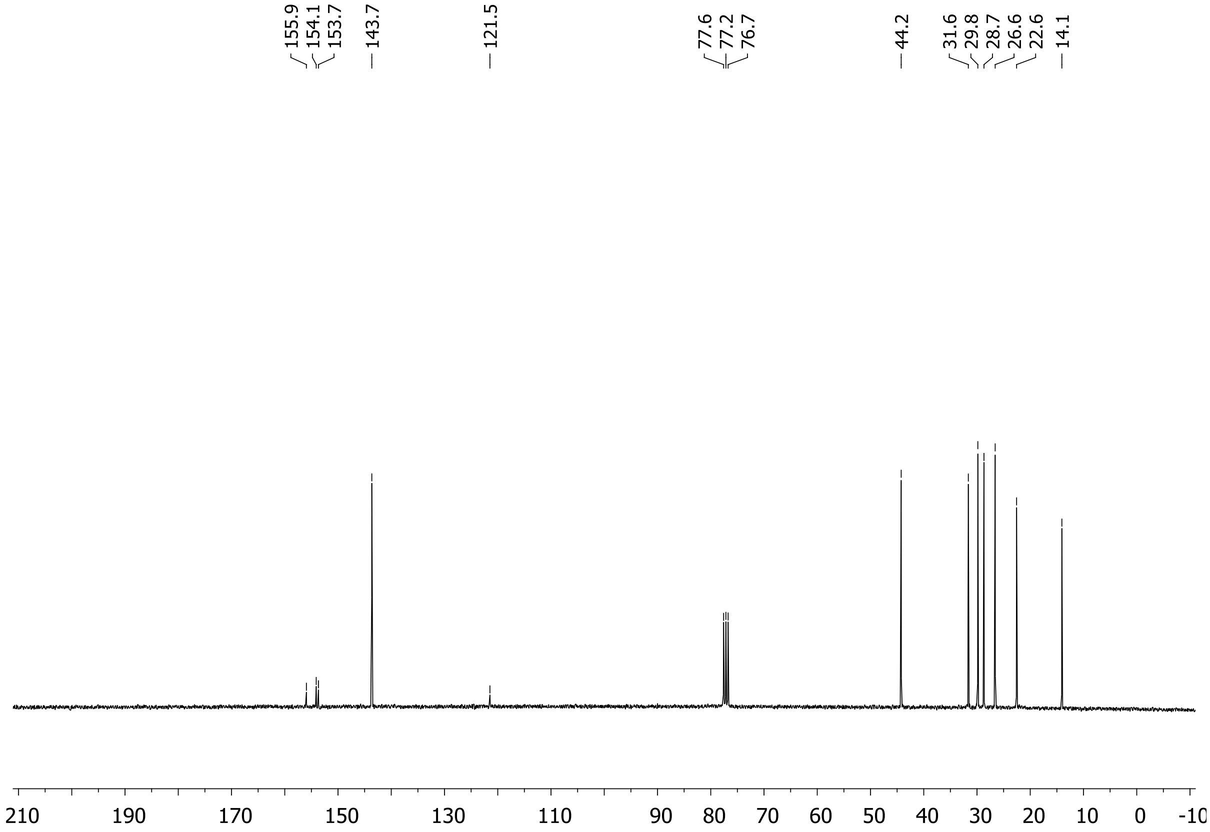

Figure S15: ^{13}C NMR (75.5 MHz, $CDCl_3$) spectrum.

2,6-Diazido-9-nonyl-9H-purine (2b)

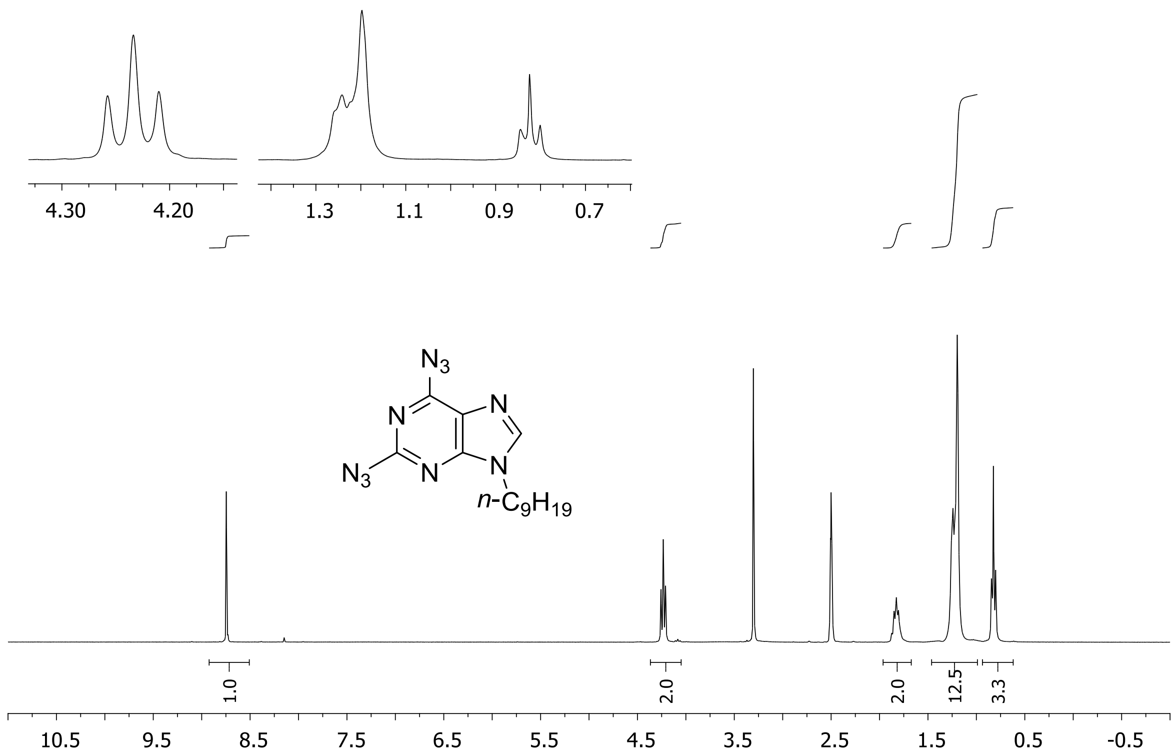

Figure S16: ^{1}H NMR (300 MHz, DMSO-d_6) spectrum.

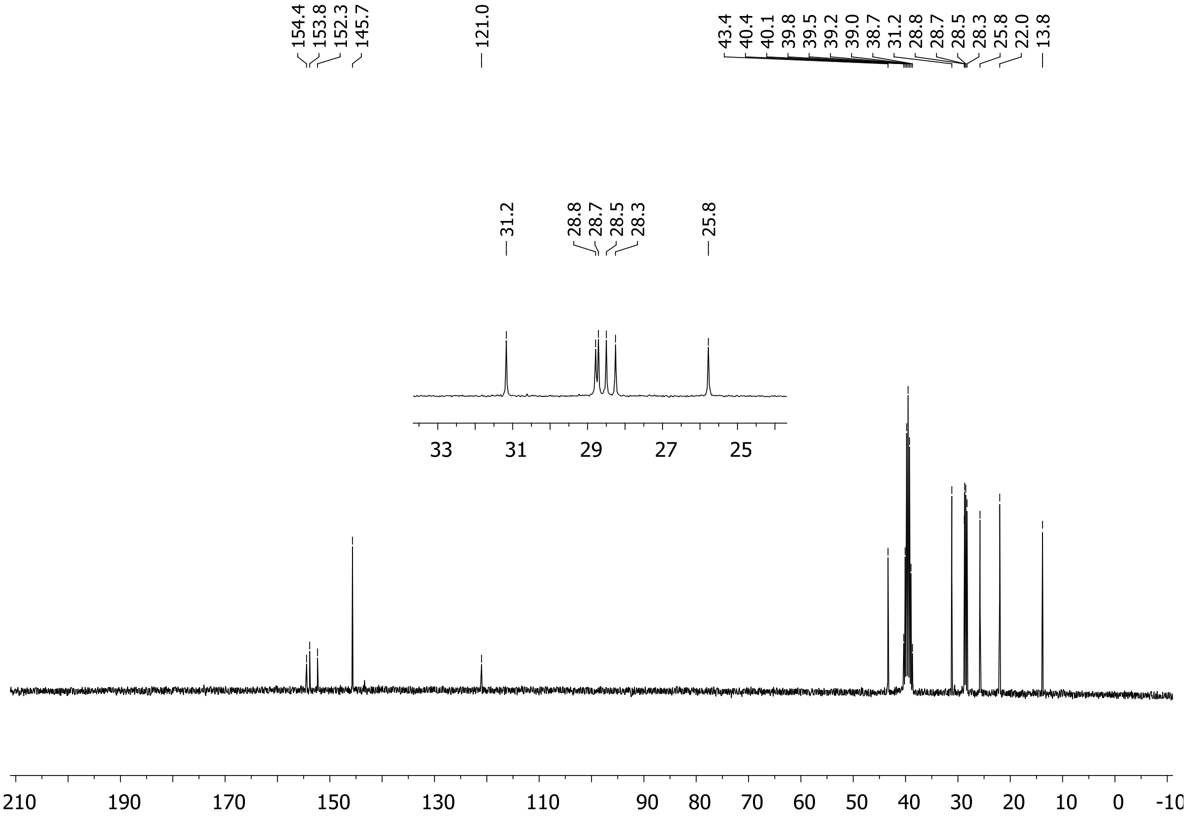

Figure S17: ^{13}C NMR (75.5 MHz, DMSO-d_6) spectrum.

2,6-Diazido-9-dodecyl-9*H*-purine (2c)

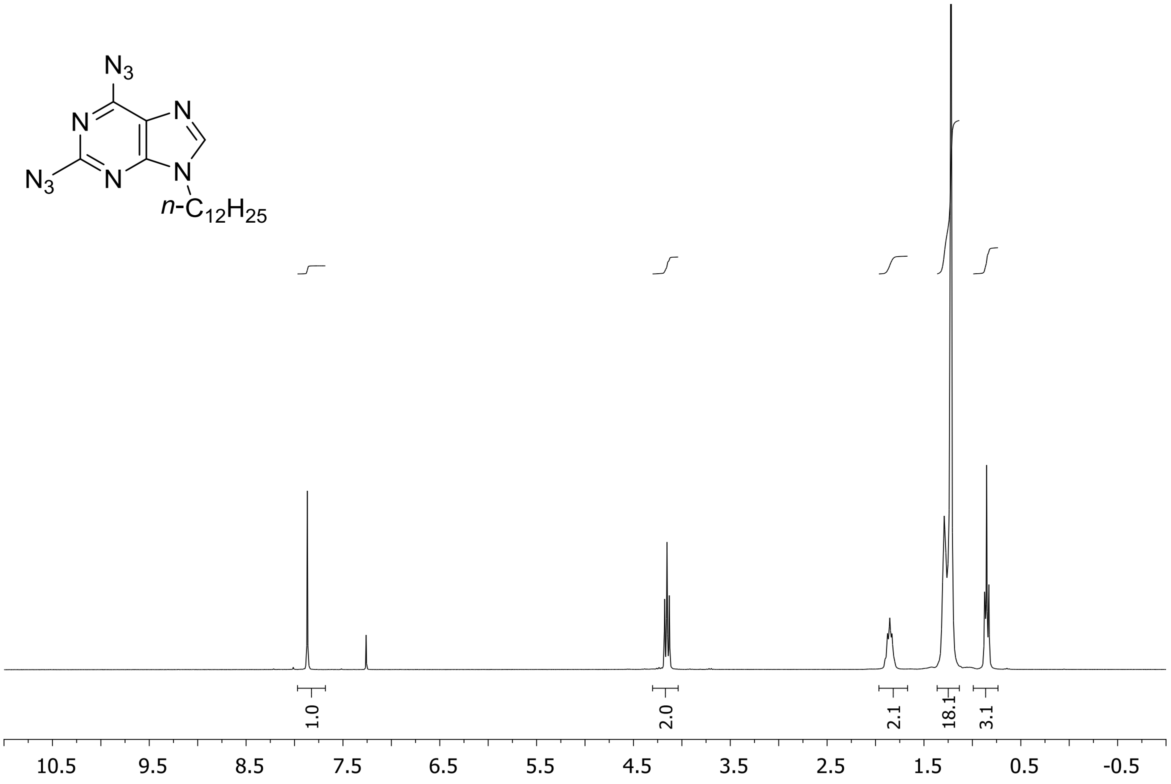

Figure S18: ^{1}H NMR (300 MHz, $CDCl_3$) spectrum.

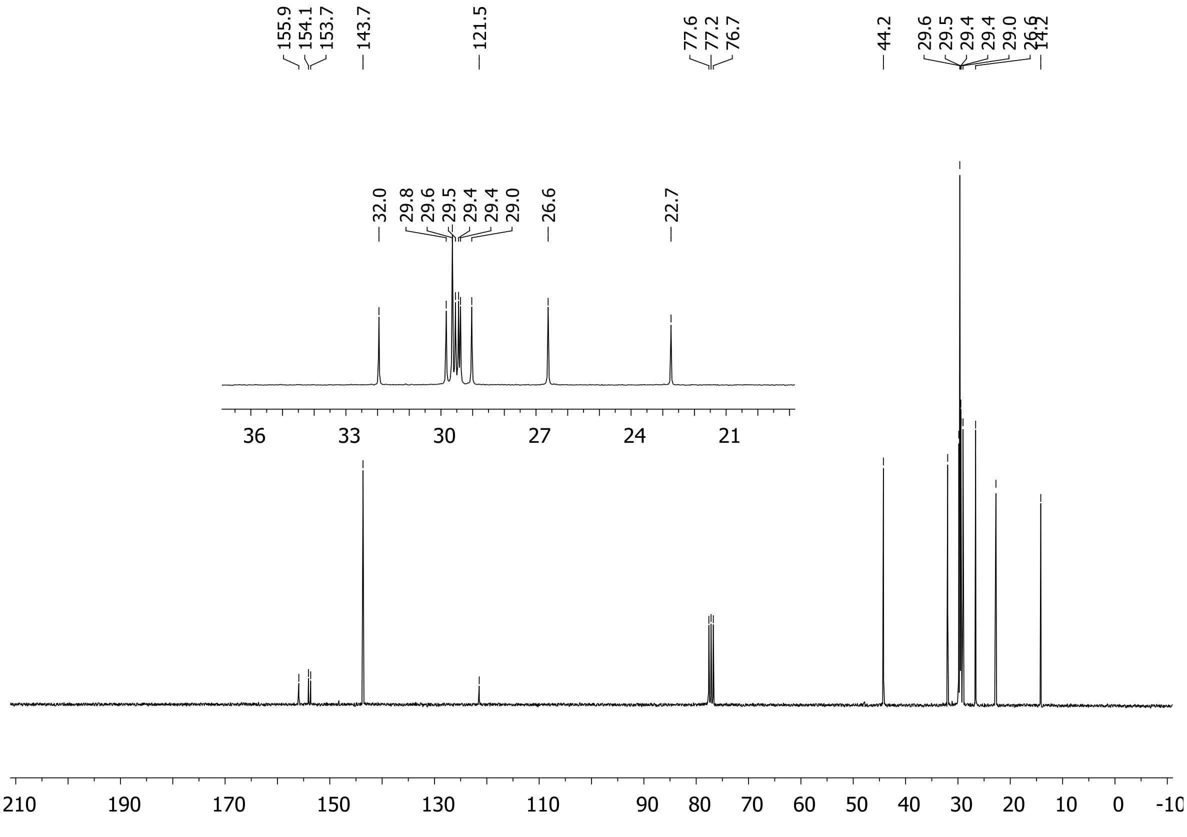

Figure S19: ^{13}C NMR (75.5 MHz, $CDCl_3$) spectrum.

2,6-Bis(4-phenyl-1*H*-1,2,3-triazol-1-yl)-9-heptyl-9*H*-purine (4)

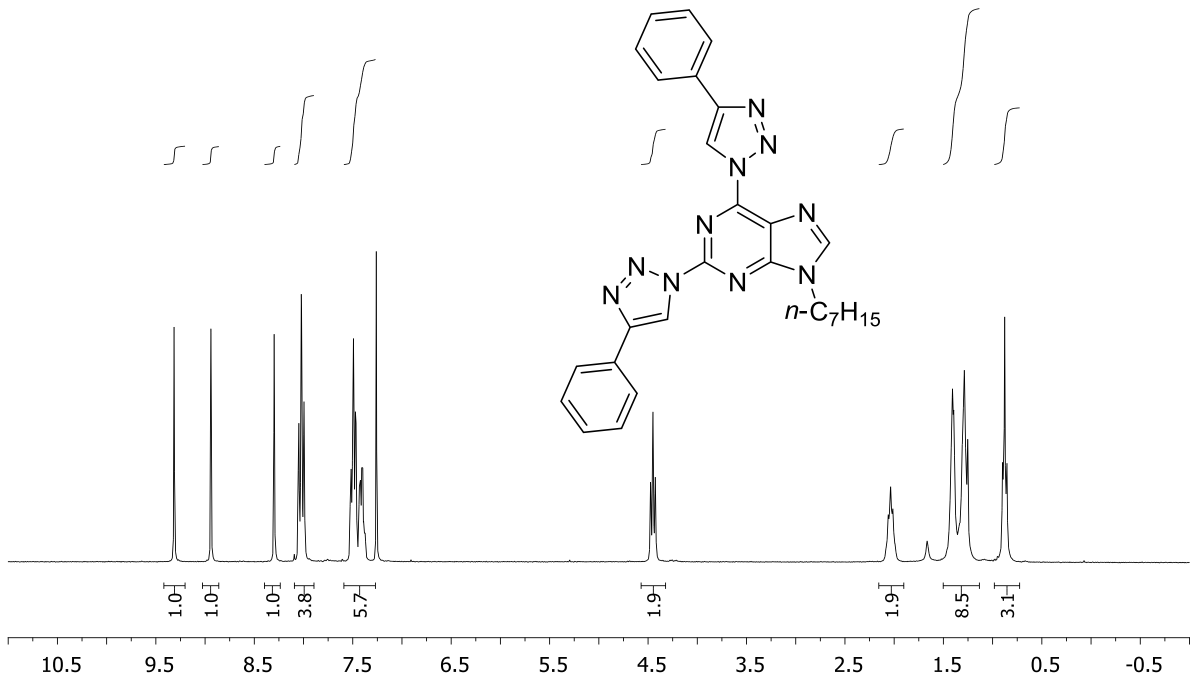

Figure S20: ^{1}H NMR (300 MHz, $CDCl_3$) spectrum.

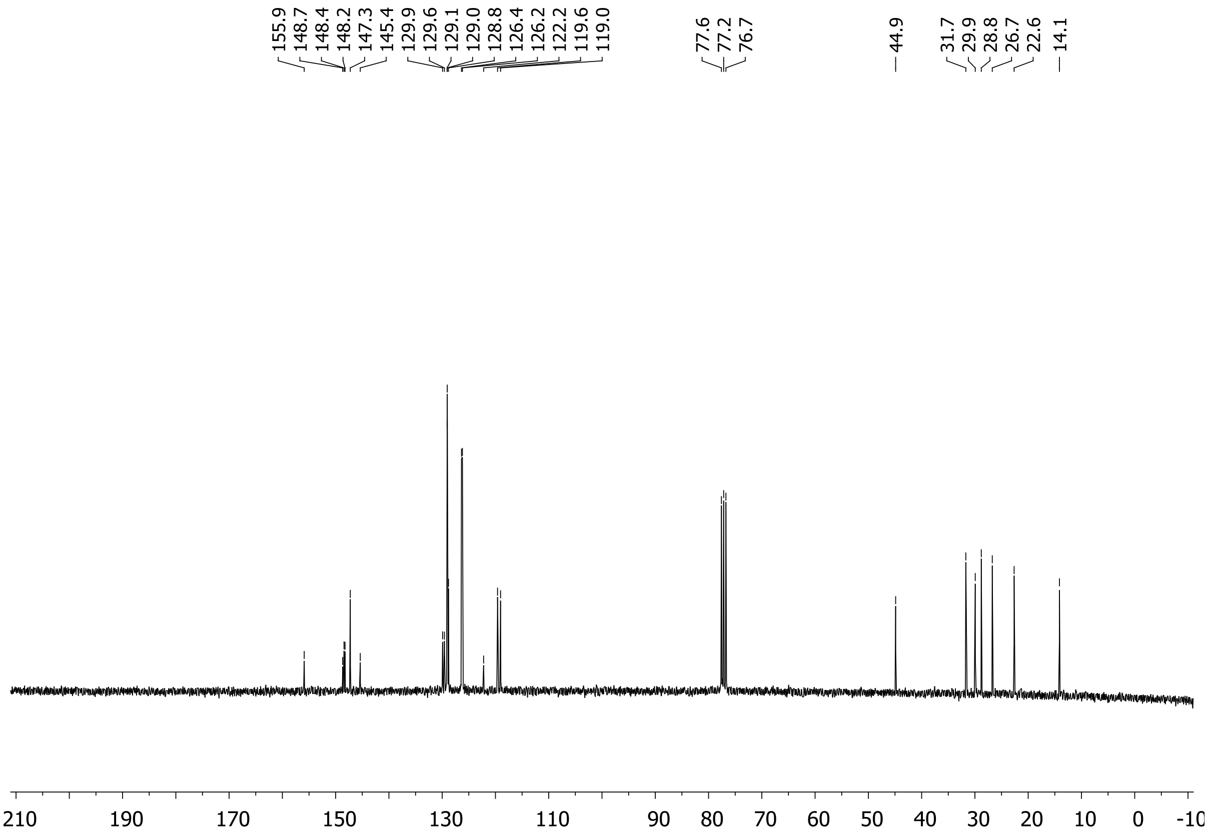

Figure S21: ^{13}C NMR (75.5 MHz, $CDCl_3$) spectrum.

9-Heptyl-2-(4-phenyl-1*H*-1,2,3-triazol-1-yl)-6-piperidino-9*H*-purine (5)

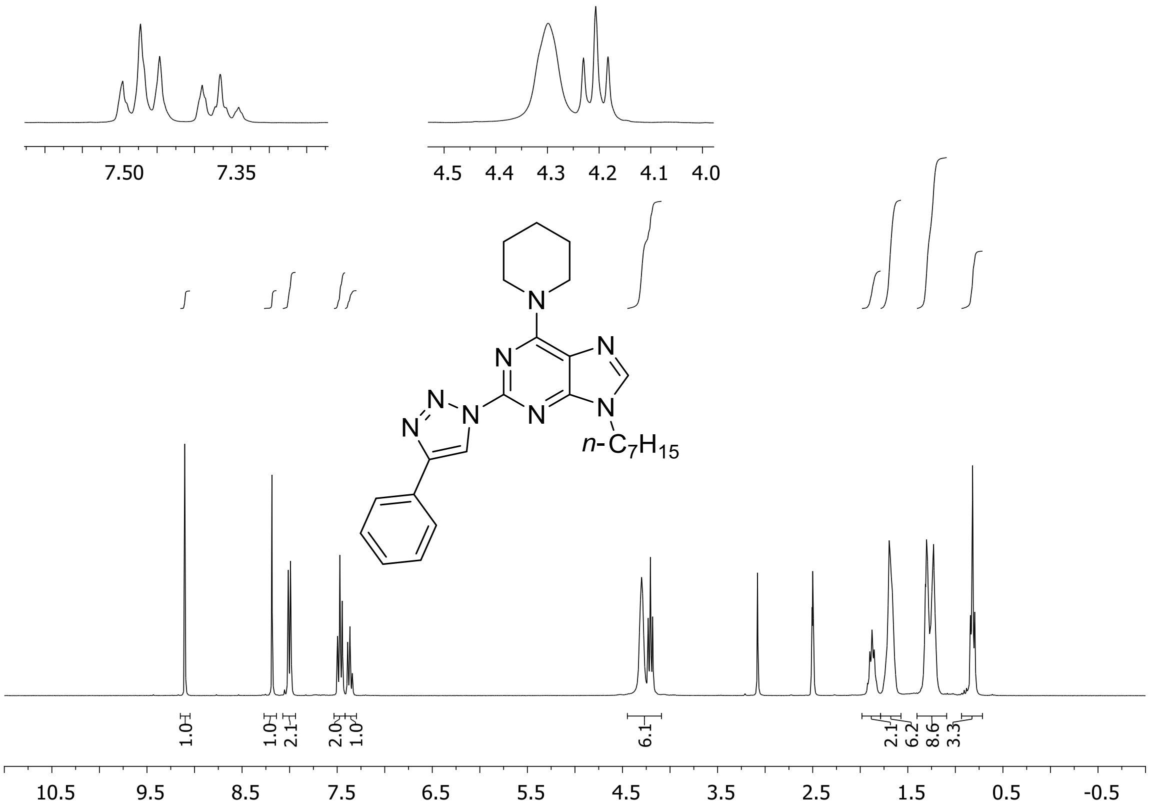

Figure S22: ^{1}H NMR (300 MHz, 70 °C, DMSO-d_6) spectrum.

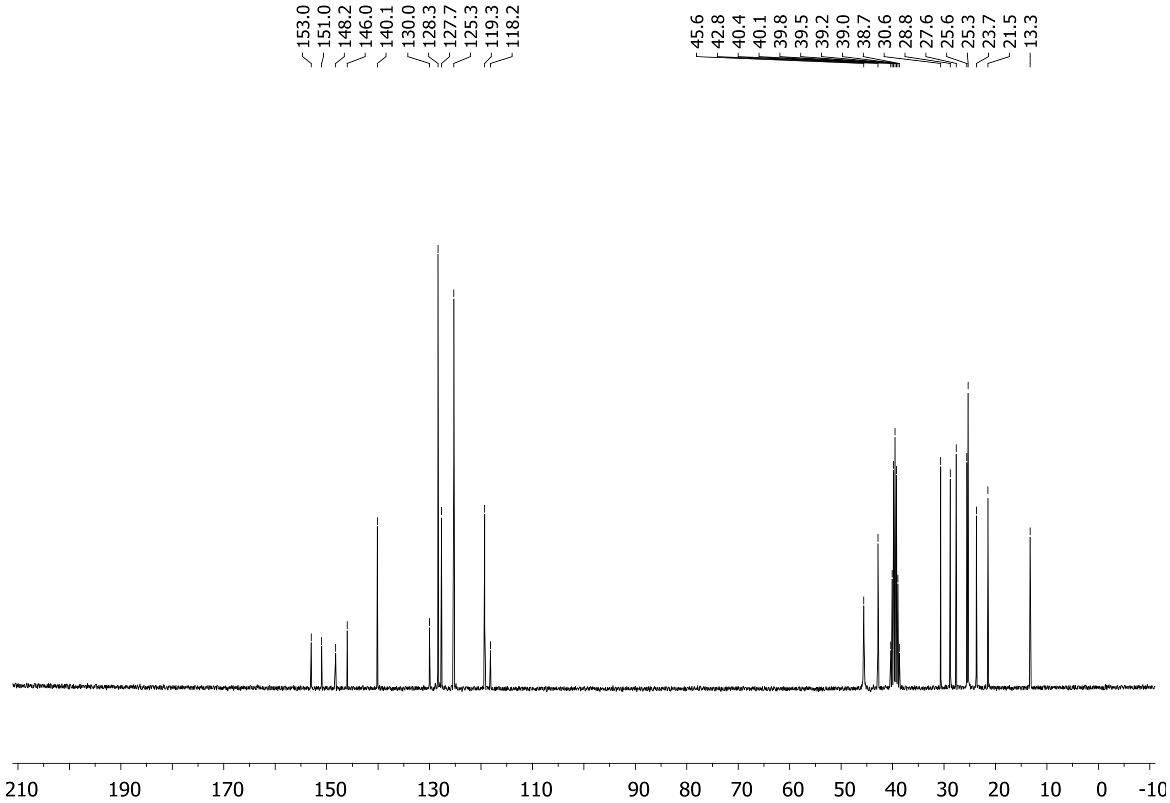

Figure S23: ^{13}C NMR (75.5 MHz, 70 °C, DMSO-d_6) spectrum.

6-Azido-9-heptyl-2-pyrrolidino-9H-purine (6a)

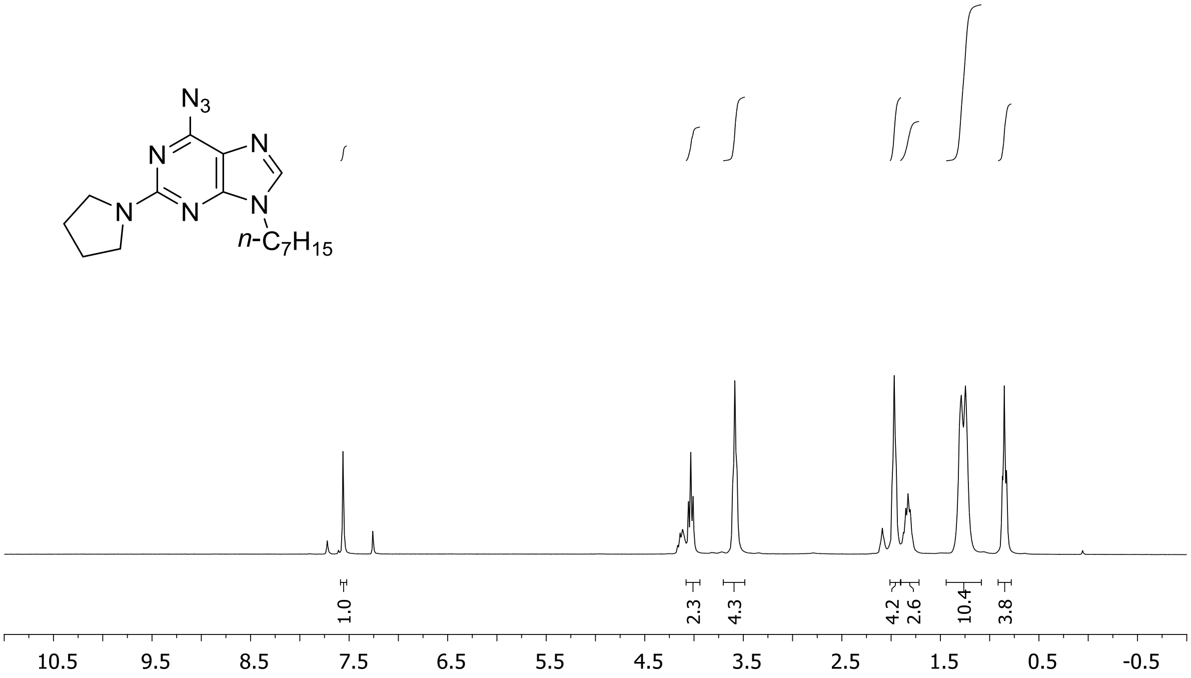

Figure S24: ^{1}H NMR (300 MHz, $CDCl_3$) spectrum.

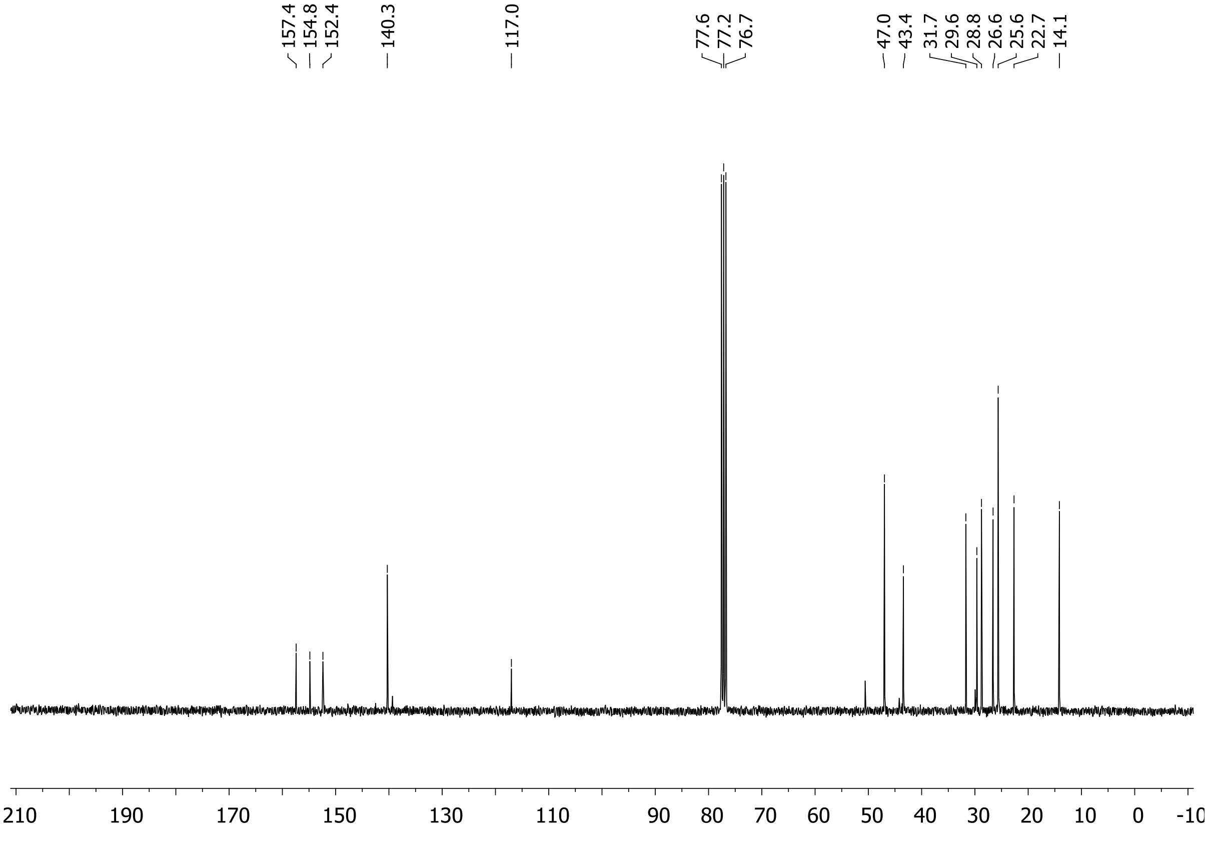

Figure S25: ^{13}C NMR (75.5 MHz, $CDCl_3$) spectrum.

6-Azido-9-heptyl-2-piperidino-9*H*-purine (6b)

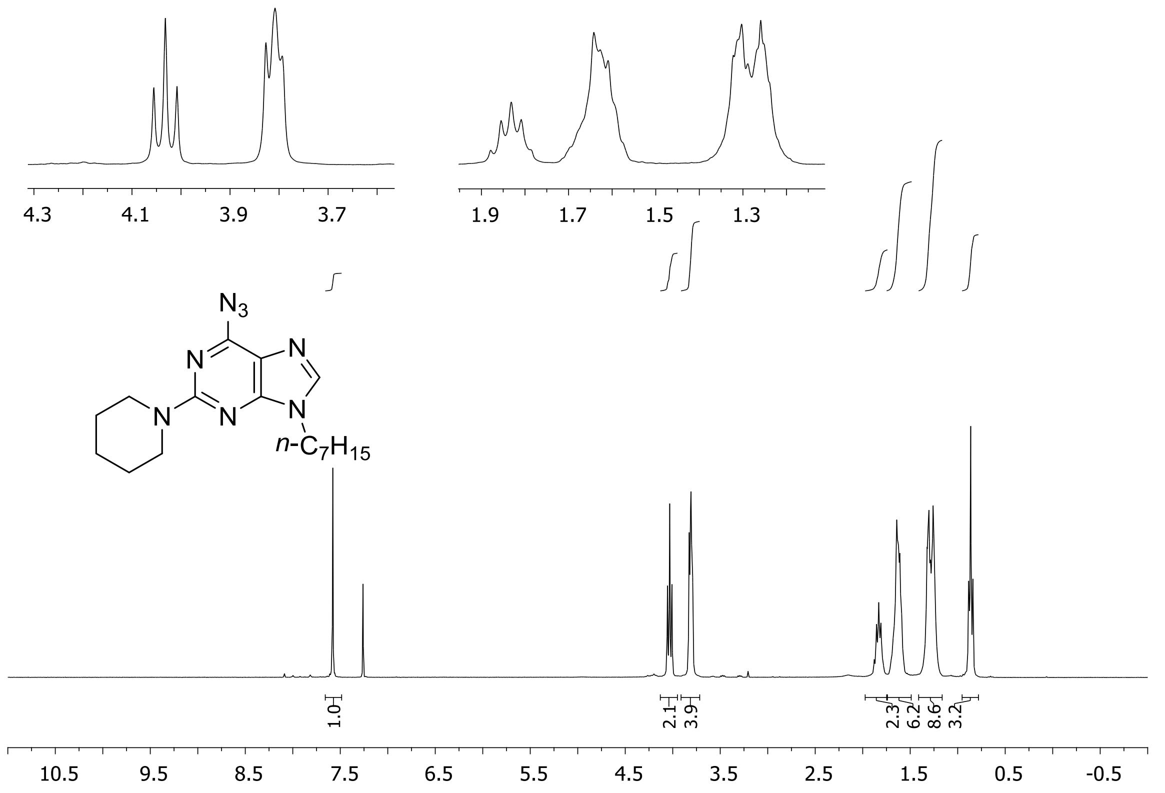

Figure S26: ^{1}H NMR (300 MHz, $CDCl_3$) spectrum.

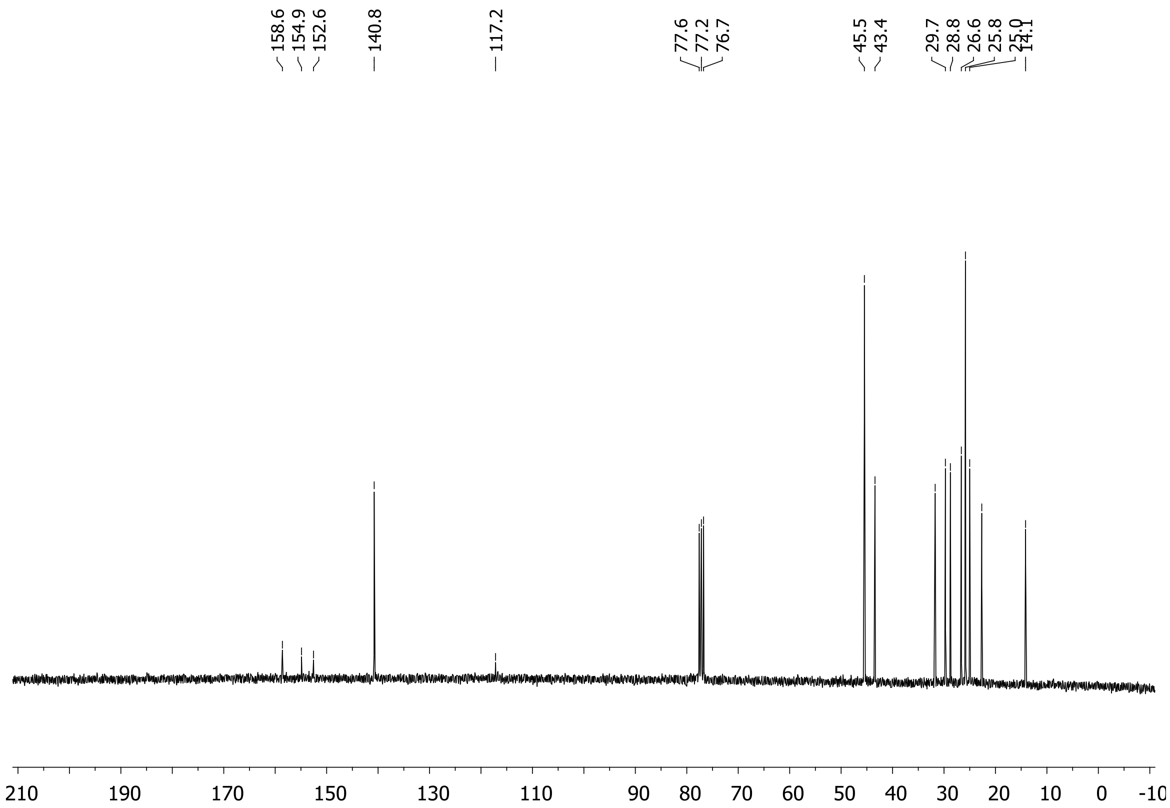

Figure S27: ^{13}C NMR (75.5 MHz, $CDCl_3$) spectrum.

9-Heptyl-6-(4-phenyl-1*H*-1,2,3-triazol-1-yl)-2-pyrrolidino-9*H*-purine (7a)

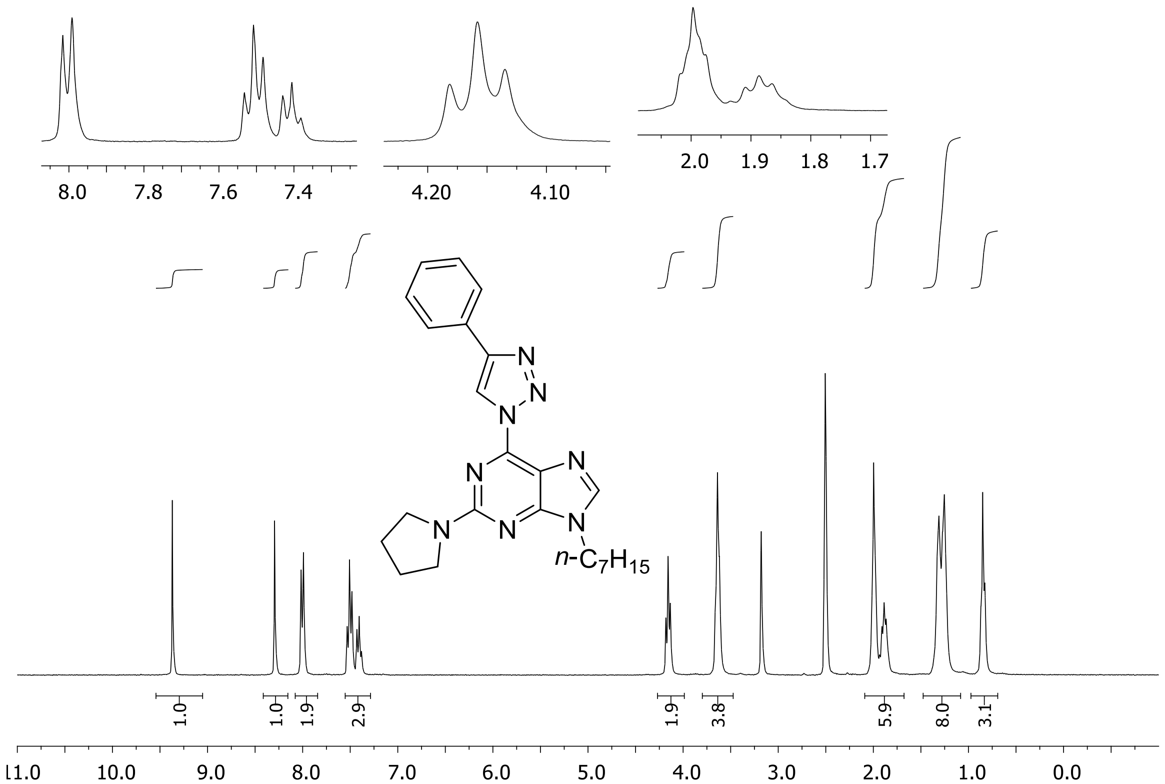

Figure S28: ^{1}H NMR (300 MHz, 70 °C, DMSO-d_6 + D_2O) spectrum.

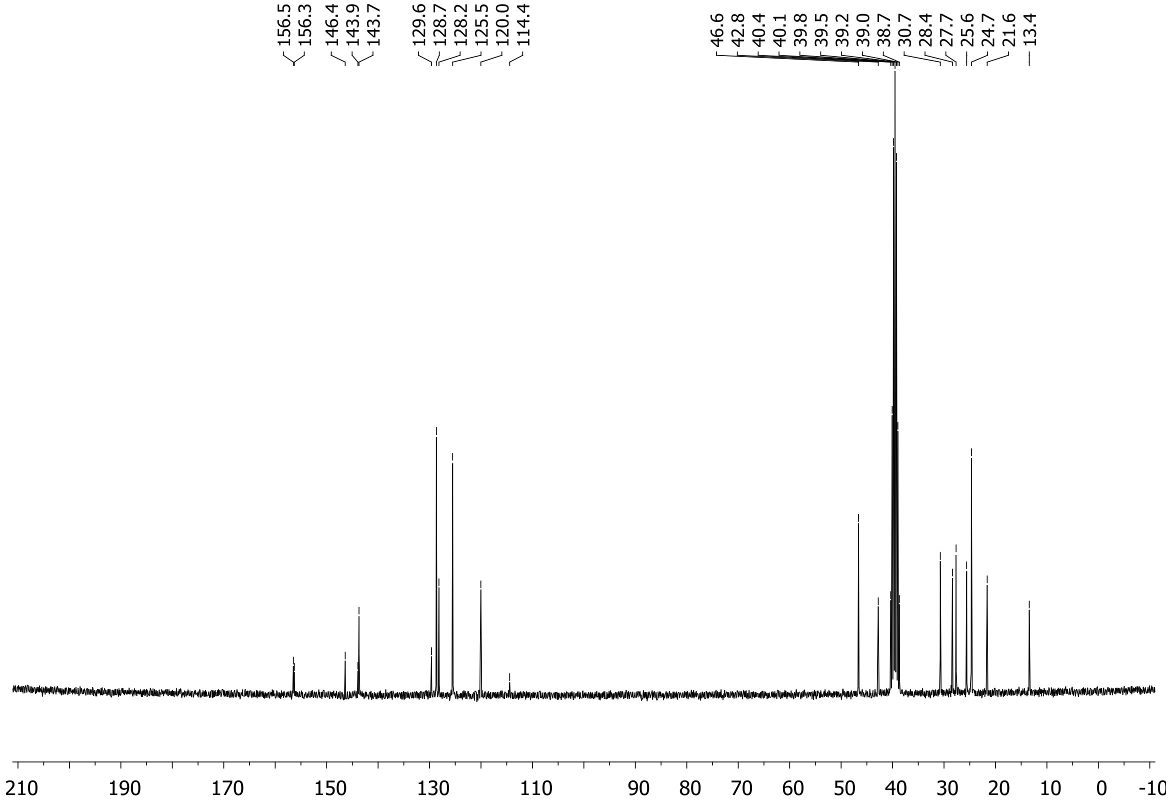

Figure S29: ^{13}C NMR (75.5 MHz, 70 °C, DMSO-d_6 + D_2O) spectrum.

9-Heptyl-6-(4-(4-metoxyphenyl)-1*H*-1,2,3-triazol-1-yl)-2-pyrrolidino-9*H*-purine (7b)

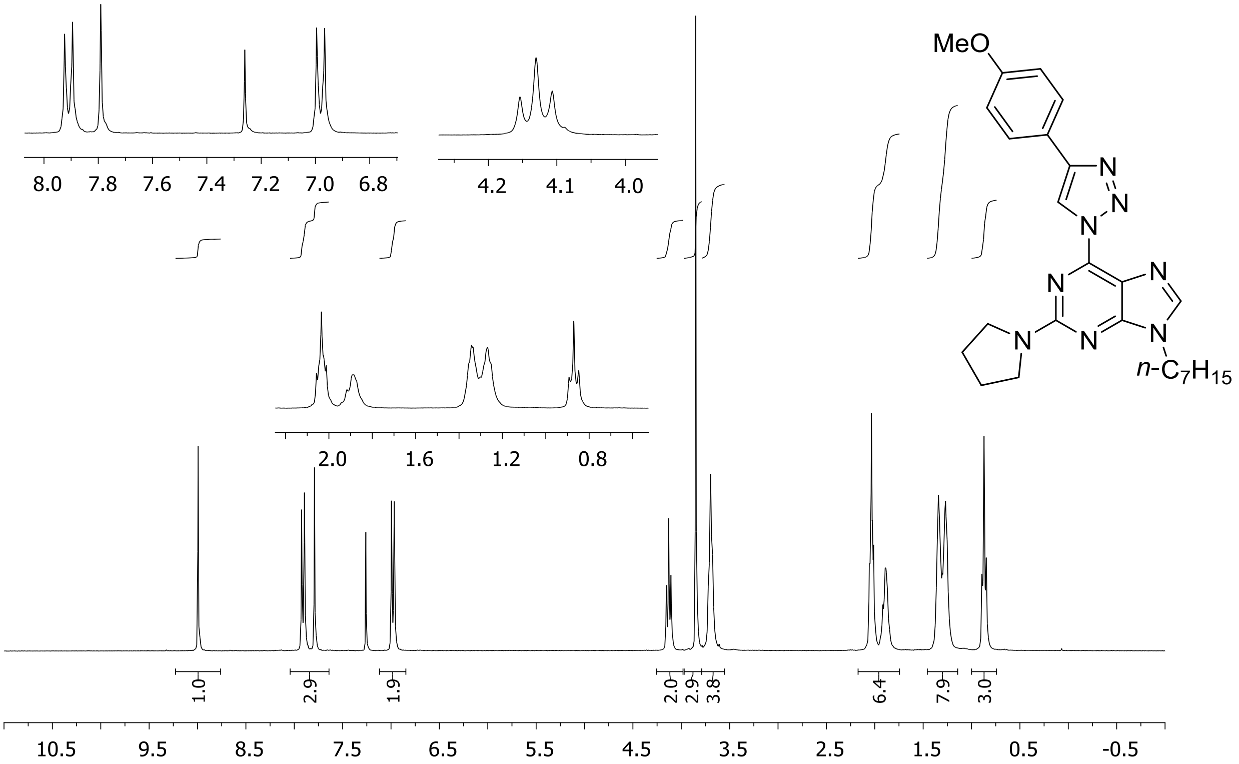

Figure S30: ^{1}H NMR (300 MHz, $CDCl_3$) spectrum.

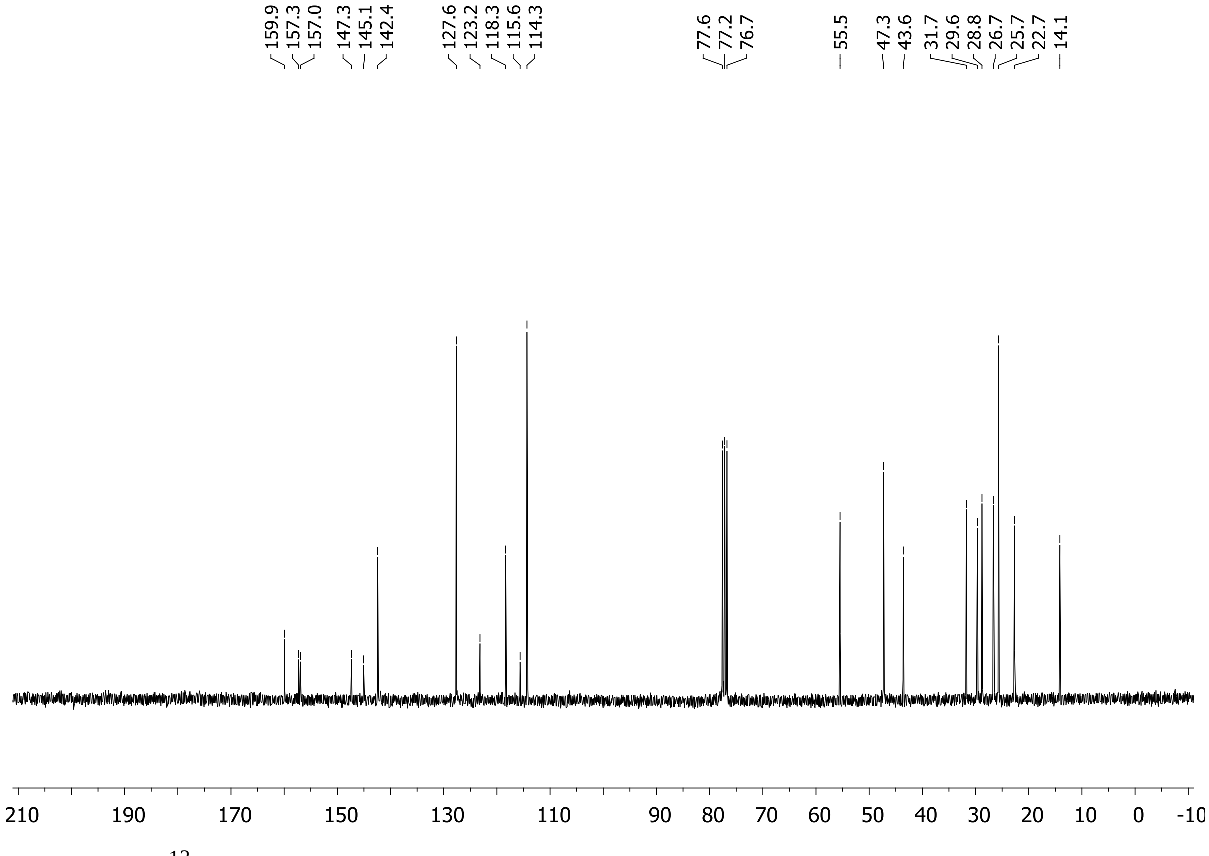

Figure S31: ^{13}C NMR (75.5 MHz, $CDCl_3$) spectrum.

9-Heptyl-6-(4-(4-dimethylaminophenyl)-1*H*-1,2,3-triazol-1-yl)-2-pyrrolidino-9*H*-purine (7c)

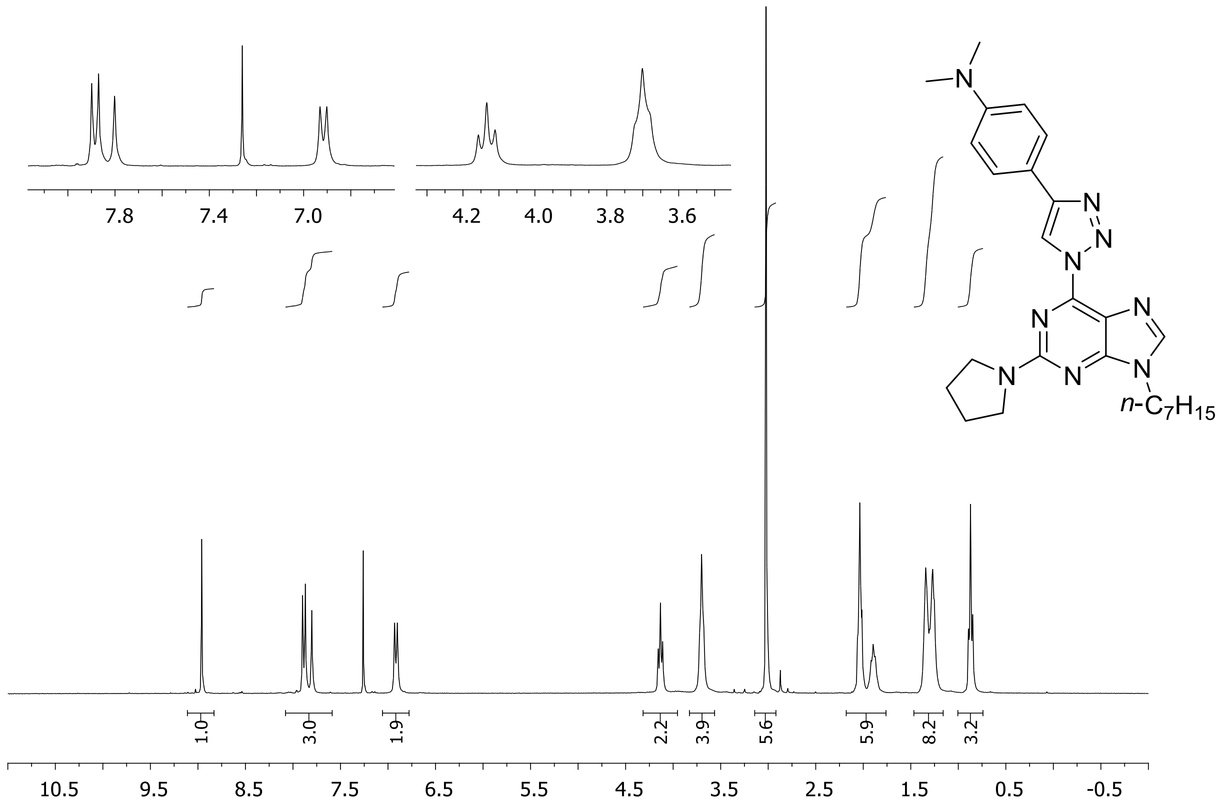

Figure S32: ^{1}H NMR (300 MHz, $CDCl_3$) spectrum.

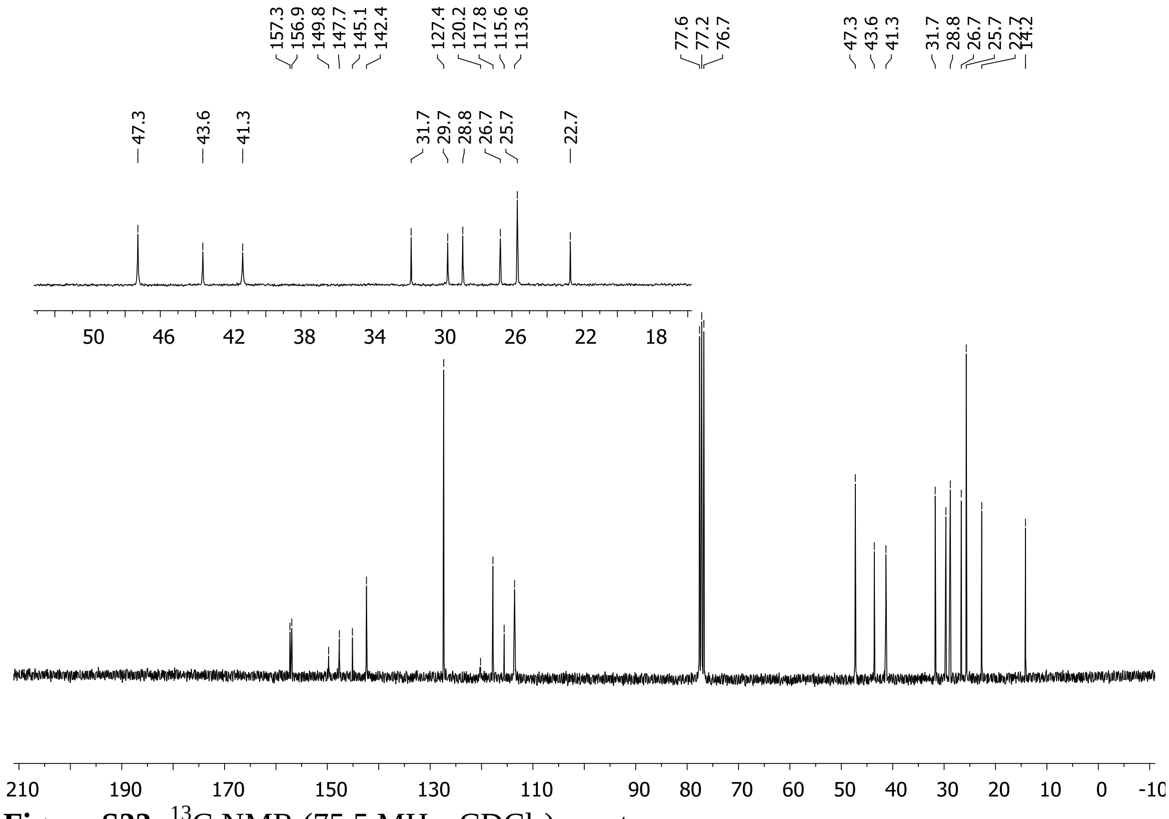

Figure S33: ^{13}C NMR (75.5 MHz, $CDCl_3$) spectrum.

6-(4-(4-Fluorophenyl)-1*H*-1,2,3-triazol-1-yl)-9-heptyl-2-pyrrolidino-9*H*-purine (7d)

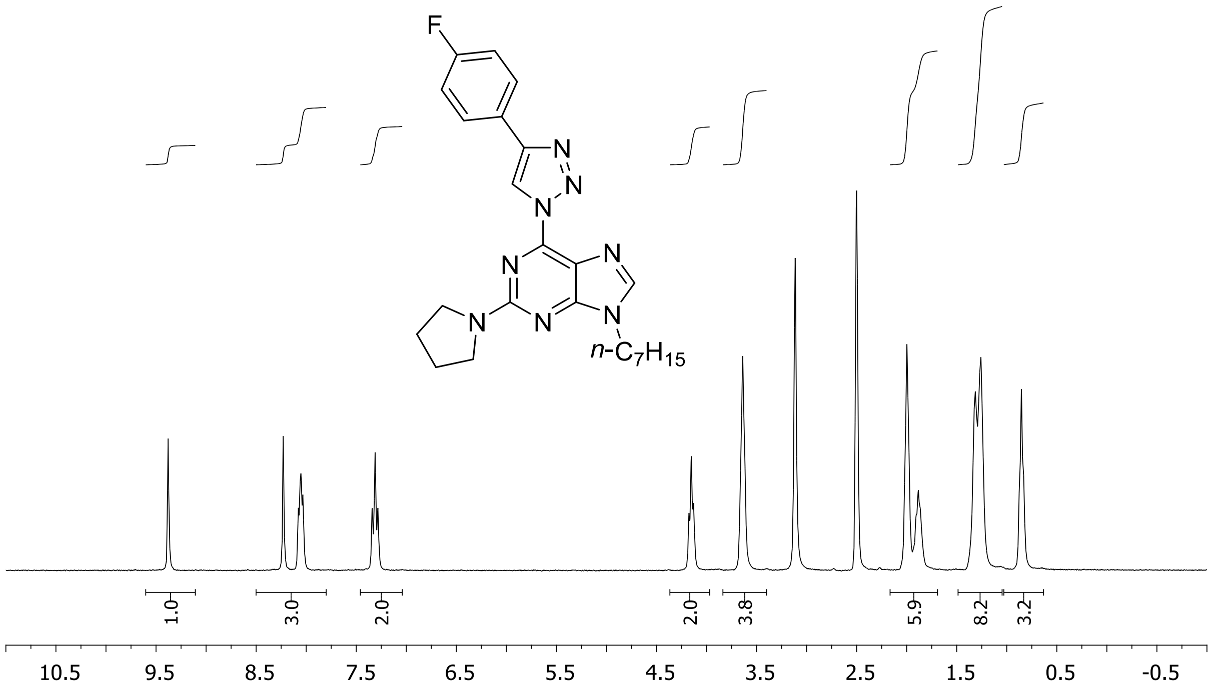

Figure S34: ^{1}H NMR (300 MHz, DMSO-d_6) spectrum.

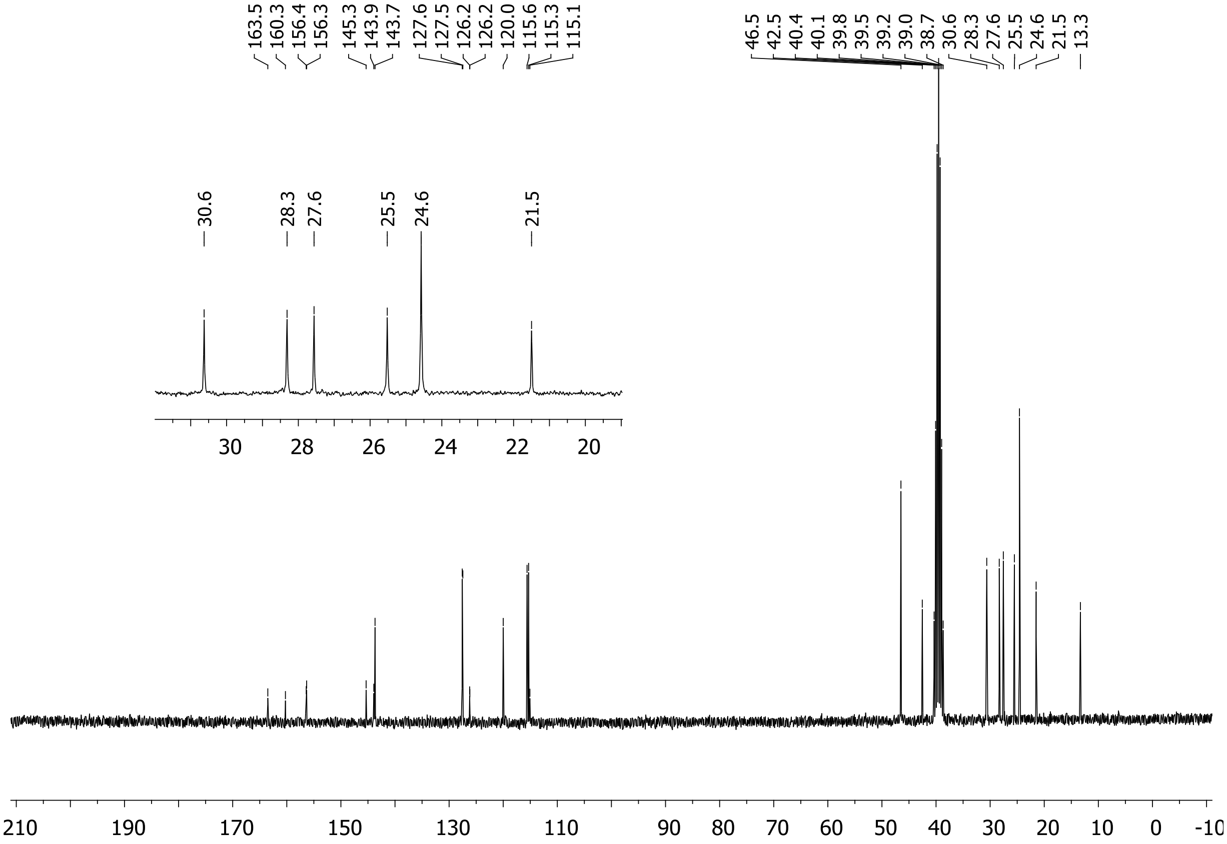

Figure S35: ^{13}C NMR (75.5 MHz, 70 °C, DMSO-d_6) spectrum.

6-(4-(4-Trifluoromethylphenyl)-1*H*-1,2,3-triazol-1-yl)-9-heptyl-2-pyrrolidino-9*H*-purine (7e)

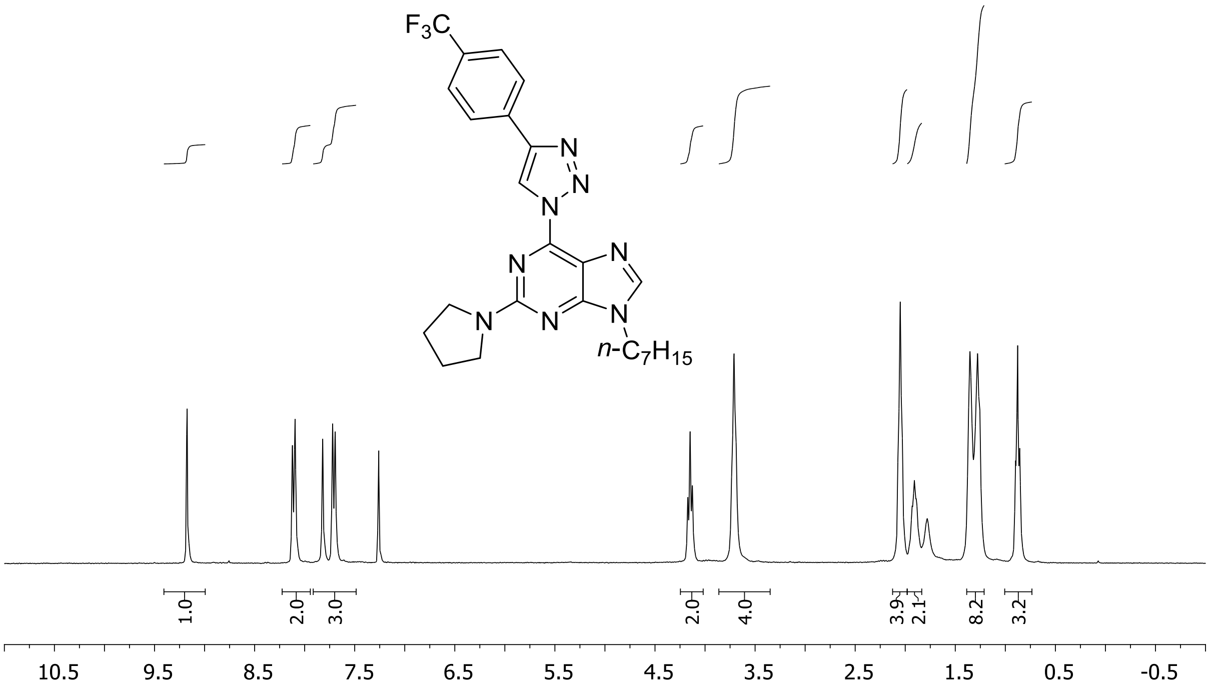

Figure S36: ^{1}H NMR (300 MHz, $CDCl_3$) spectrum.

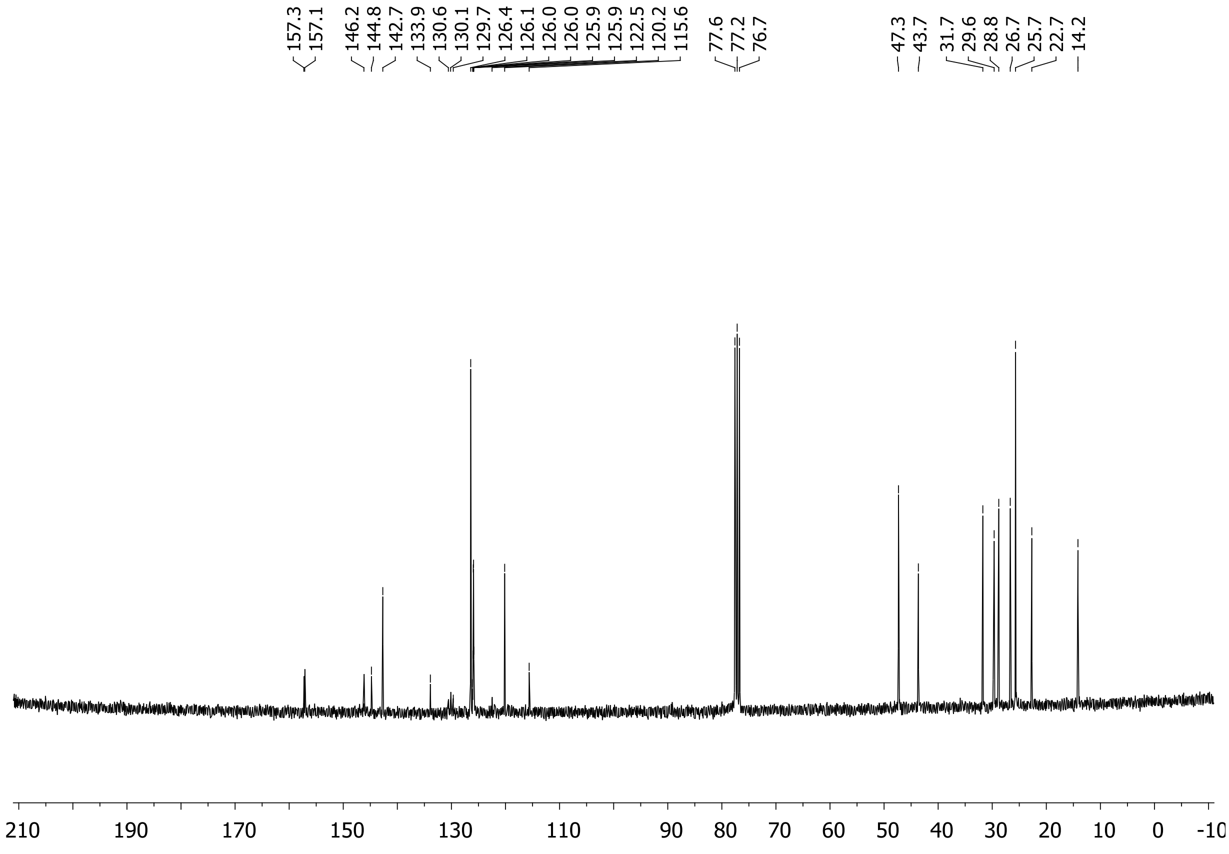

Figure S37: ^{13}C NMR (75.5 MHz, $CDCl_3$) spectrum.

6-(4-(4-Cianophenyl)-1*H*-1,2,3-triazol-1-yl)-9-heptyl-2-pyrrolidino-9*H*-purine (7f)

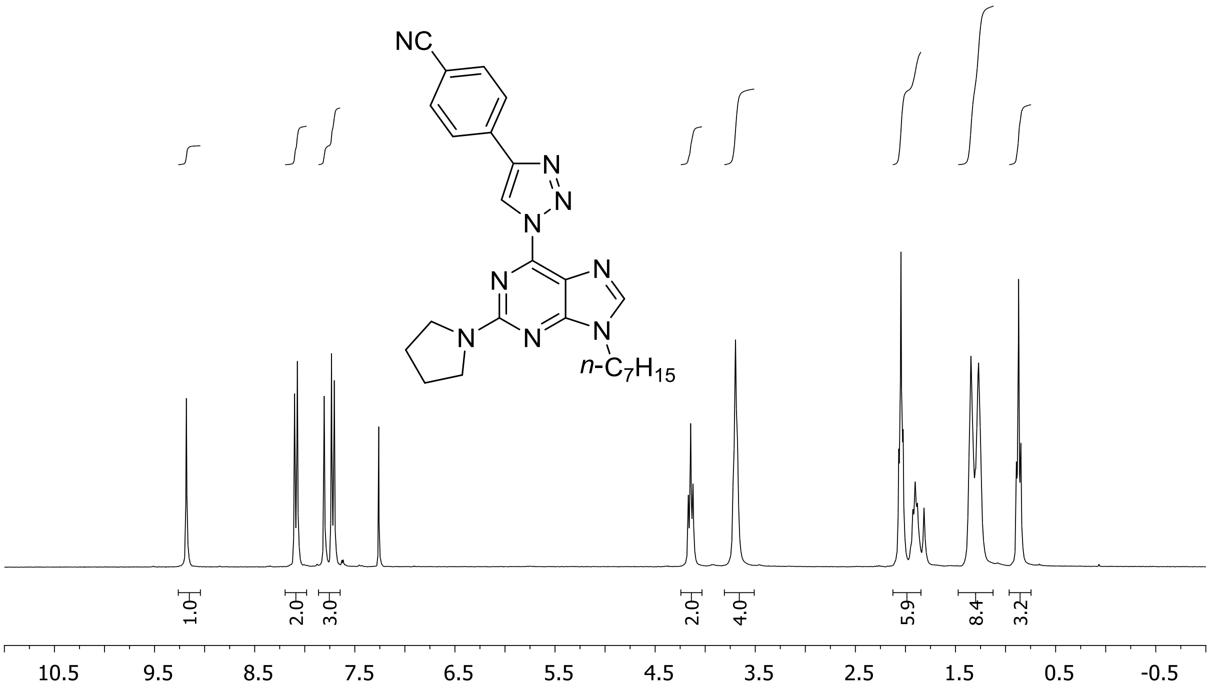

Figure S38: ^{1}H NMR (300 MHz, $CDCl_3$) spectrum.

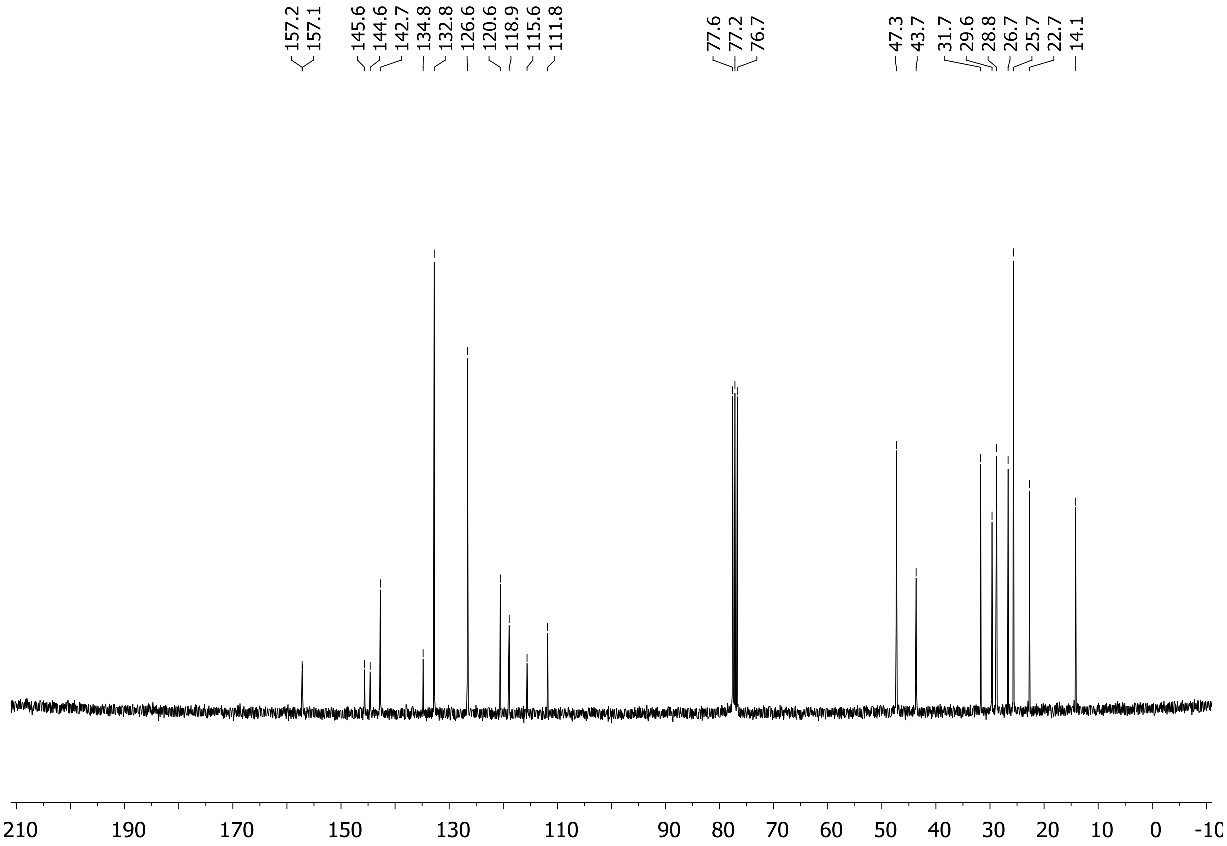

Figure S39: ^{13}C NMR (75.5 MHz, $CDCl_3$) spectrum.

9-Heptyl-6-(4-phenyl-1*H*-1,2,3-triazol-1-yl)-2-piperidino-9*H*-purine (8a)

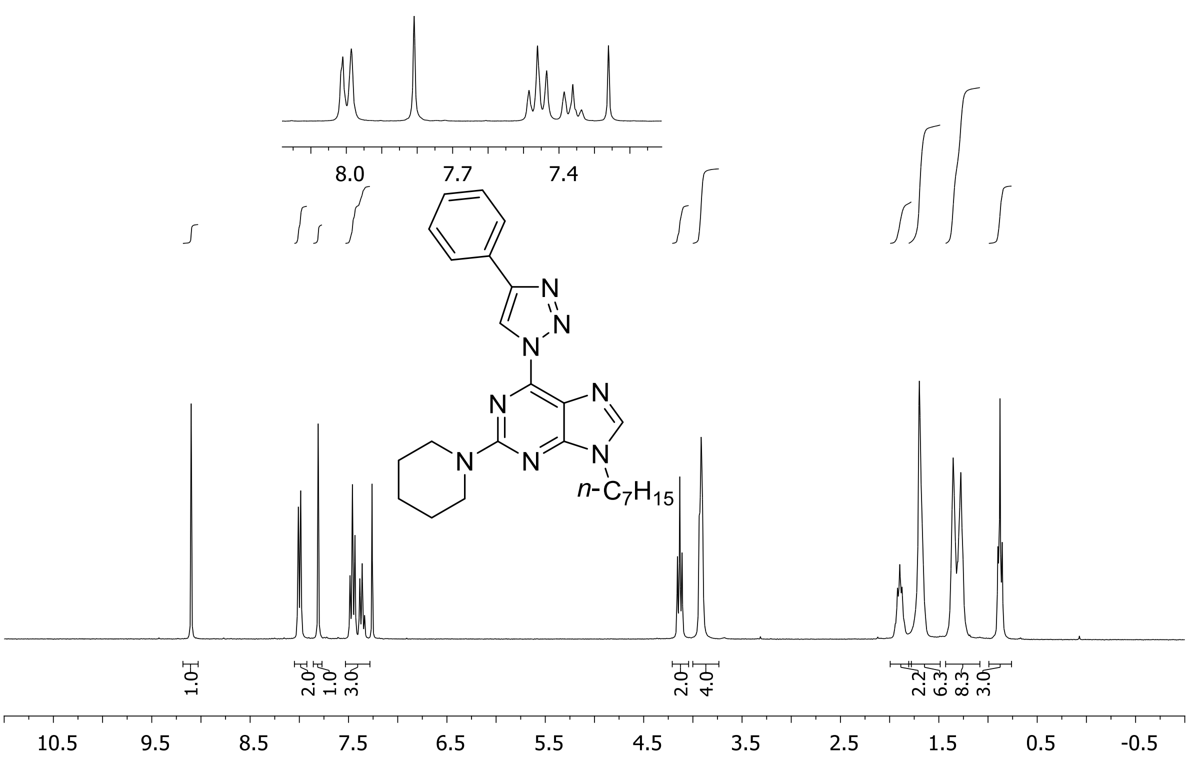

Figure S40: ^{1}H NMR (300 MHz, $CDCl_3$) spectrum.

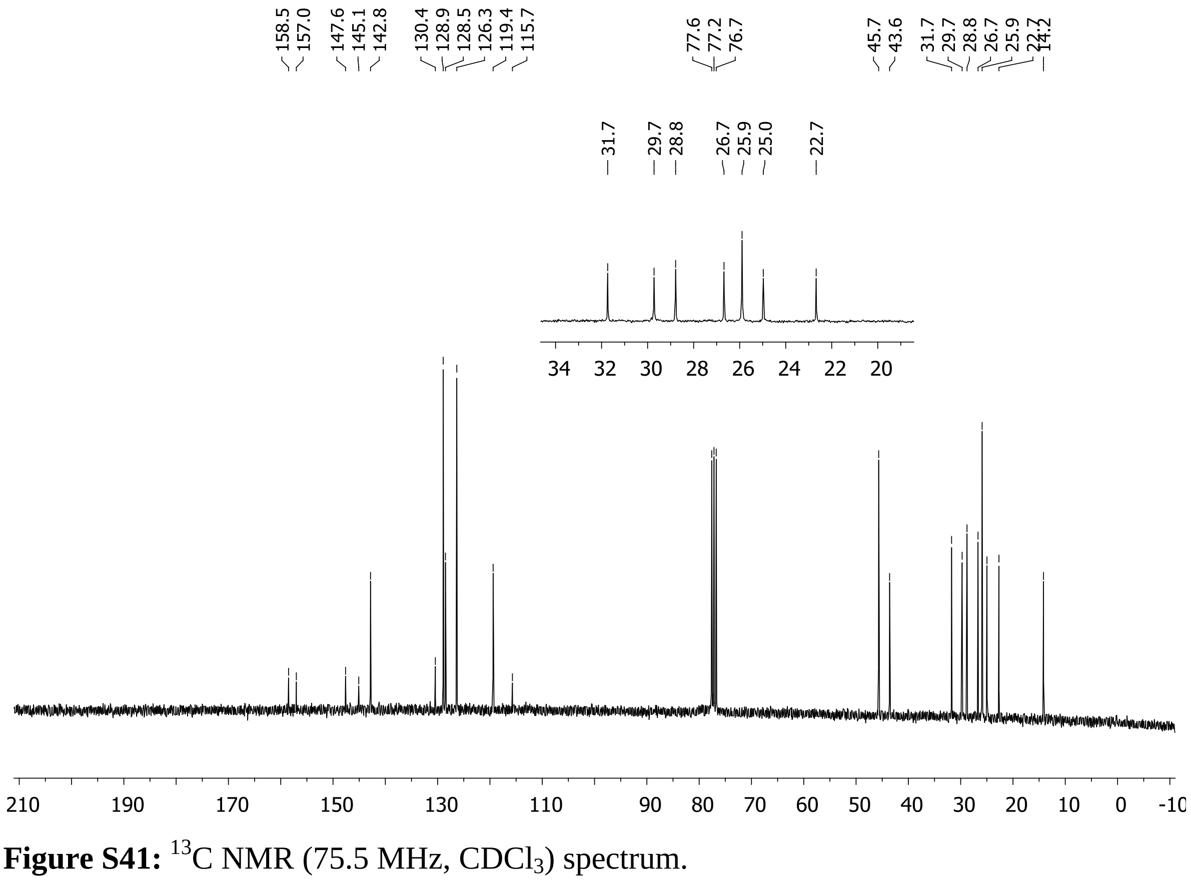

Figure S41: ^{13}C NMR (75.5 MHz, $CDCl_3$) spectrum.

9-Heptyl-6-(4-(4-metoxyphenyl)-1*H*-1,2,3-triazol-1-yl)-2-piperidino-9*H*-purine (8b)

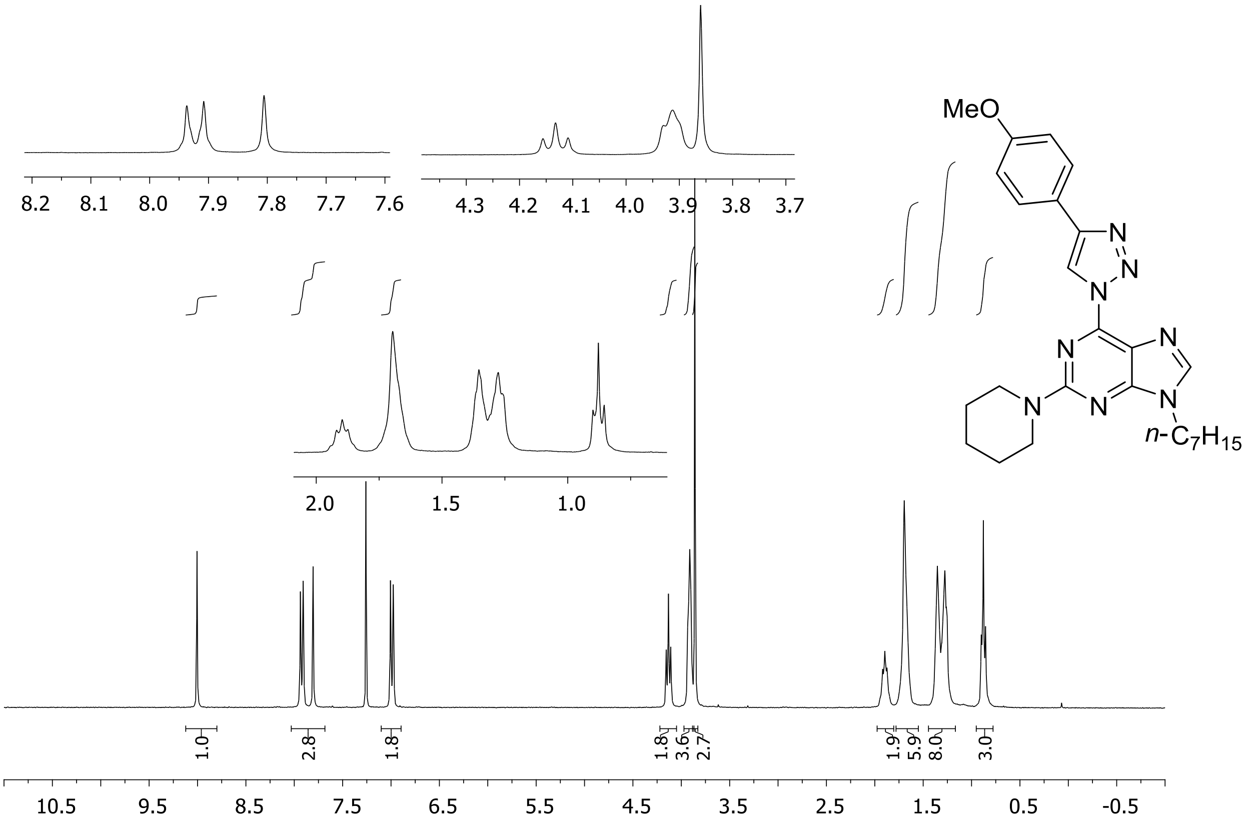

Figure S42: ^{1}H NMR (300 MHz, $CDCl_3$) spectrum.

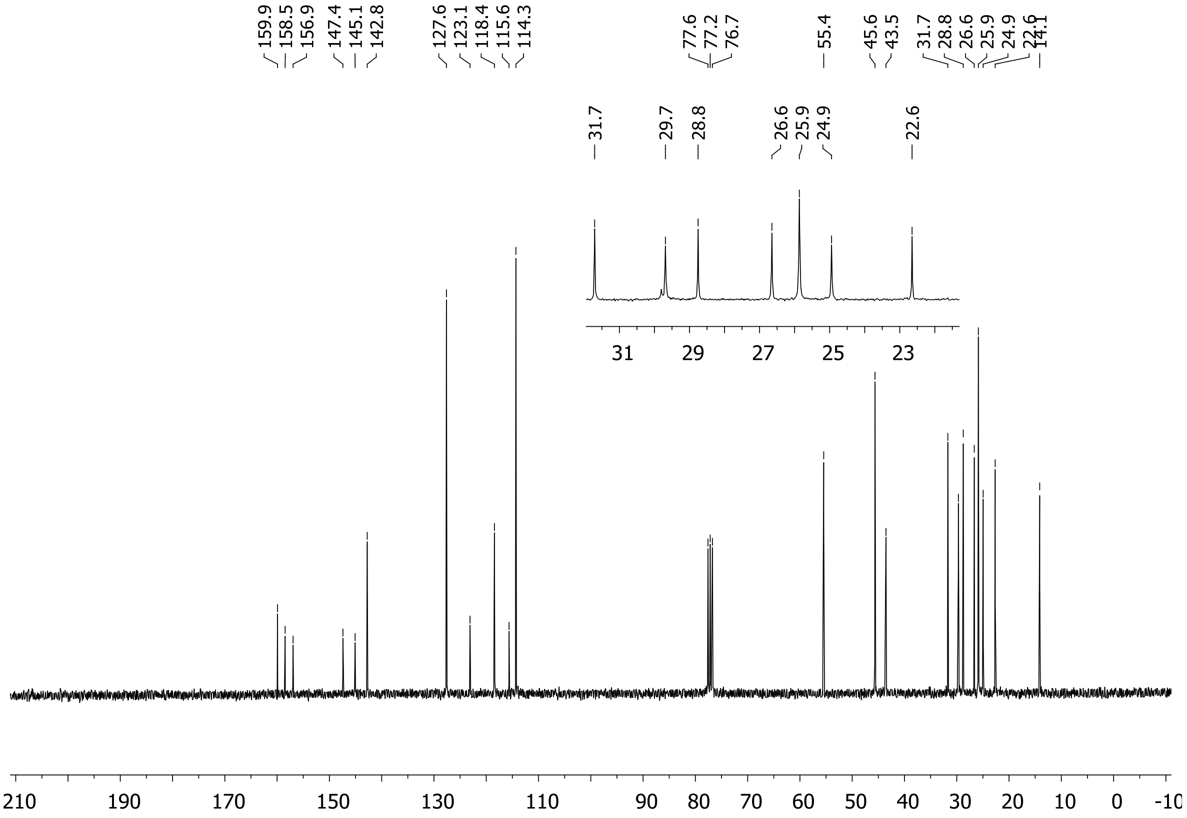

Figure S43: ^{13}C NMR (75.5 MHz, $CDCl_3$) spectrum.

9-Heptyl-6-(4-(4-dimethylaminophenyl)-1*H*-1,2,3-triazol-1-yl)-2-piperidino-9*H*-purine (8c)

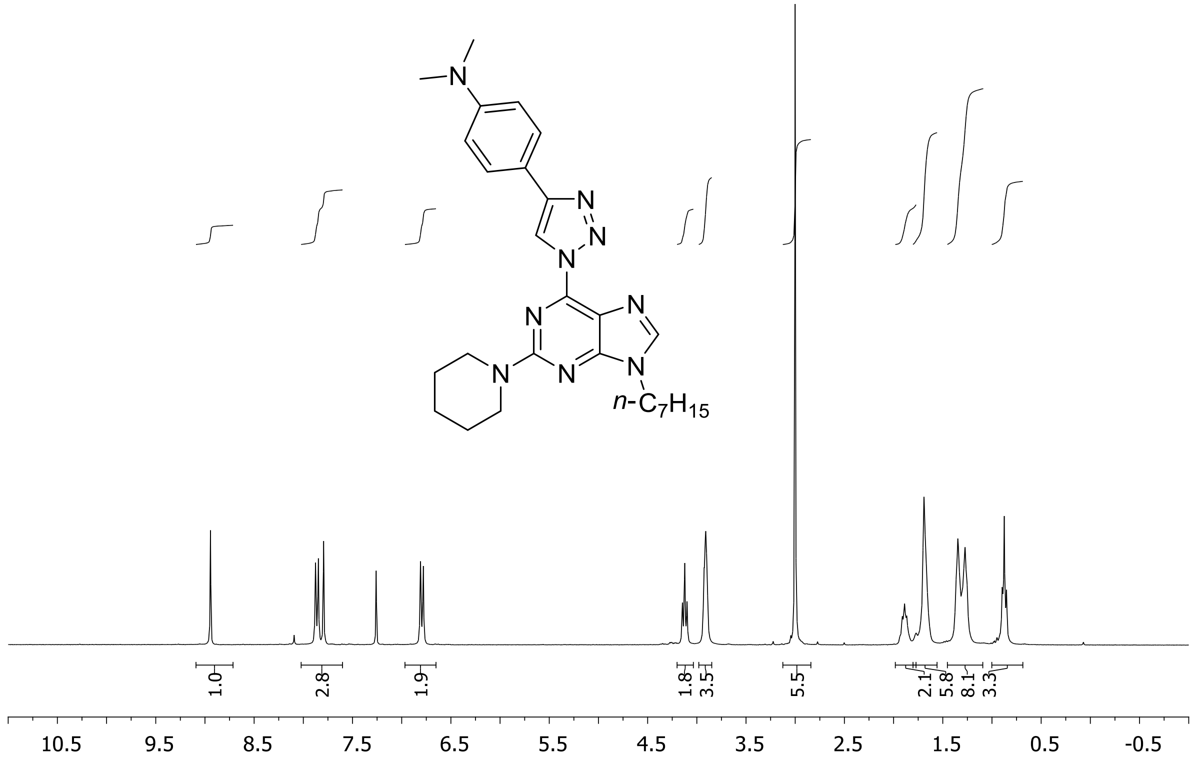

Figure S44: ^{1}H NMR (300 MHz, $CDCl_3$) spectrum.

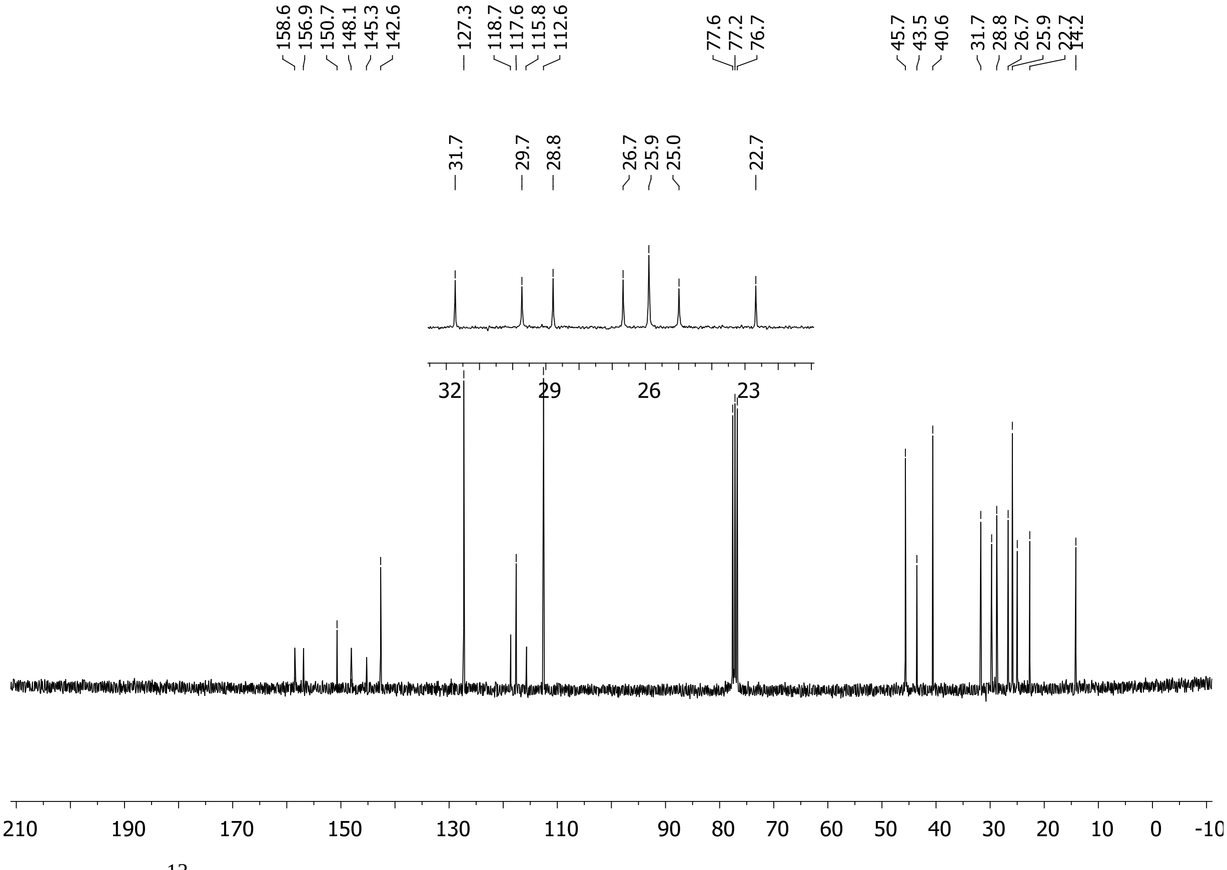

Figure S45: ^{13}C NMR (75.5 MHz, $CDCl_3$) spectrum.

6-(4-(4-Fluorophenyl)-1*H*-1,2,3-triazol-1-yl)-9-heptyl-2-piperidino-9*H*-purine (8d)

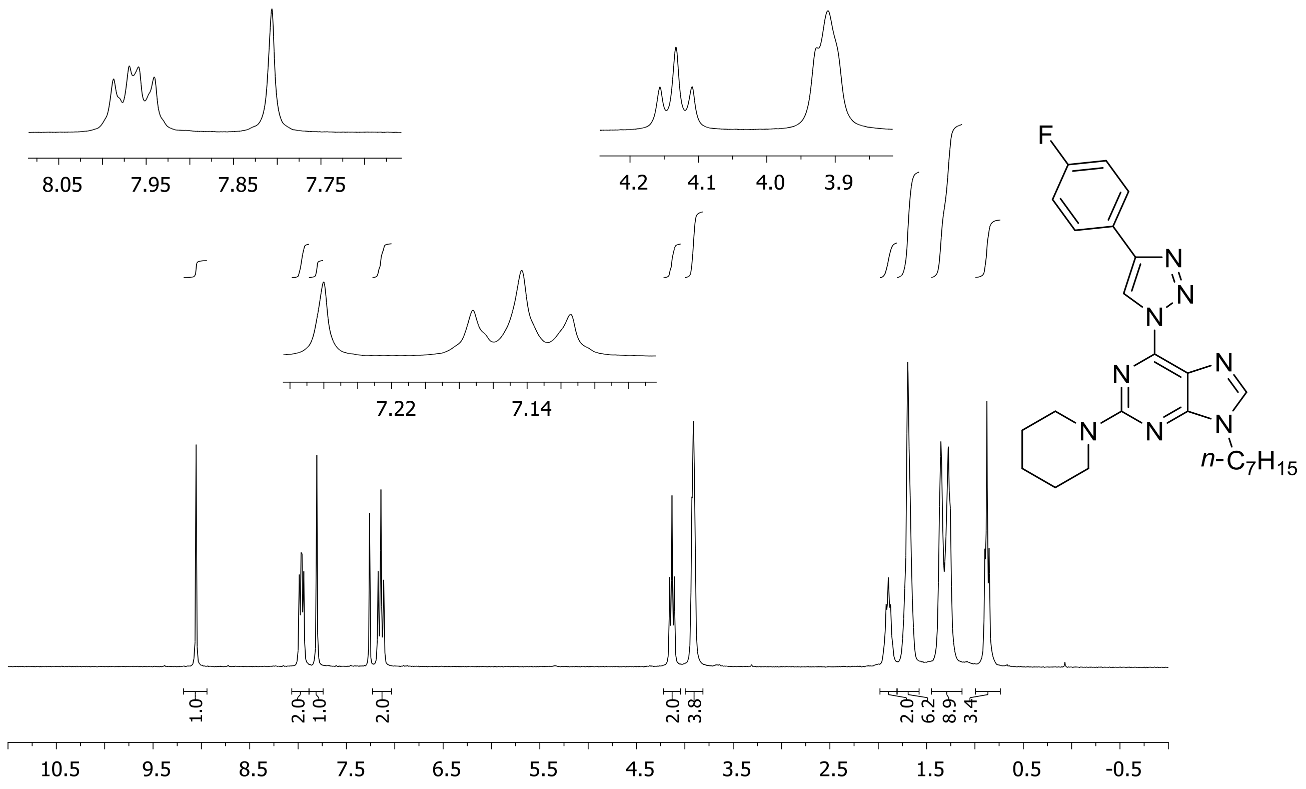

Figure S46: ^{1}H NMR (300 MHz, $CDCl_3$) spectrum.

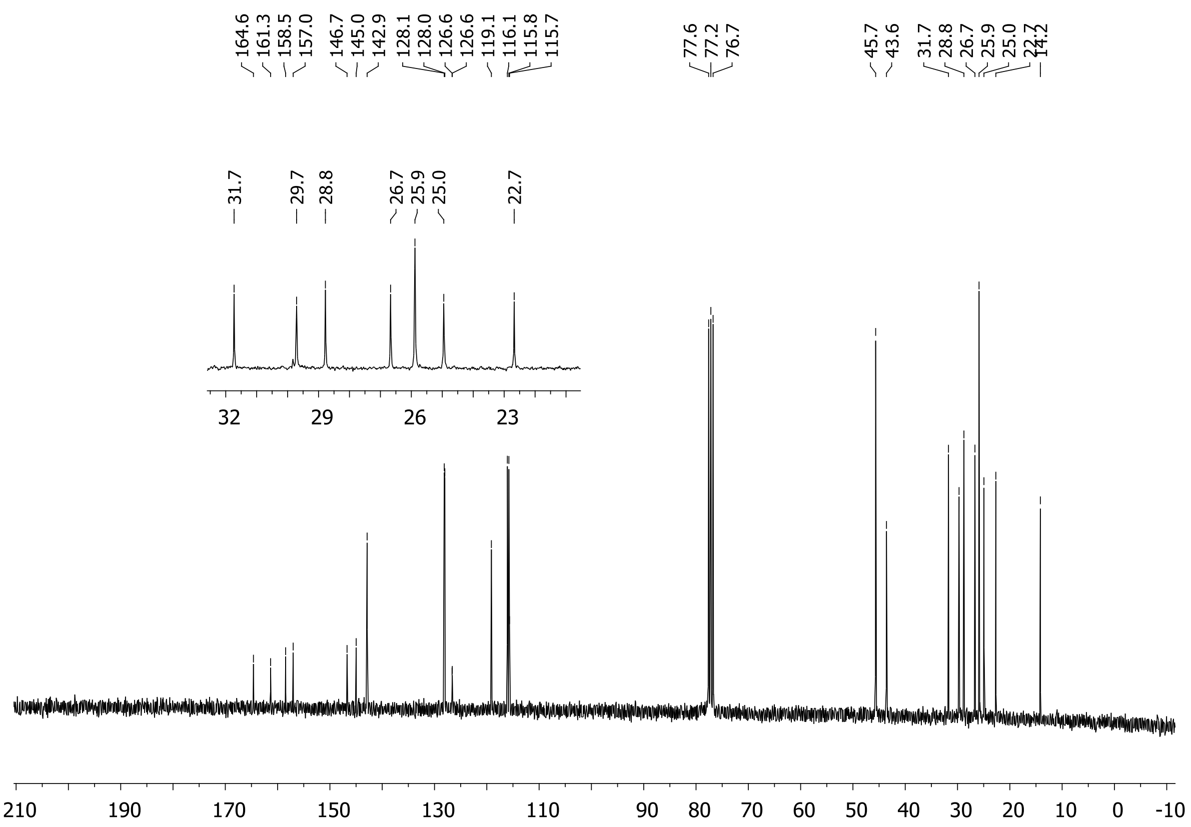

Figure S47: ^{13}C NMR (75.5 MHz, $CDCl_3$) spectrum.

6-(4-(4-Trifluoromethylphenyl)-1*H*-1,2,3-triazol-1-yl)-9-heptyl-2-piperidino-9*H*-purine (8e)

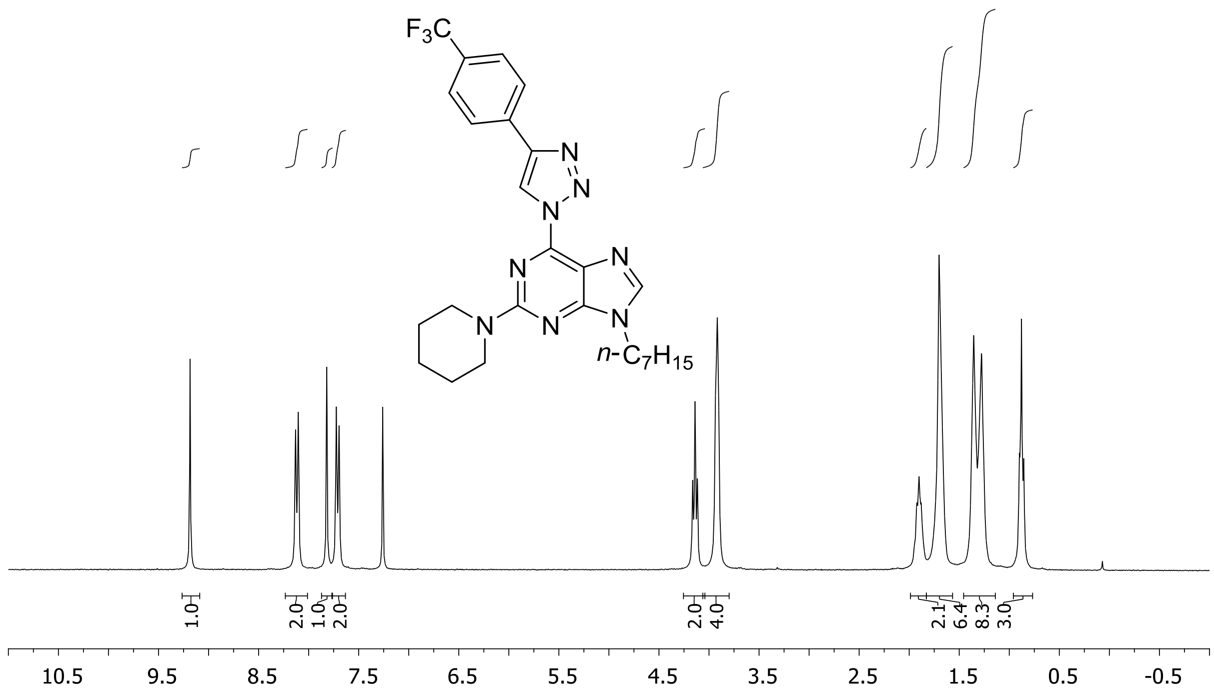

Figure S48: ^{1}H NMR (300 MHz, $CDCl_3$) spectrum.

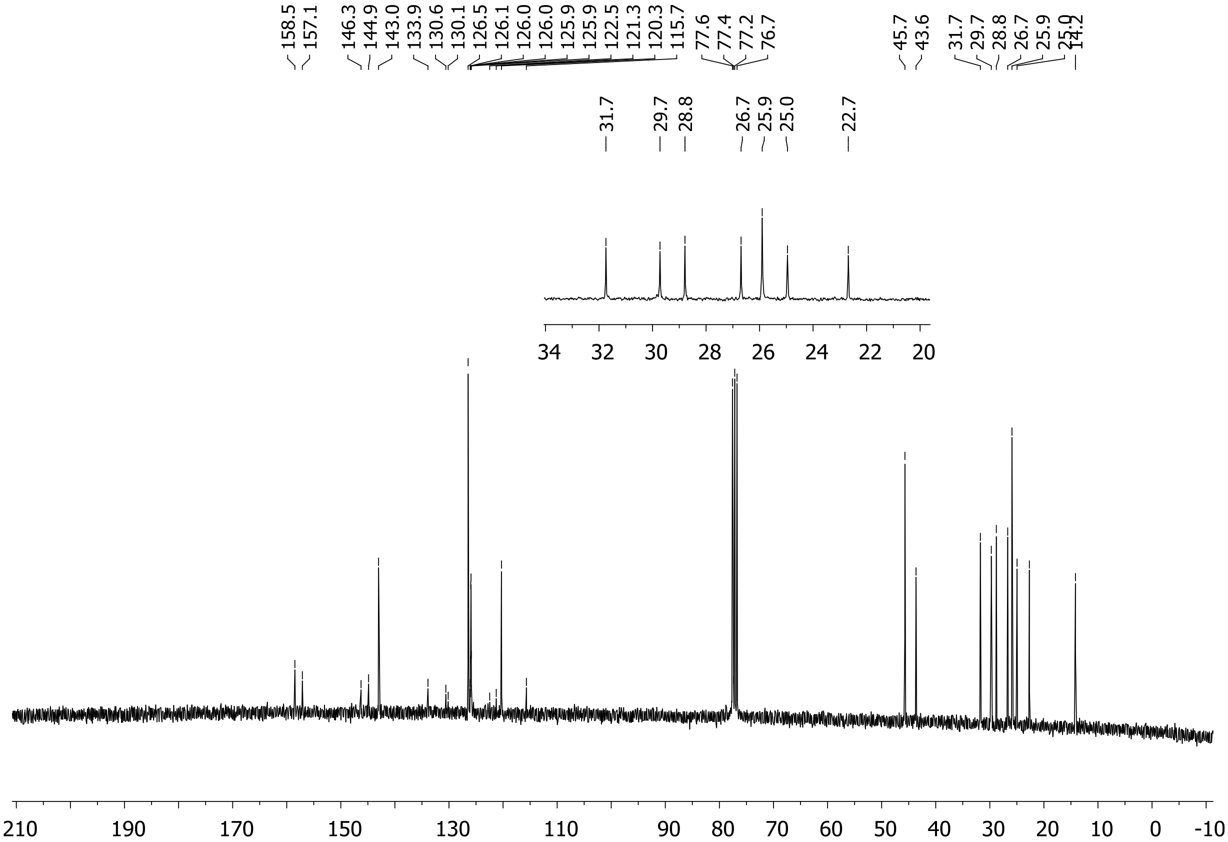

Figure S49: ^{13}C NMR (75.5 MHz, $CDCl_3$) spectrum.

6-(4-(4-Cianophenyl)-1*H*-1,2,3-triazol-1-yl)-9-heptyl-2-piperidino-9*H*-purine (8f)

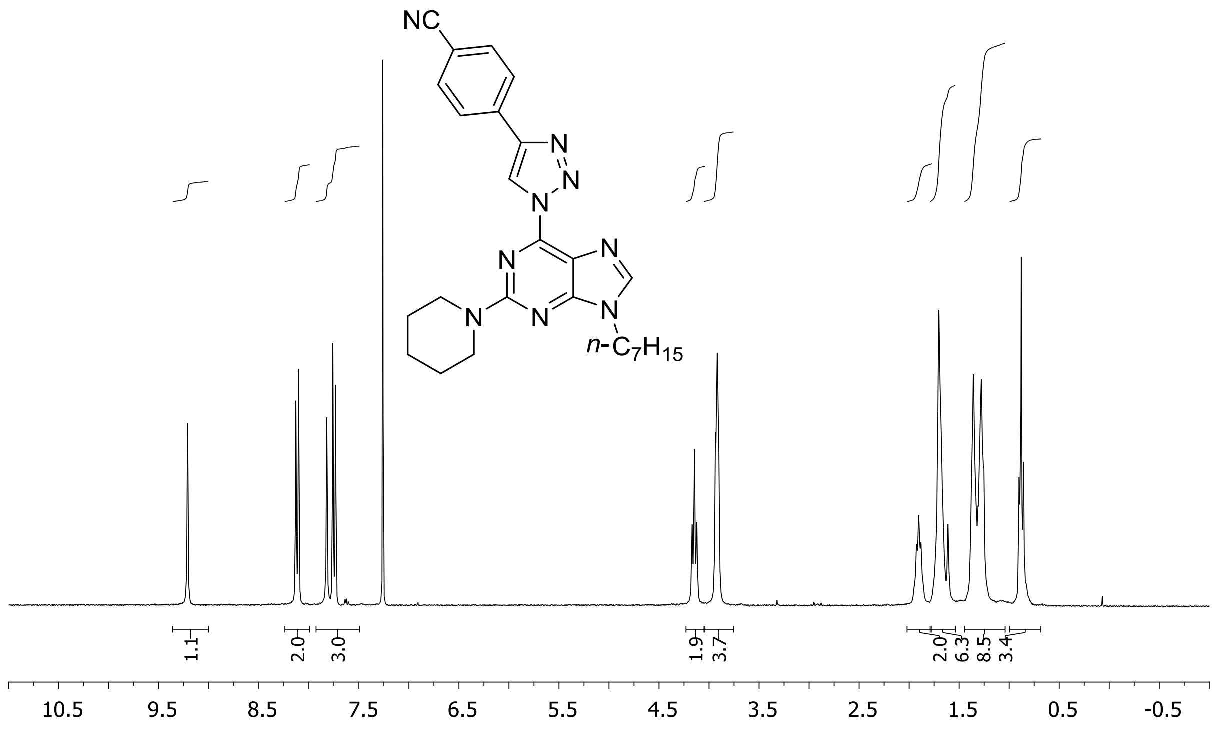

Figure S50: ^{1}H NMR (300 MHz, $CDCl_3$) spectrum.

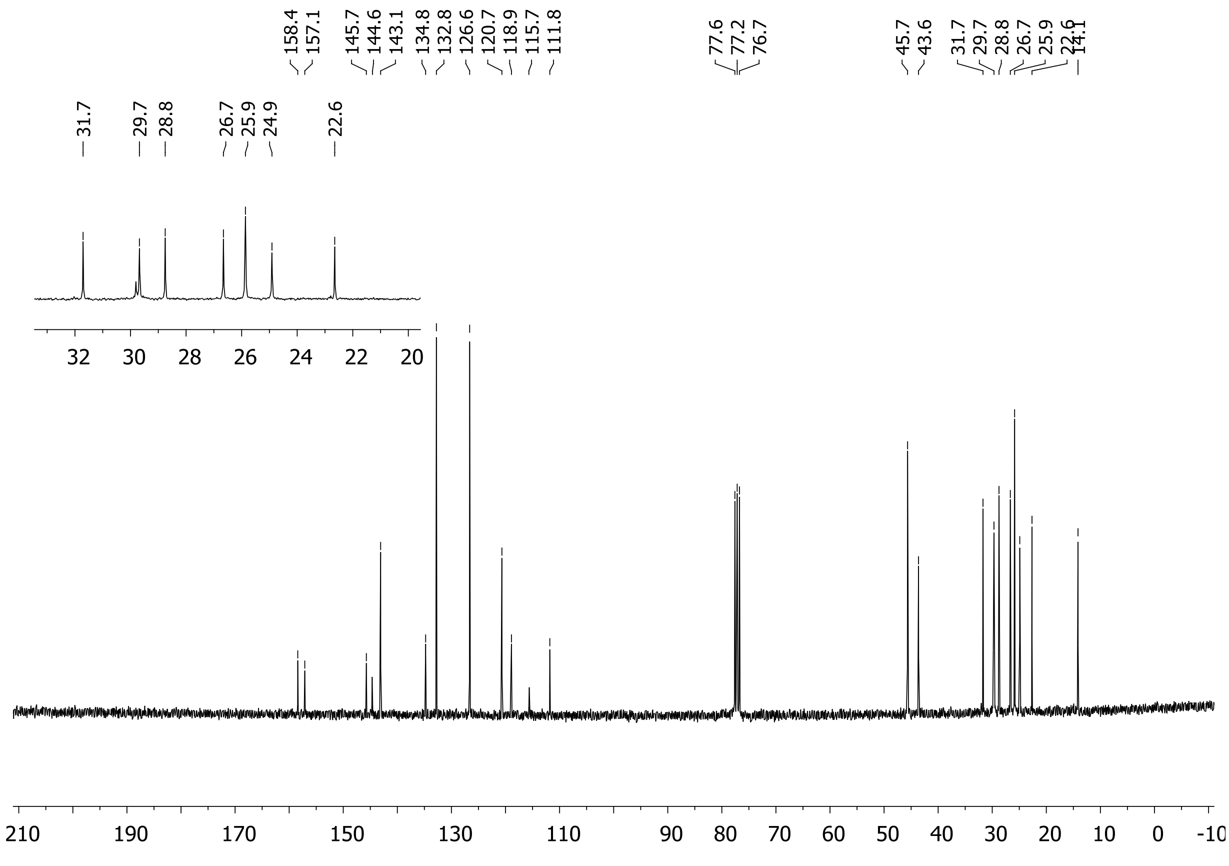

Figure S51: ^{13}C NMR (75.5 MHz, $CDCl_3$) spectrum.

9-Pentyl-6-(4-phenyl-1*H*-1,2,3-triazol-1-yl)-2-piperidino-9*H*-purine (9)

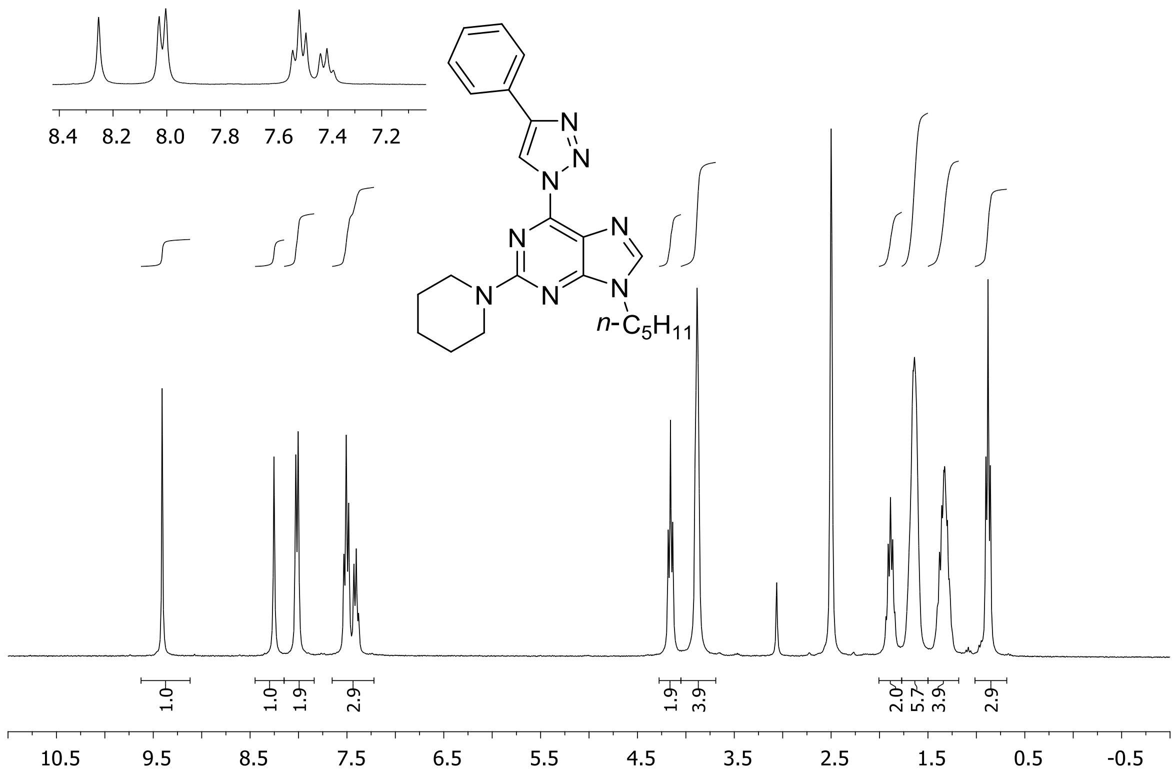

Figure S52: ^{1}H NMR (300 MHz, 70 °C, DMSO-d_6) spectrum.

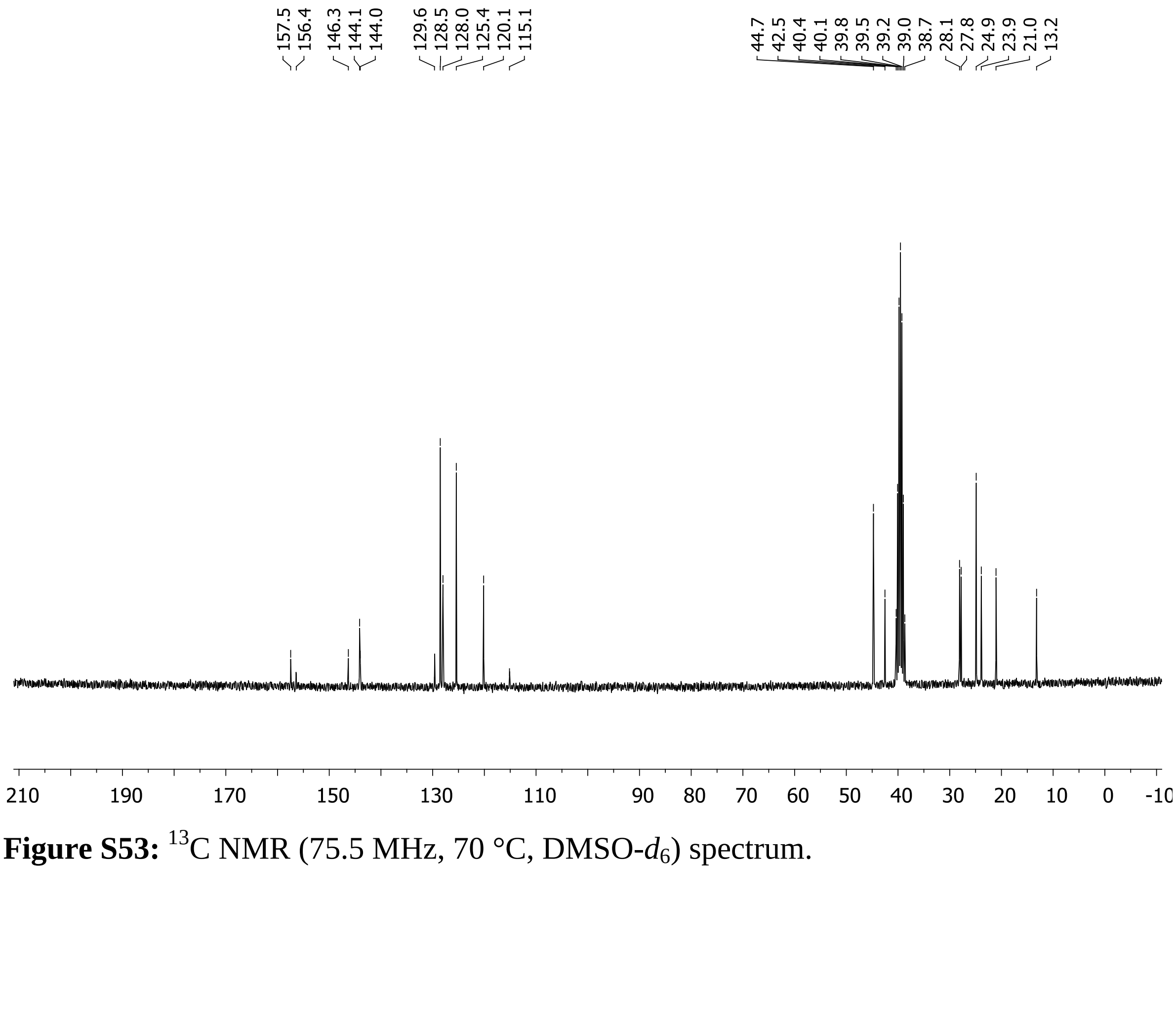

Figure S53: ^{13}C NMR (75.5 MHz, 70 °C, DMSO-d_6) spectrum.

Copies of ^{1}H and ^{13}C NMR spectra of 7-deazapurines (**10a–f**, **11a–f**)

6-(4-Phenyl-1,2,3-triazol-1-yl)-9-methyl-2-pyrrolidino-7-deazapurine (10a)

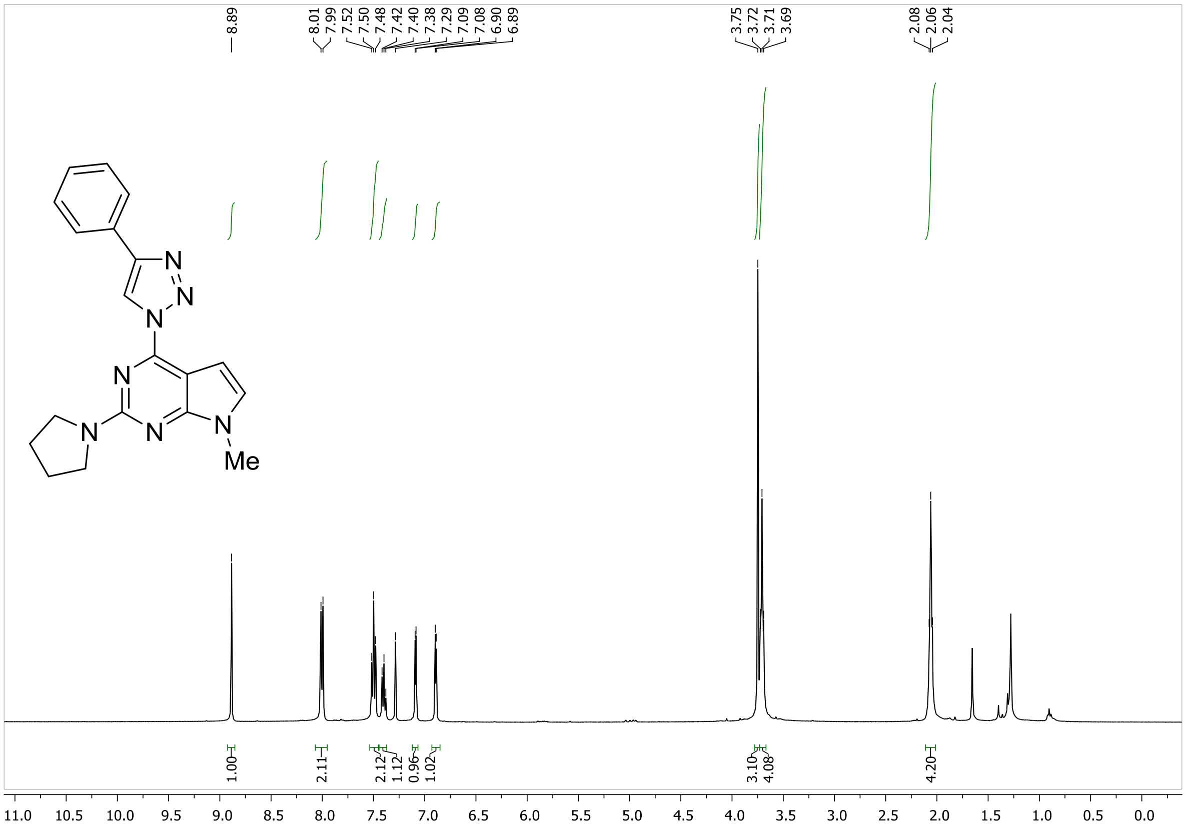

Figure S54: ^{1}H NMR (400 MHz, $CDCl_3$) spectrum.

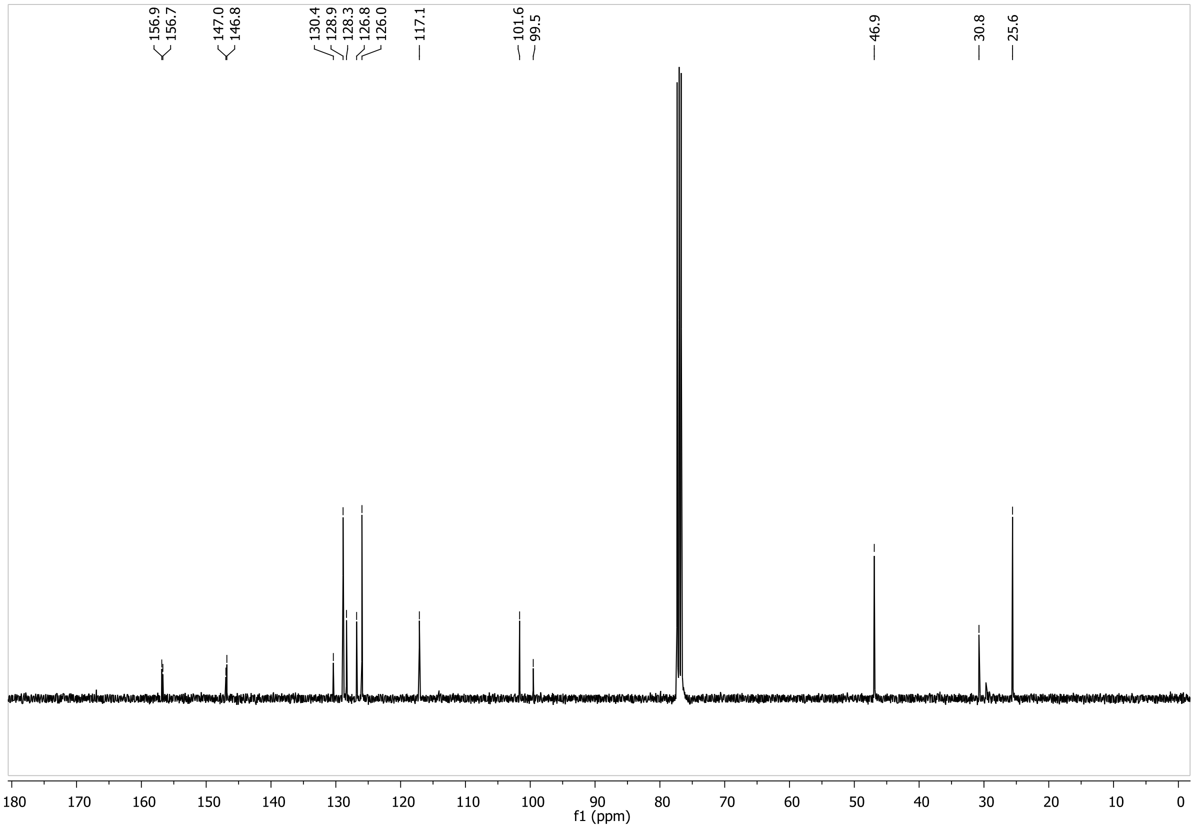

Figure S55: ^{13}C NMR (100 MHz, $CDCl_3$) spectrum.

6-[4-(4-Methoxyphenyl)-1,2,3-triazol-1-yl]-9-methyl-2-pyrrolidino-7-deazapurine (10b)

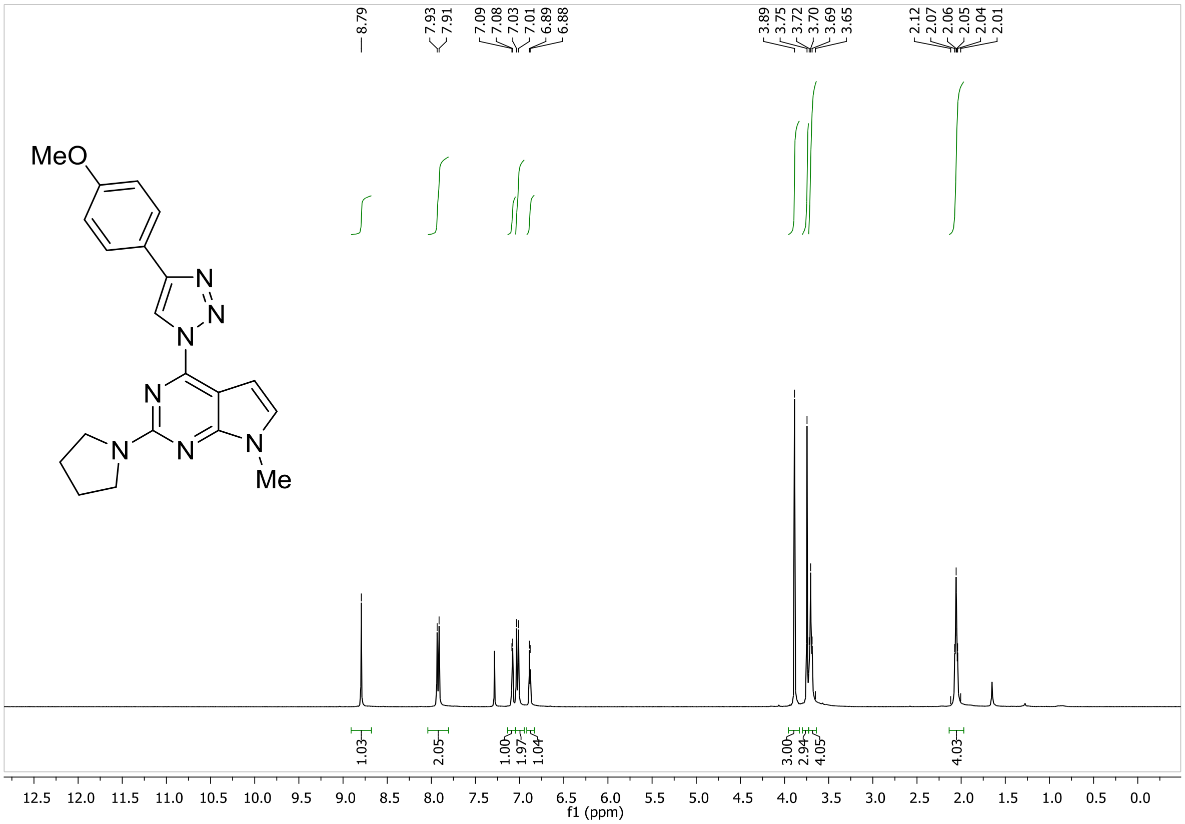

Figure S56: ^{1}H NMR (400 MHz, $CDCl_3$) spectrum.

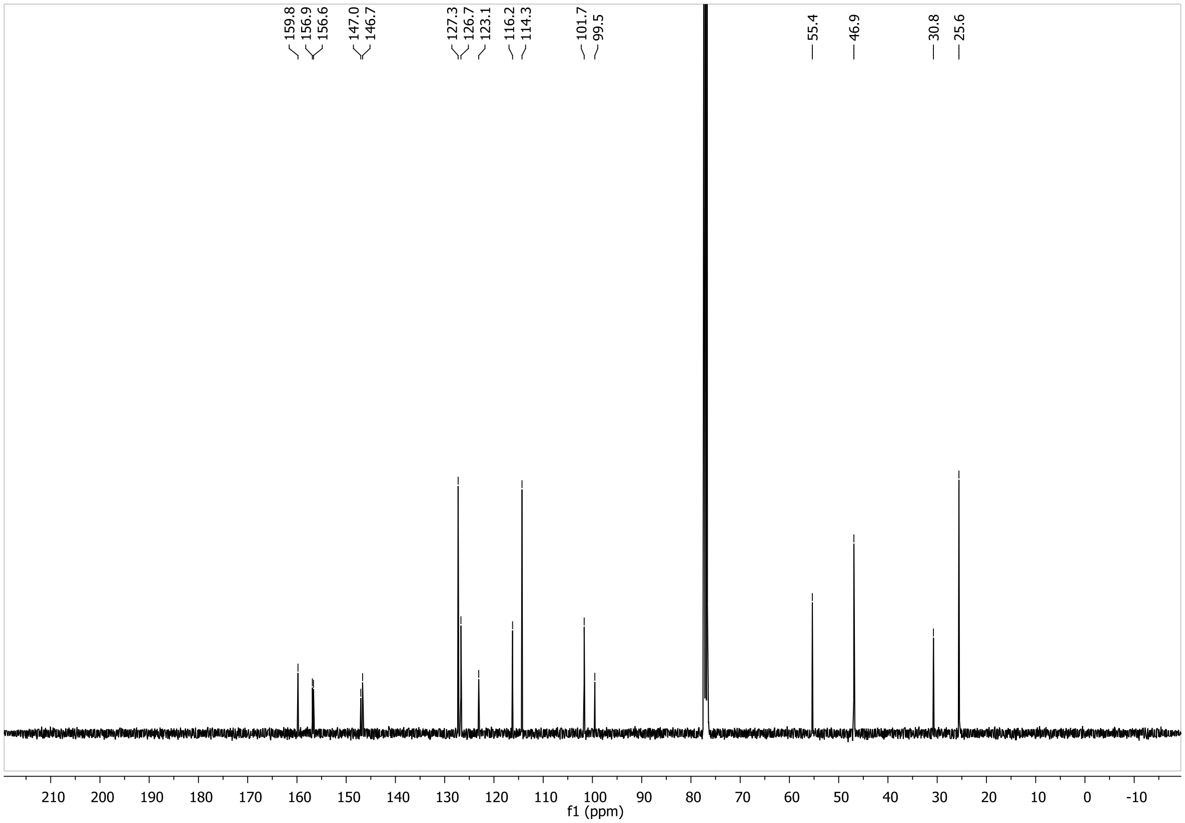

Figure S57: ^{13}C NMR (100 MHz, $CDCl_3$) spectrum.

9-Methyl-6-[4-(4-dimethylaminophenyl)-1,2,3-triazol-1-yl]-2-pyrrolidino-7-deazapurine (10c)

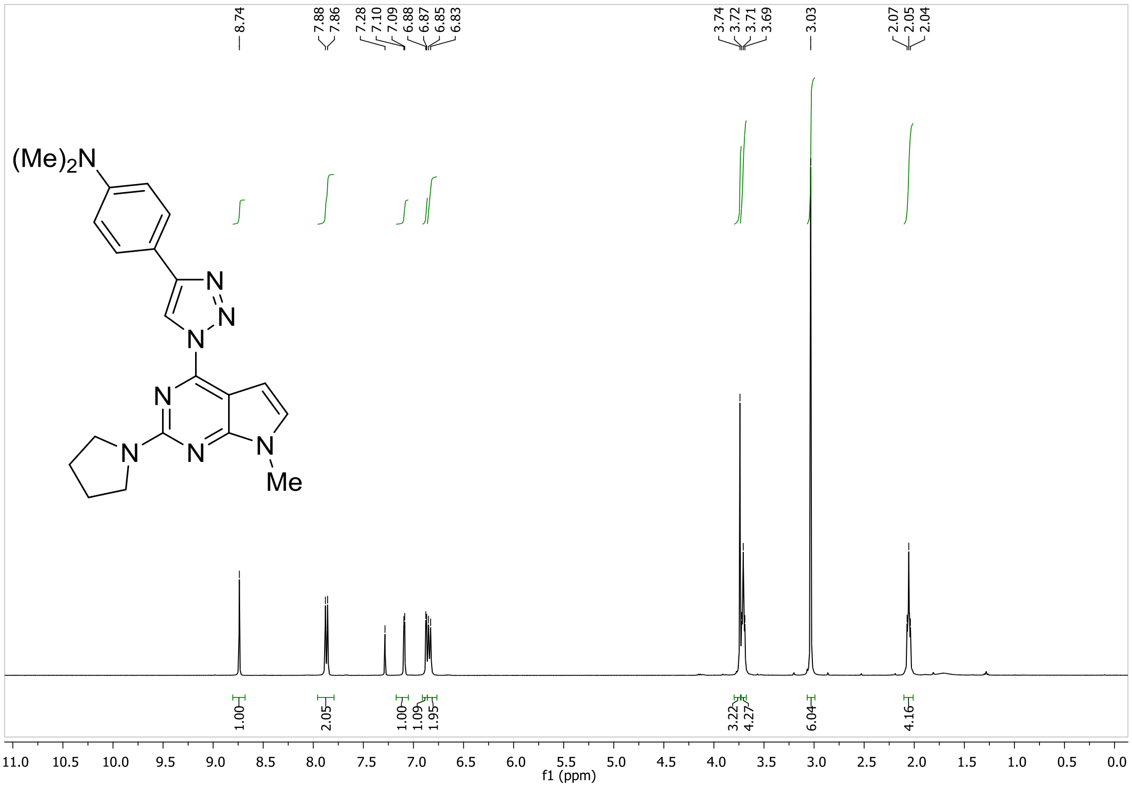

Figure S58: ^{1}H NMR (400 MHz, $CDCl_3$) spectrum.

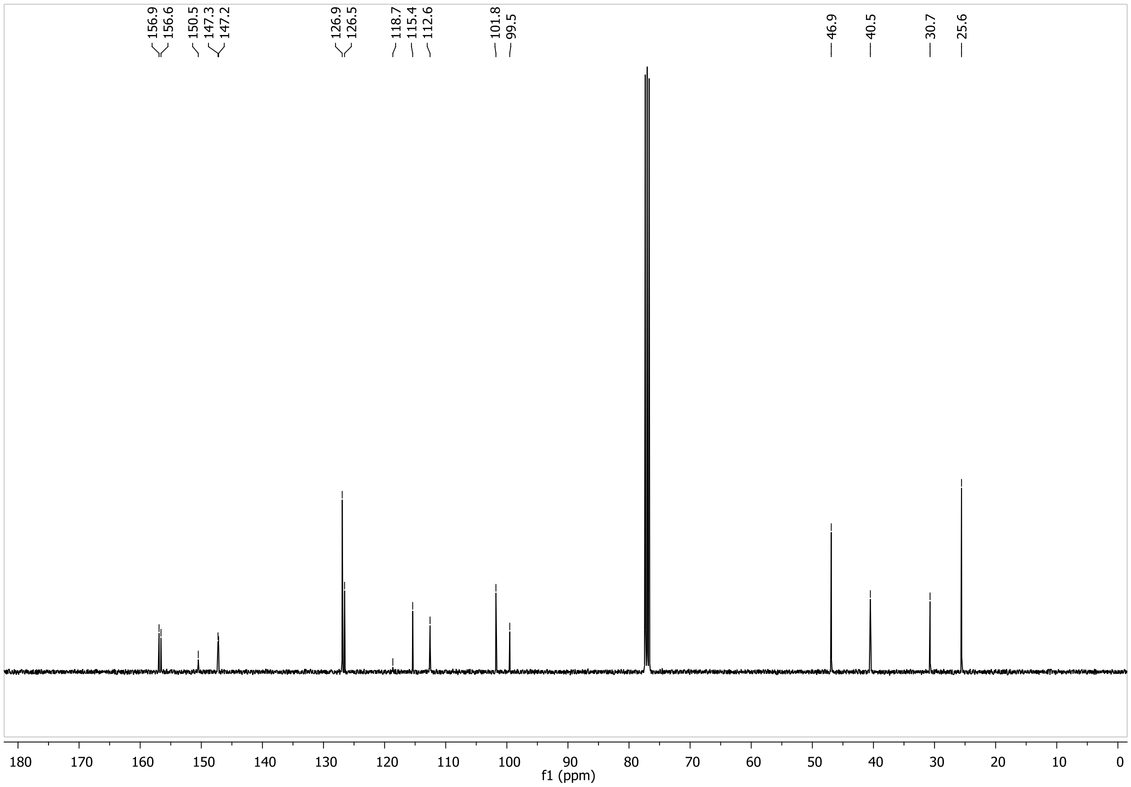

Figure S59: ^{13}C NMR (100 MHz, $CDCl_3$) spectrum.

6-[4-(4-Fluorophenyl)-1,2,3-triazol-1-yl]-9-methyl-2-pyrrolidino-7-deazapurine (10d)

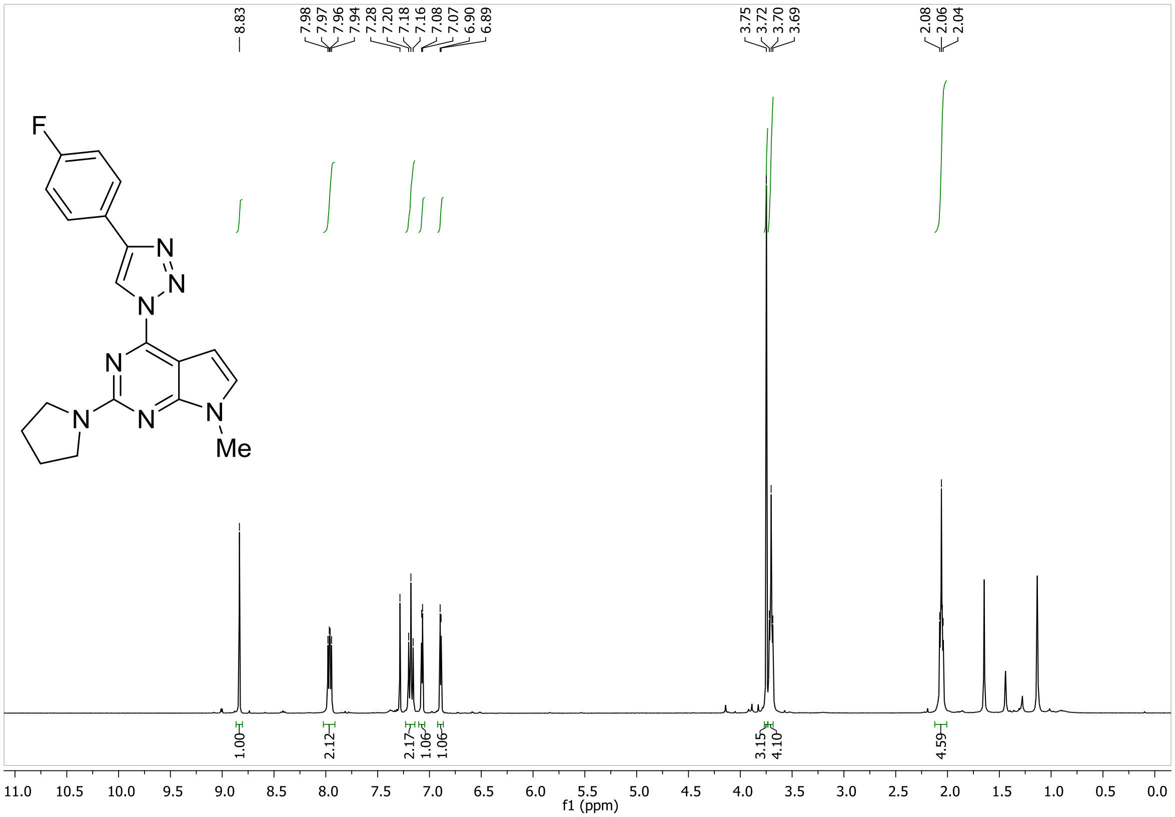

Figure S60: ^{1}H NMR (400 MHz, $CDCl_3$) spectrum.

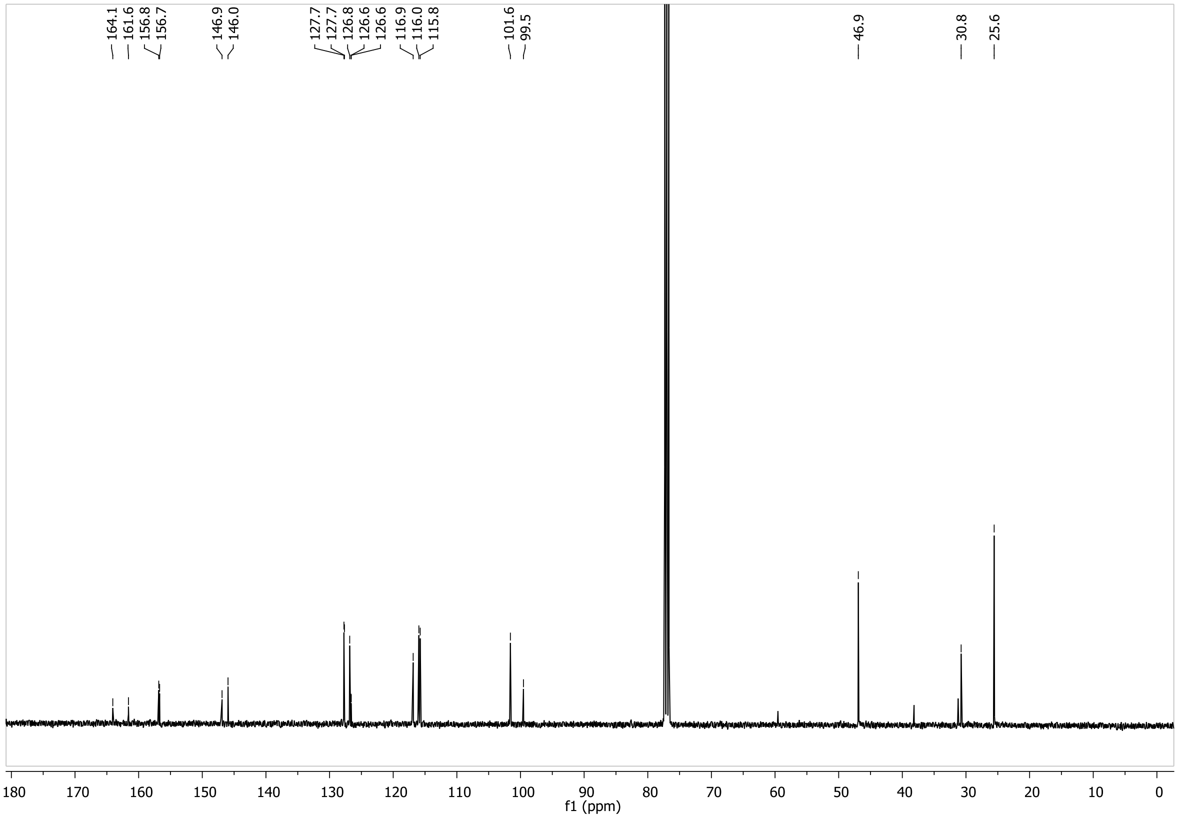

Figure S61: ^{13}C NMR (100 MHz, $CDCl_3$) spectrum.

6-{4-[4-(Trifluoromethyl)phenyl]-1,2,3-triazol-1-yl}-9-methyl-2-pyrrolidino-7-deazapurine (10e)

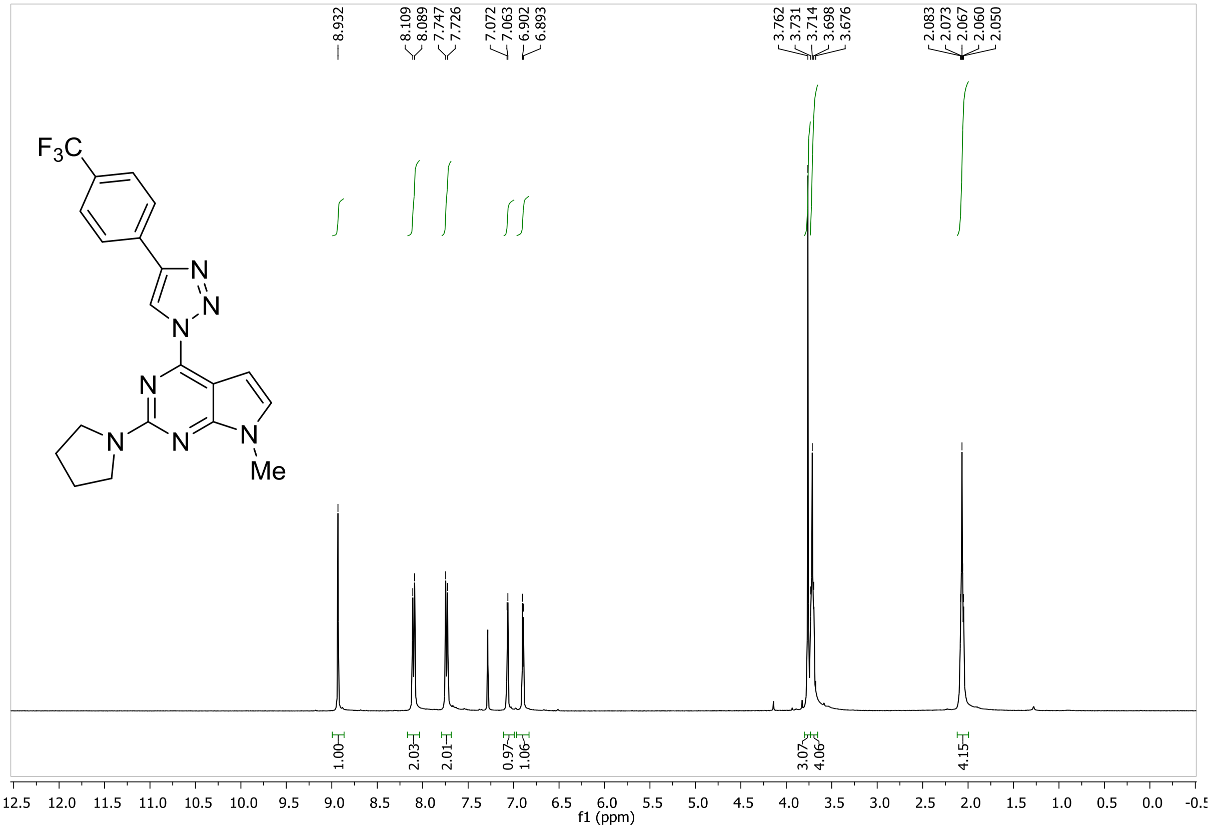

Figure S62: ^{1}H NMR (400 MHz, $CDCl_3$) spectrum.

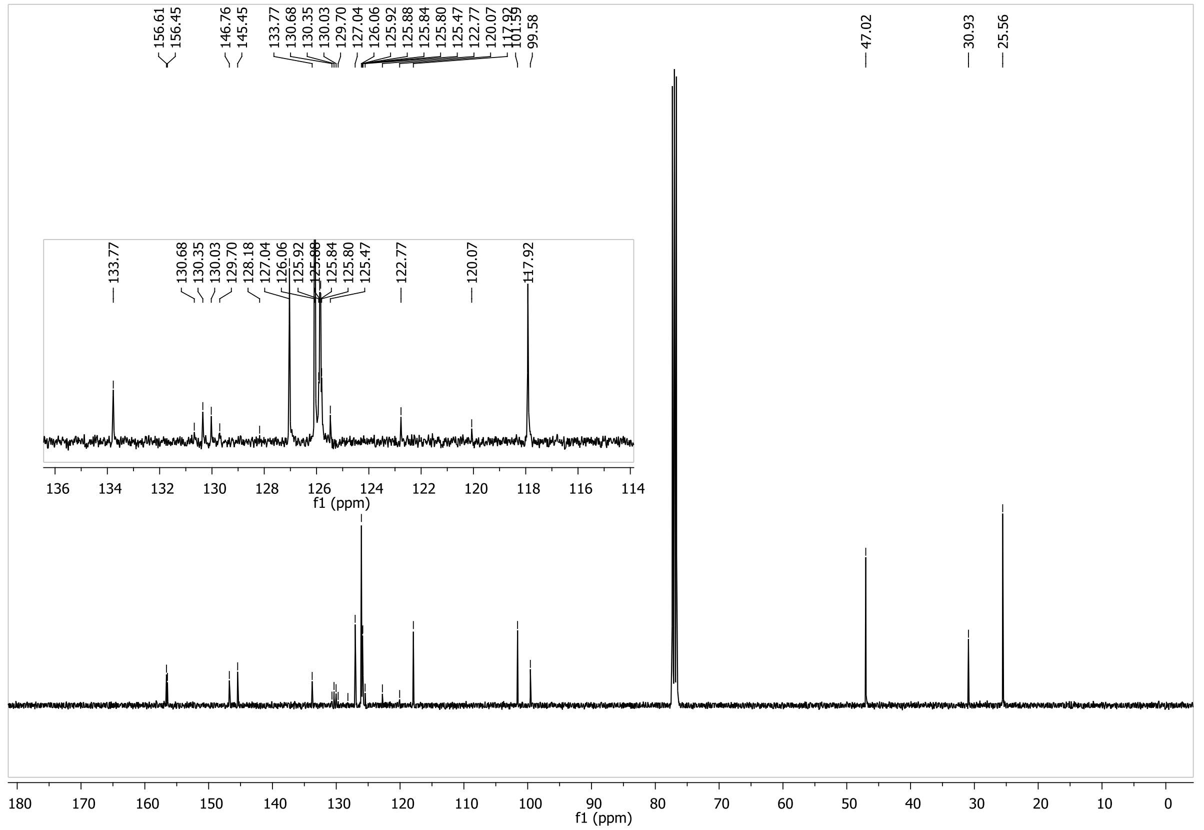

Figure S63: ^{13}C NMR (100 MHz, $CDCl_3$) spectrum.

6-[4-(4-Cyanophenyl)-1,2,3-triazol-1-yl]-9-methyl-2-pyrrolidino-7-deazapurine (10f)

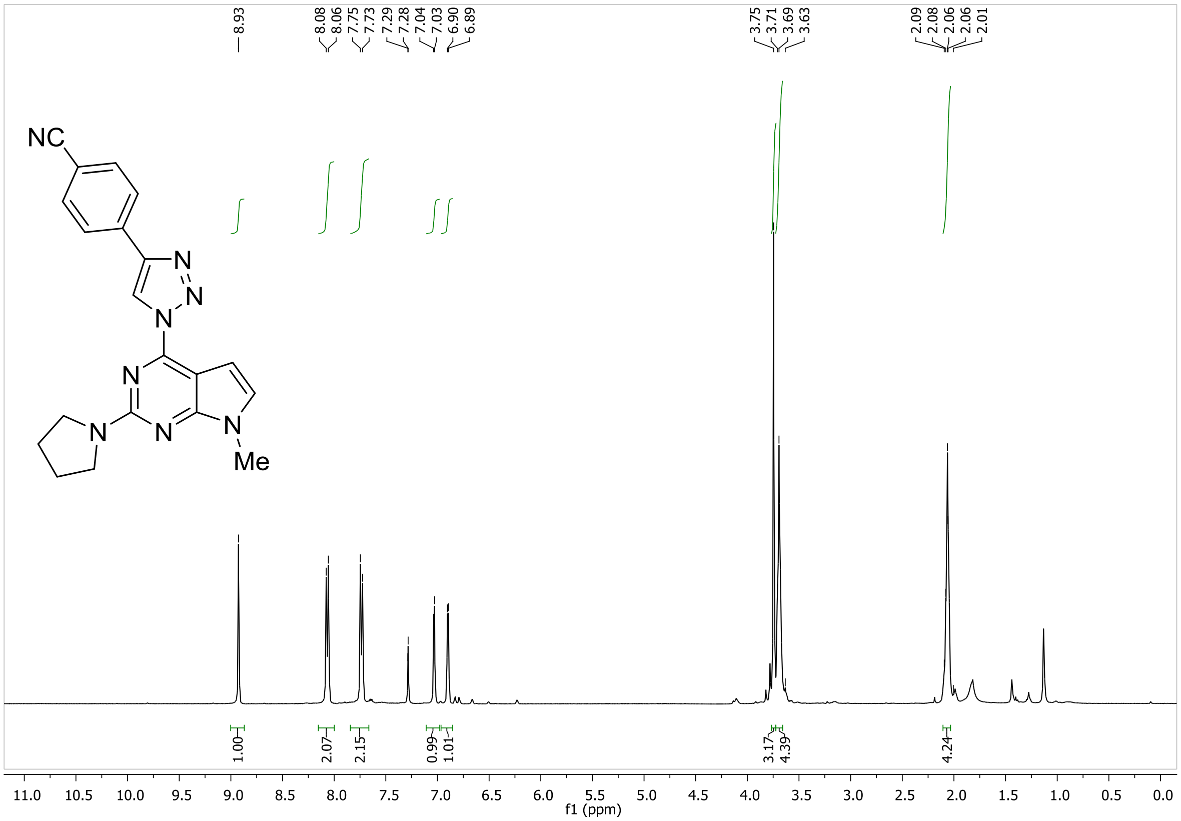

Figure S64: ^{1}H NMR (400 MHz, $CDCl_3$) spectrum.

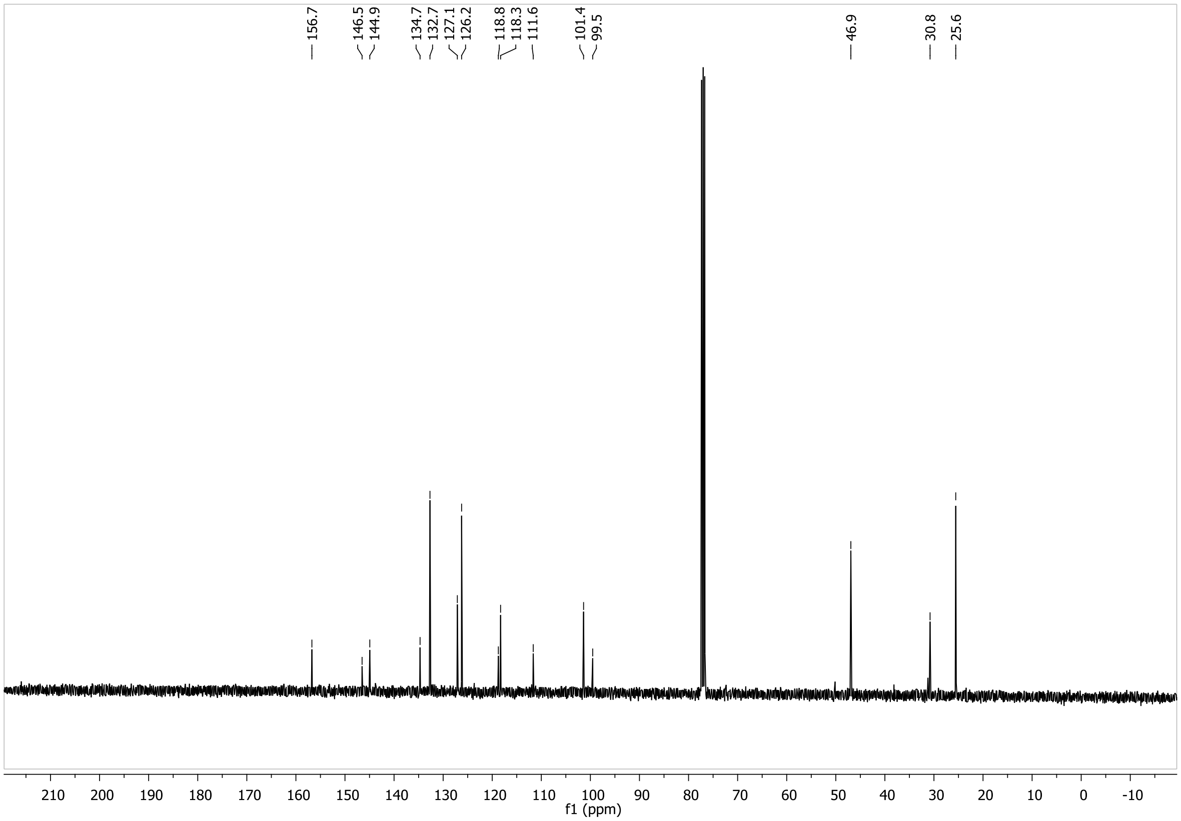

Figure S65: ^{13}C NMR (100 MHz, $CDCl_3$) spectrum.

6-(4-Phenyl-1,2,3-triazol-1-yl)-9-methyl-2-piperidino-7-deazapurine (11a)

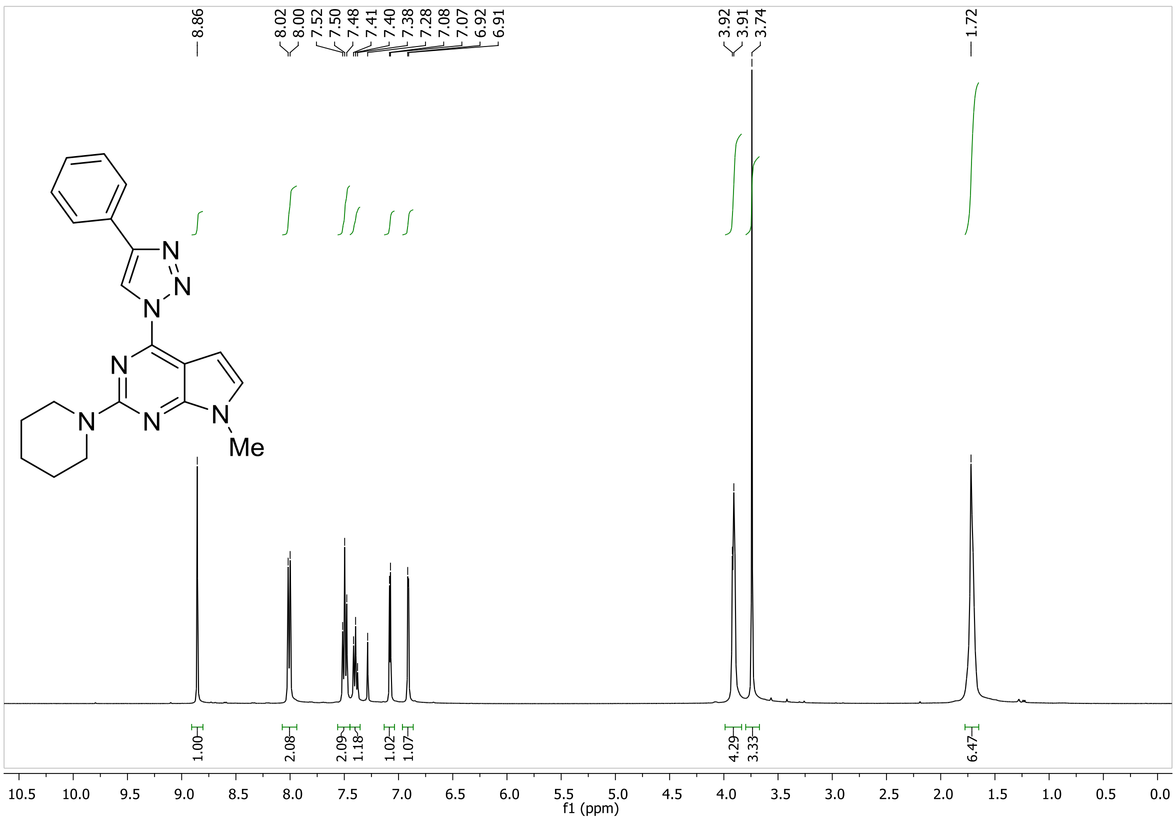

Figure S66: ^{1}H NMR (400 MHz, $CDCl_3$) spectrum.

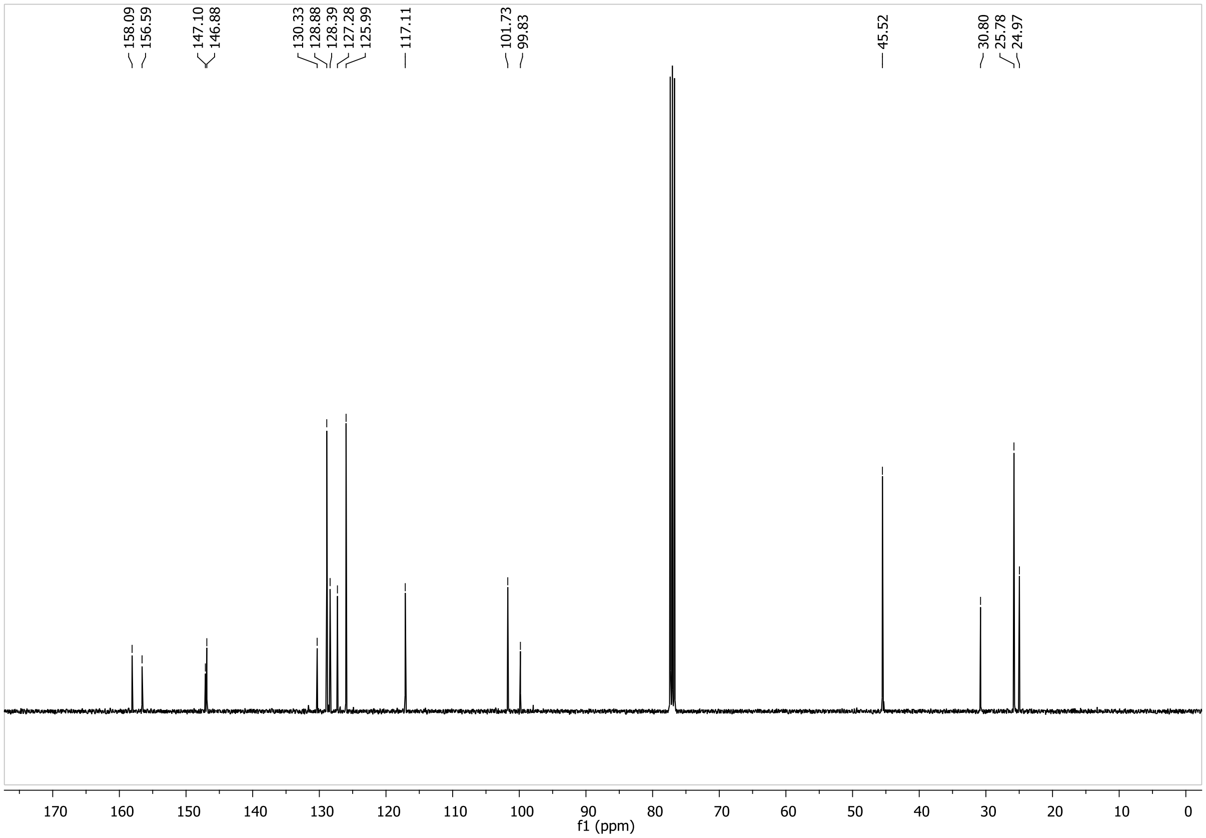

Figure S67: ^{13}C NMR (100 MHz, $CDCl_3$) spectrum.

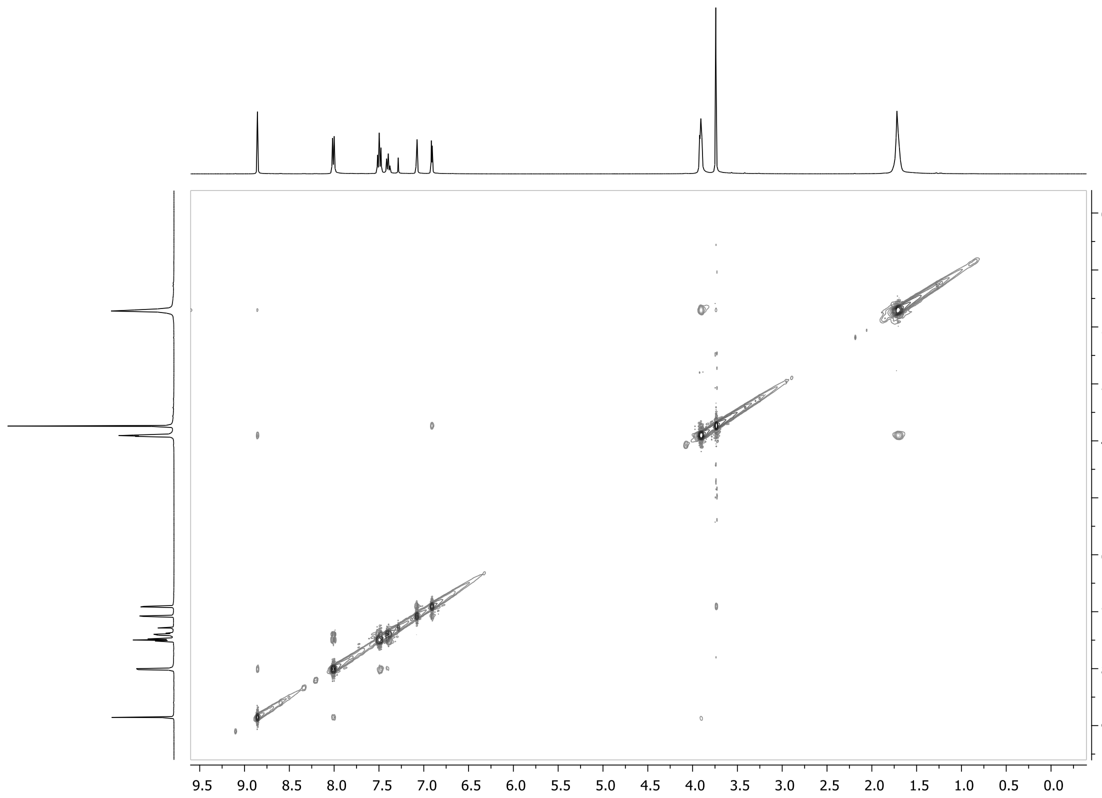

Figure S68: COSY spectrum of compound **11a**.

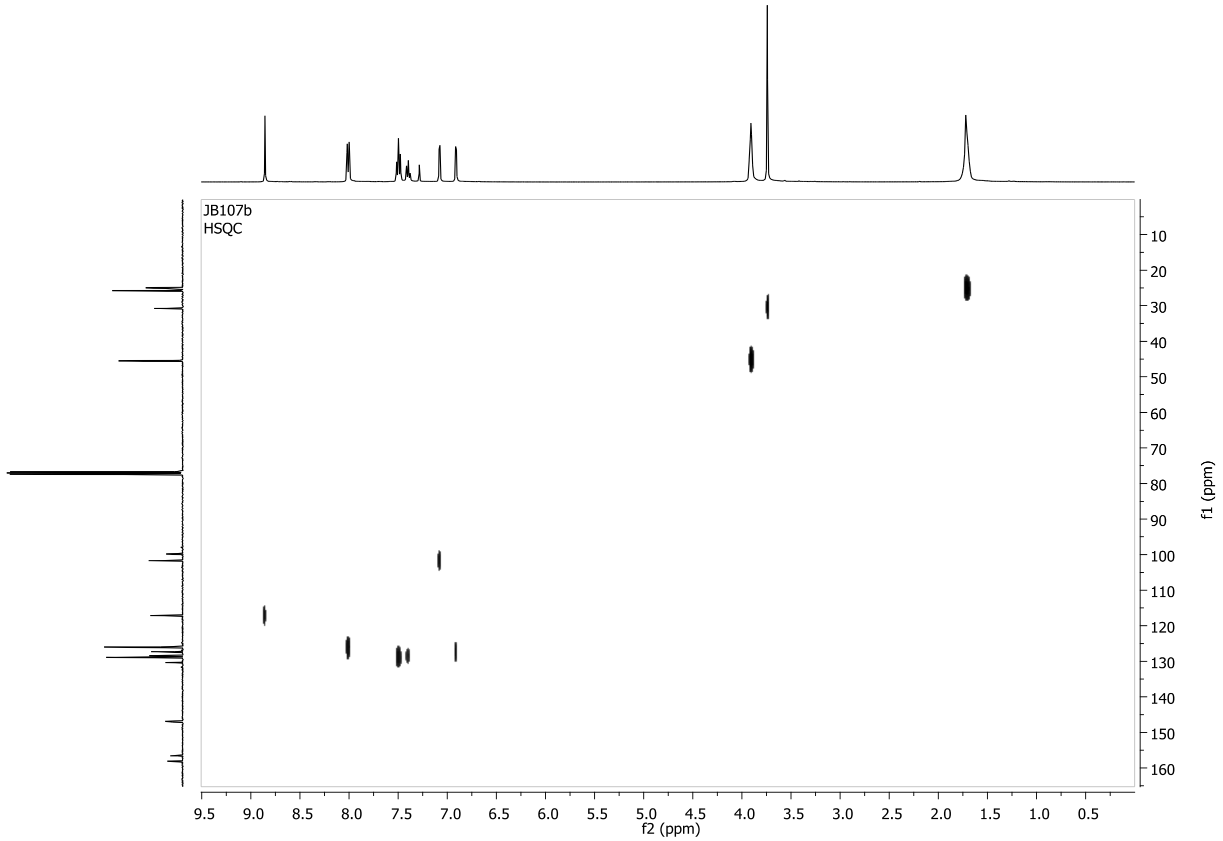

Figure S69: ^{1}H-^{13}C-HSQC spectrum of compound **11a**.

6-[4-(4-Methoxyphenyl)-1,2,3-triazol-1-yl]-9-methyl-2-piperidino-7-deazapurine (11b)

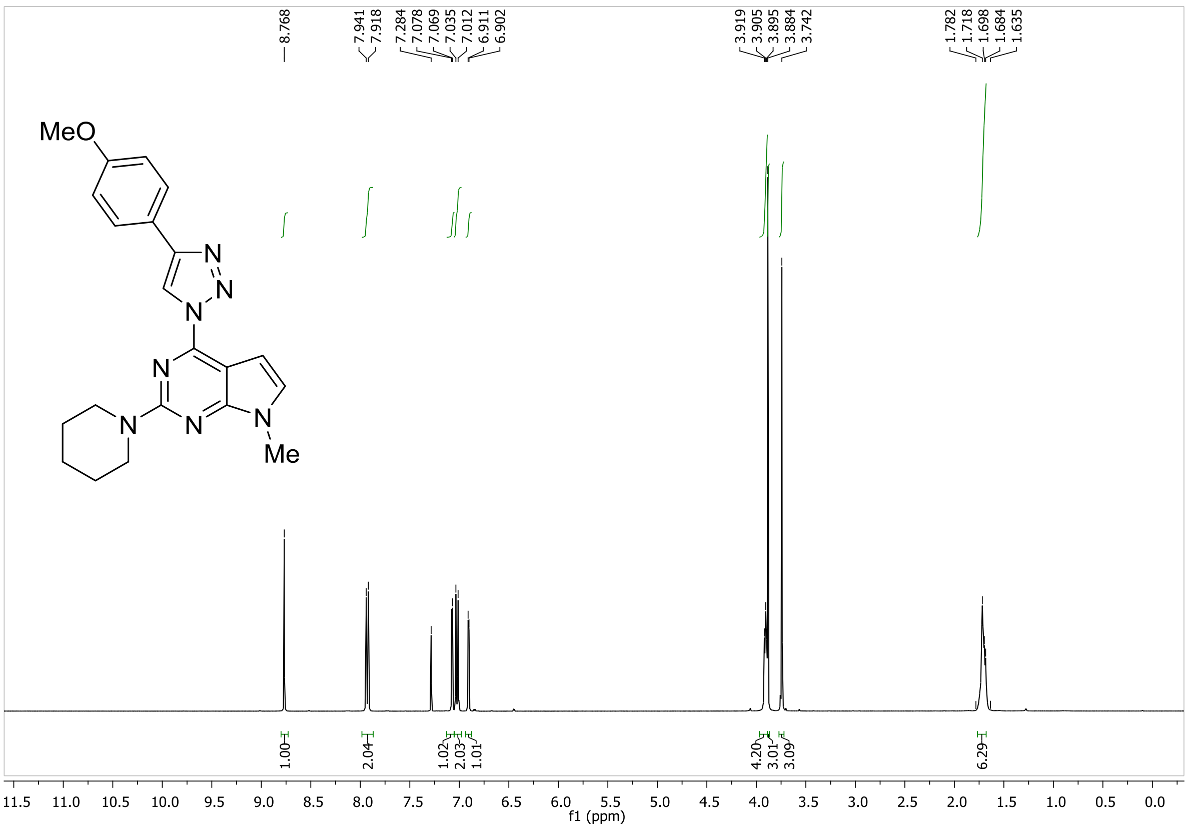

Figure S70: ^{1}H NMR (400 MHz, $CDCl_3$) spectrum.

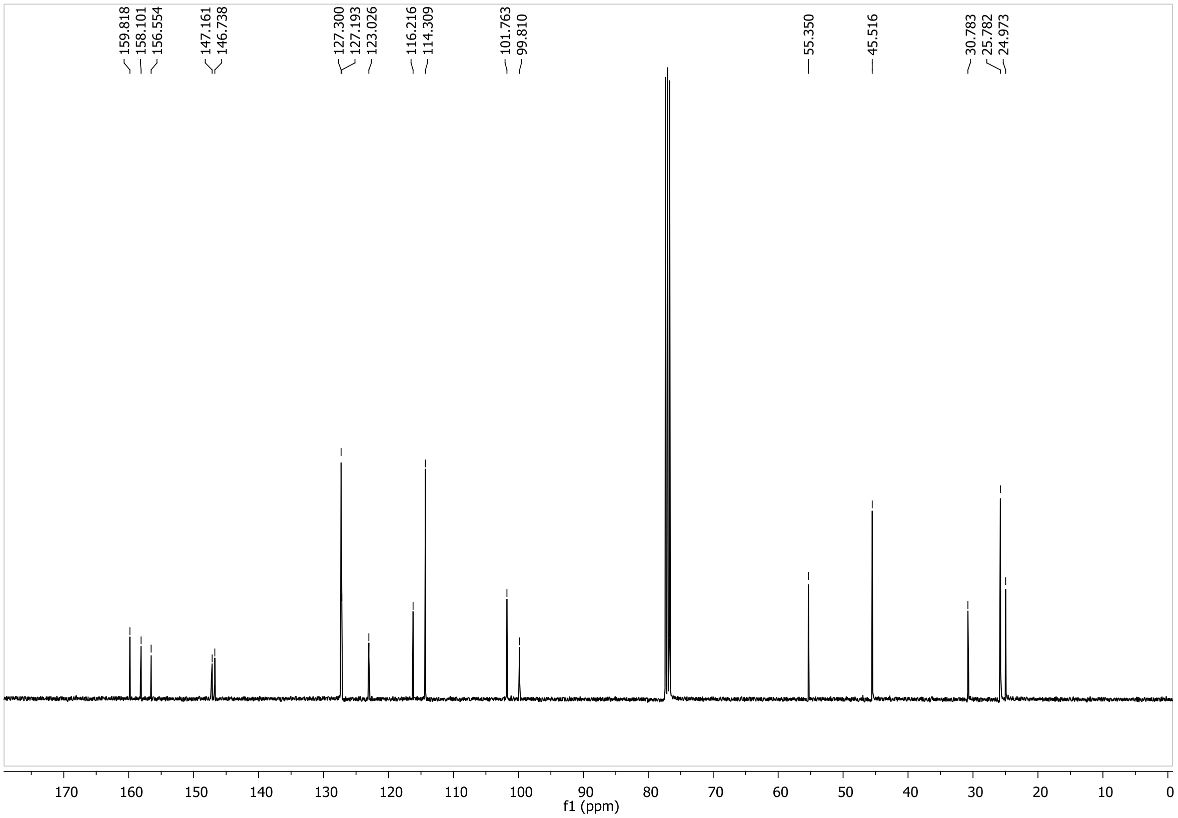

Figure S71: ^{13}C NMR (100 MHz, $CDCl_3$) spectrum.

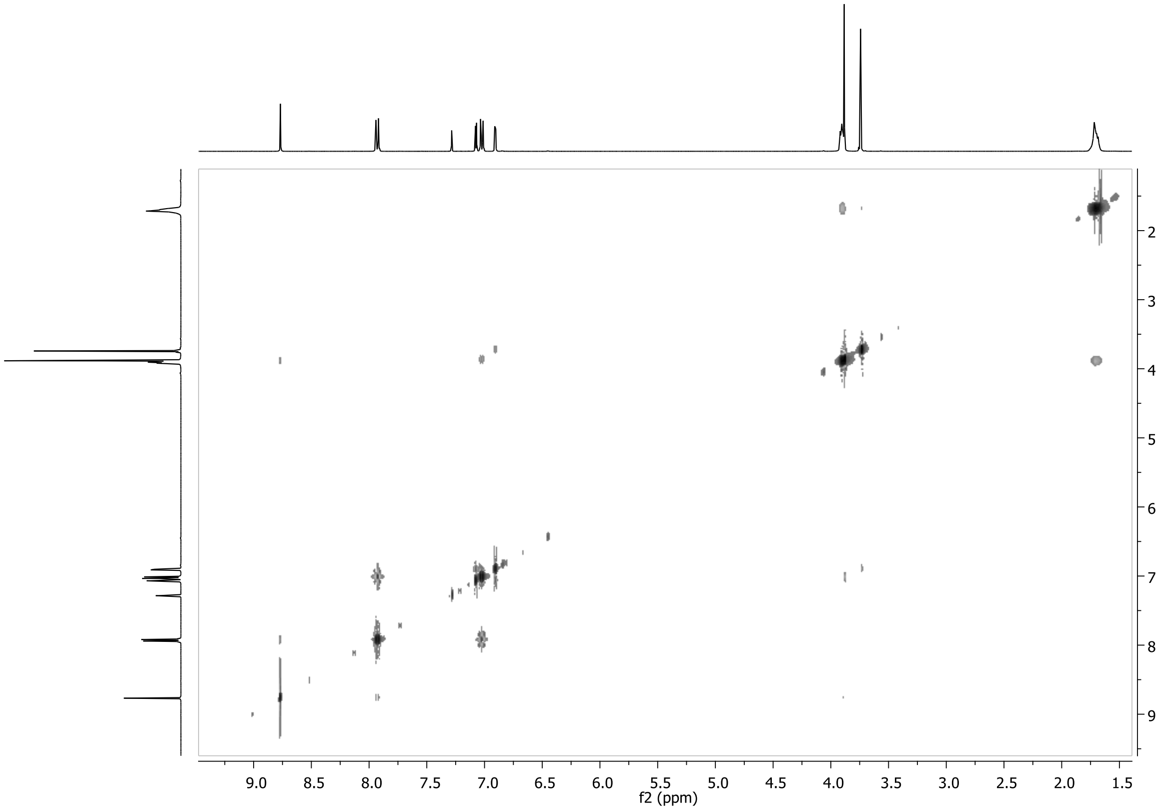

Figure S72: COSY spectrum of compound **11b**.

9-Methyl-6-[4-(4-dimethylaminophenyl)-1,2,3-triazol-1-yl]-2-piperidino-7-deazapurine (11c)

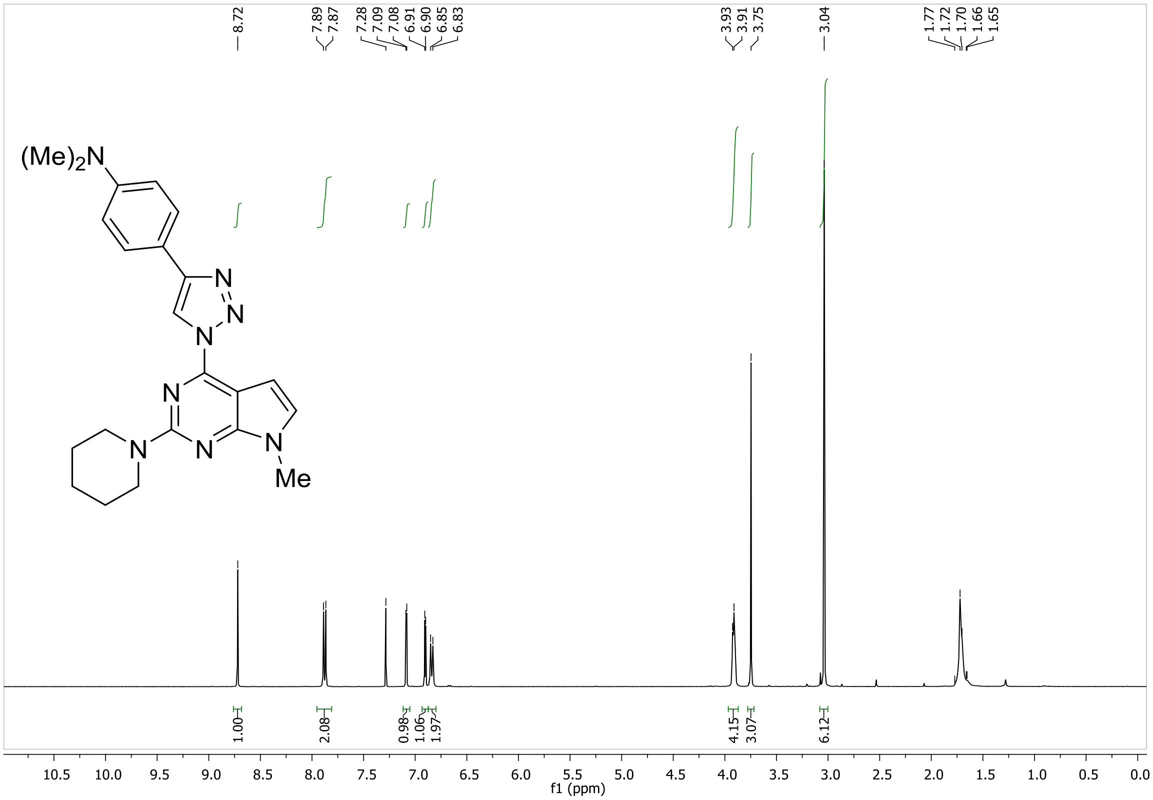

Figure S73: ^{1}H NMR (400 MHz, $CDCl_3$) spectrum.

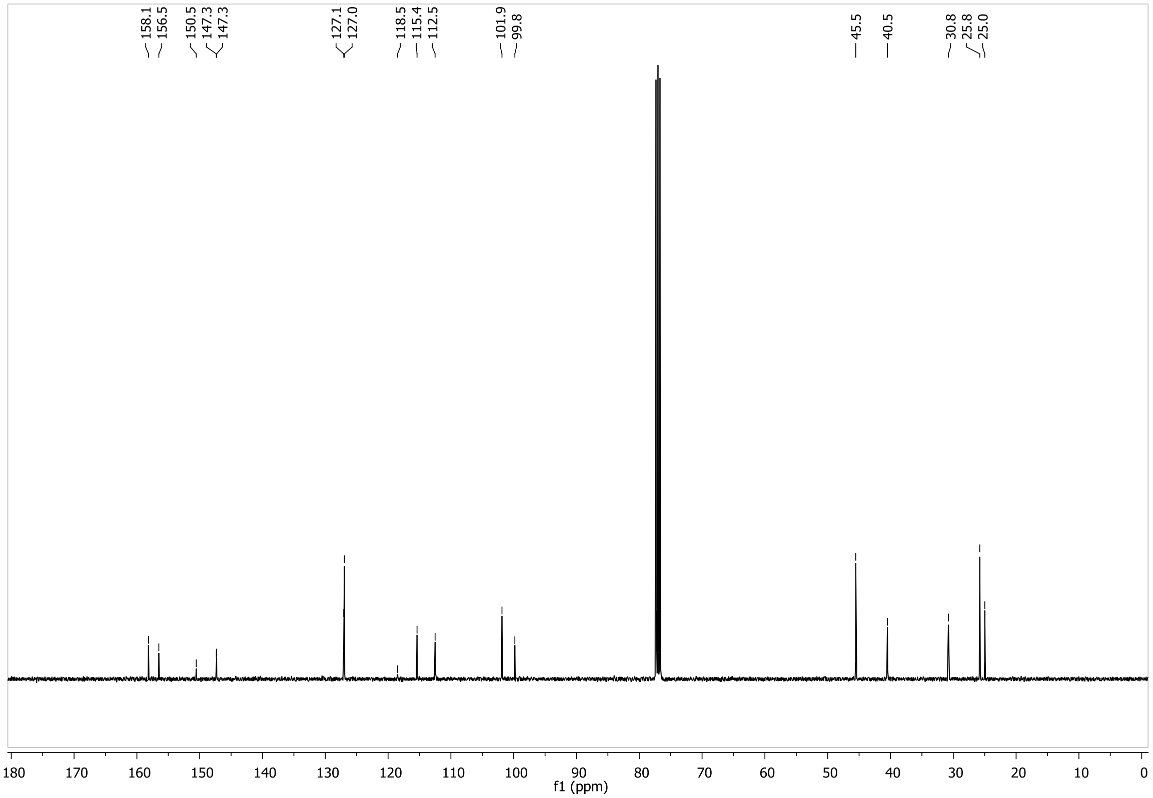

Figure S74: ^{13}C NMR (100 MHz, $CDCl_3$) spectrum.

6-[4-(4-Fluorophenyl)-1,2,3-triazol-1-yl]-9-methyl-2-piperidino-7-deazapurine (11d)

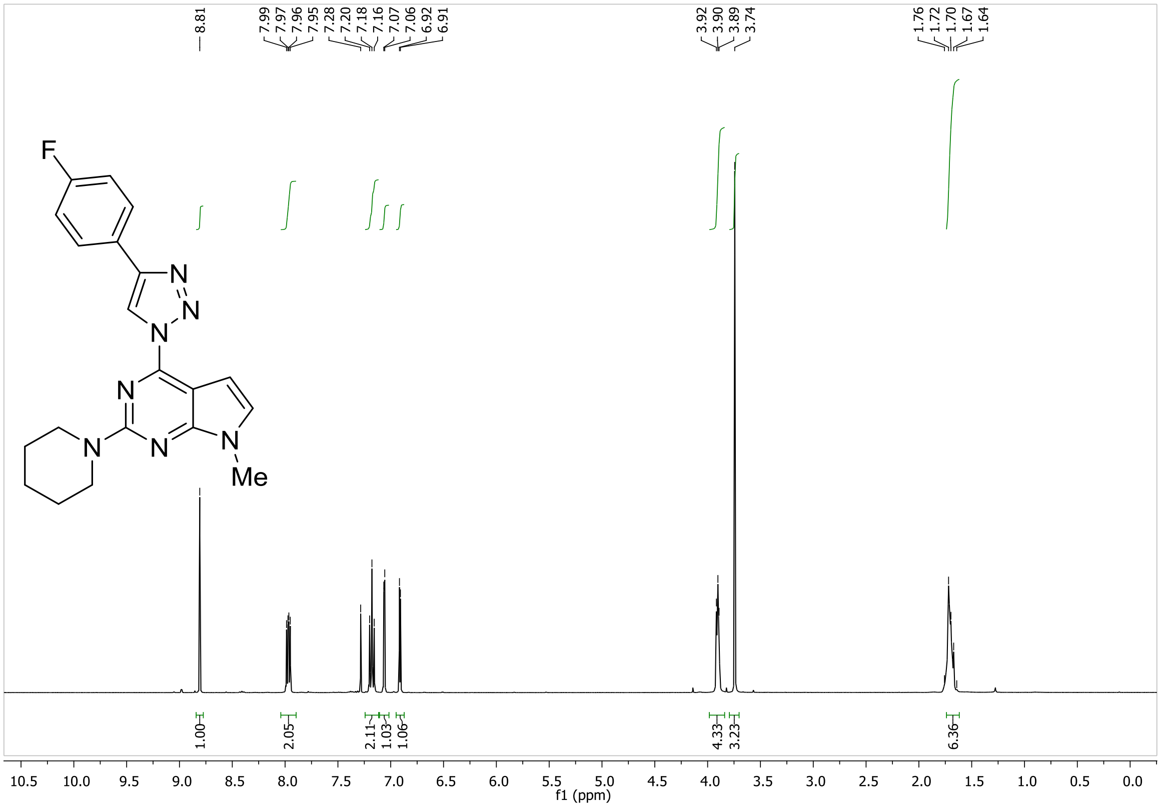

Figure S75: ^{1}H NMR (400 MHz, $CDCl_3$) spectrum.

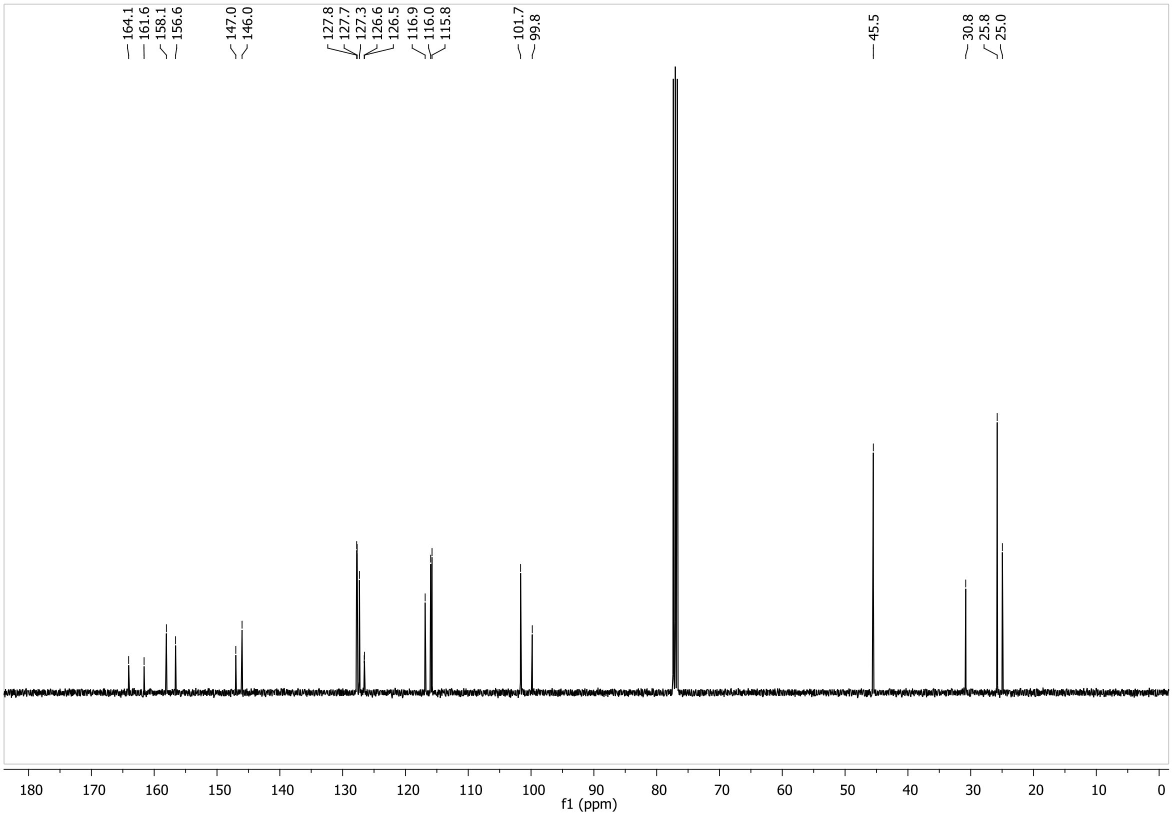

Figure S76: ^{13}C NMR (100 MHz, $CDCl_3$) spectrum.

6-{4-[4-(Trifluoromethyl)phenyl]-1,2,3-triazol-1-yl}-9-methyl-2-piperidino-7-deazapurine (11e)

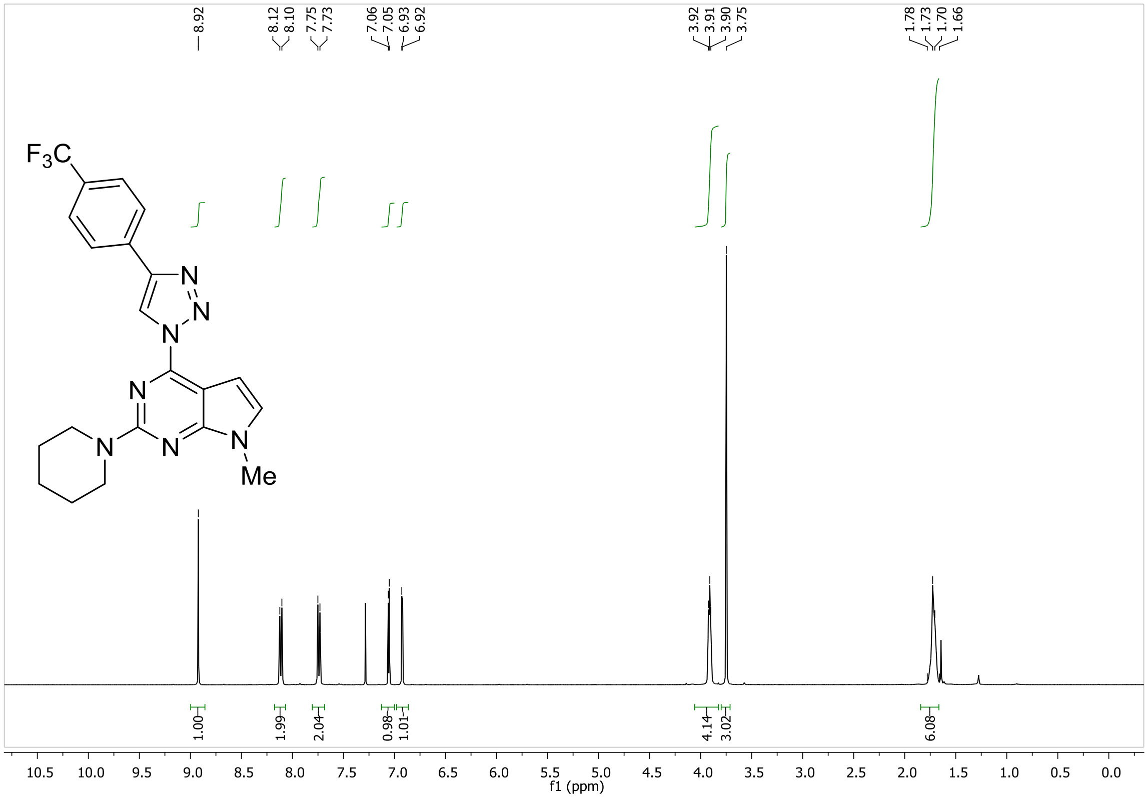

Figure S77: ^{1}H NMR (400 MHz, $CDCl_3$) spectrum.

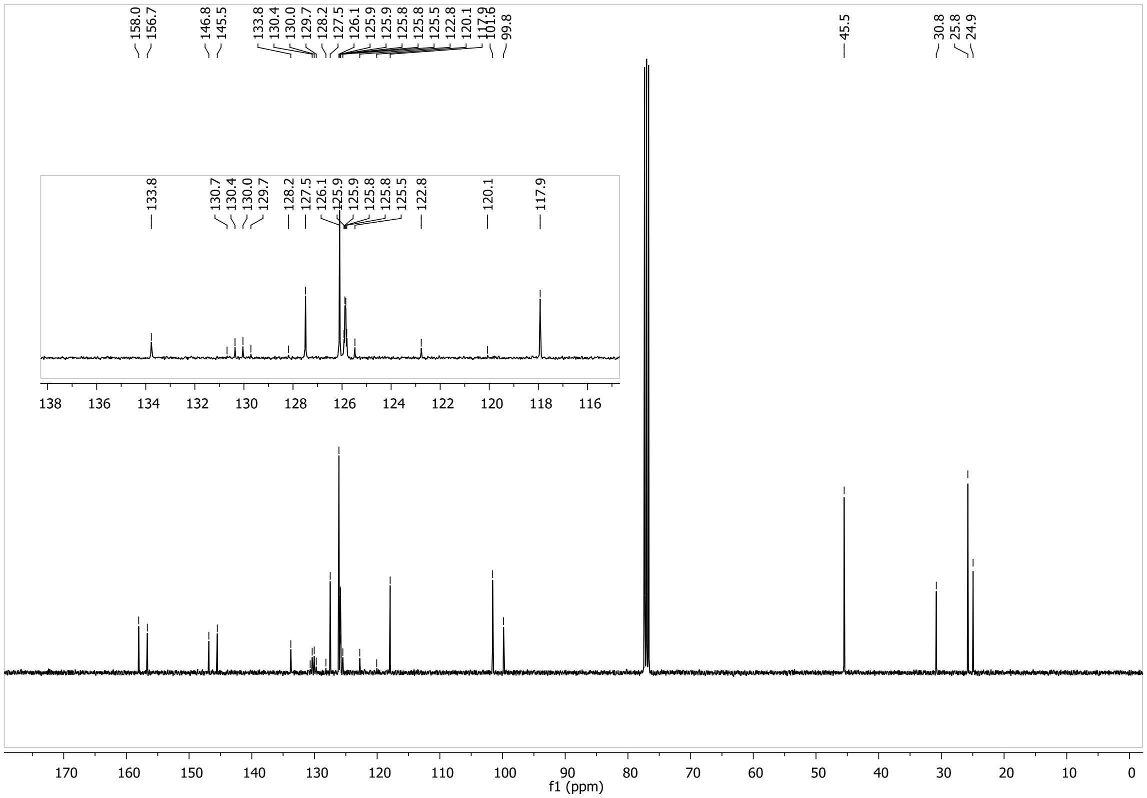

Figure S78: ^{13}C NMR (100 MHz, $CDCl_3$) spectrum.

6-[4-(4-Cyanophenyl)-1,2,3-triazol-1-yl]-9-methyl-2-piperidino-7-deazapurine (11f)

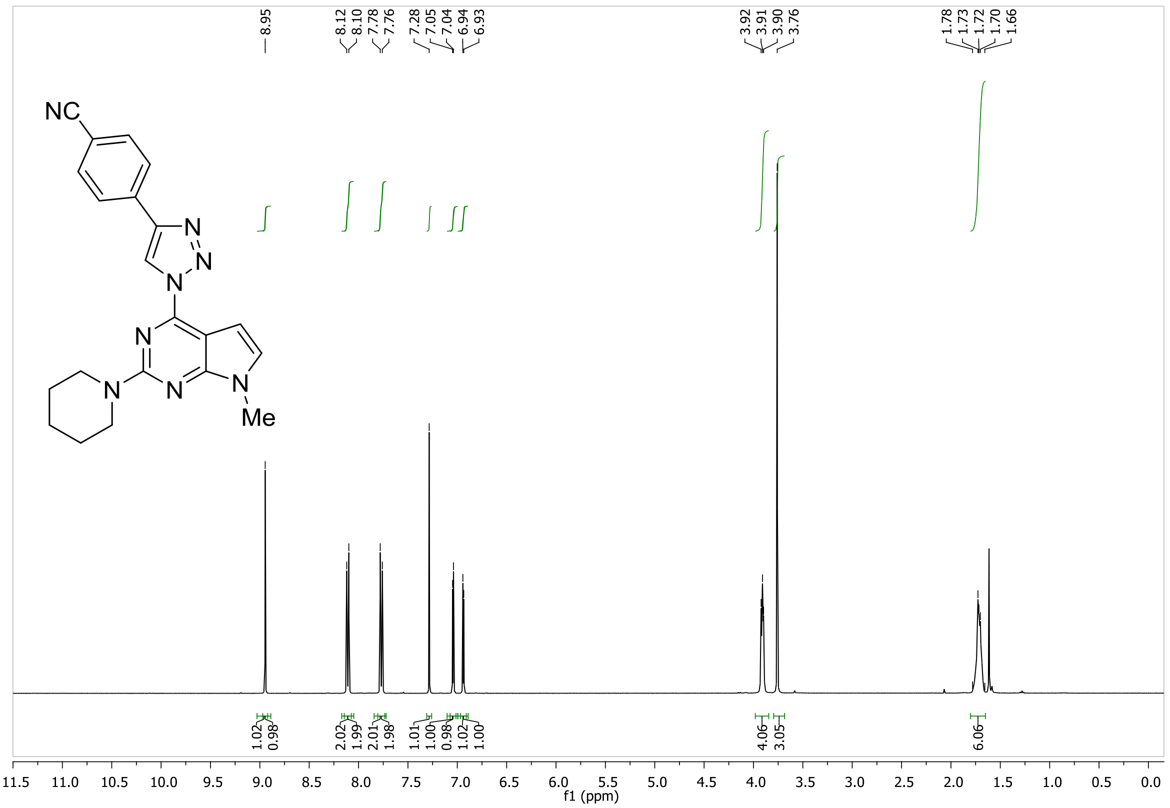

Figure S79: ^{1}H NMR (400 MHz, $CDCl_3$) spectrum.

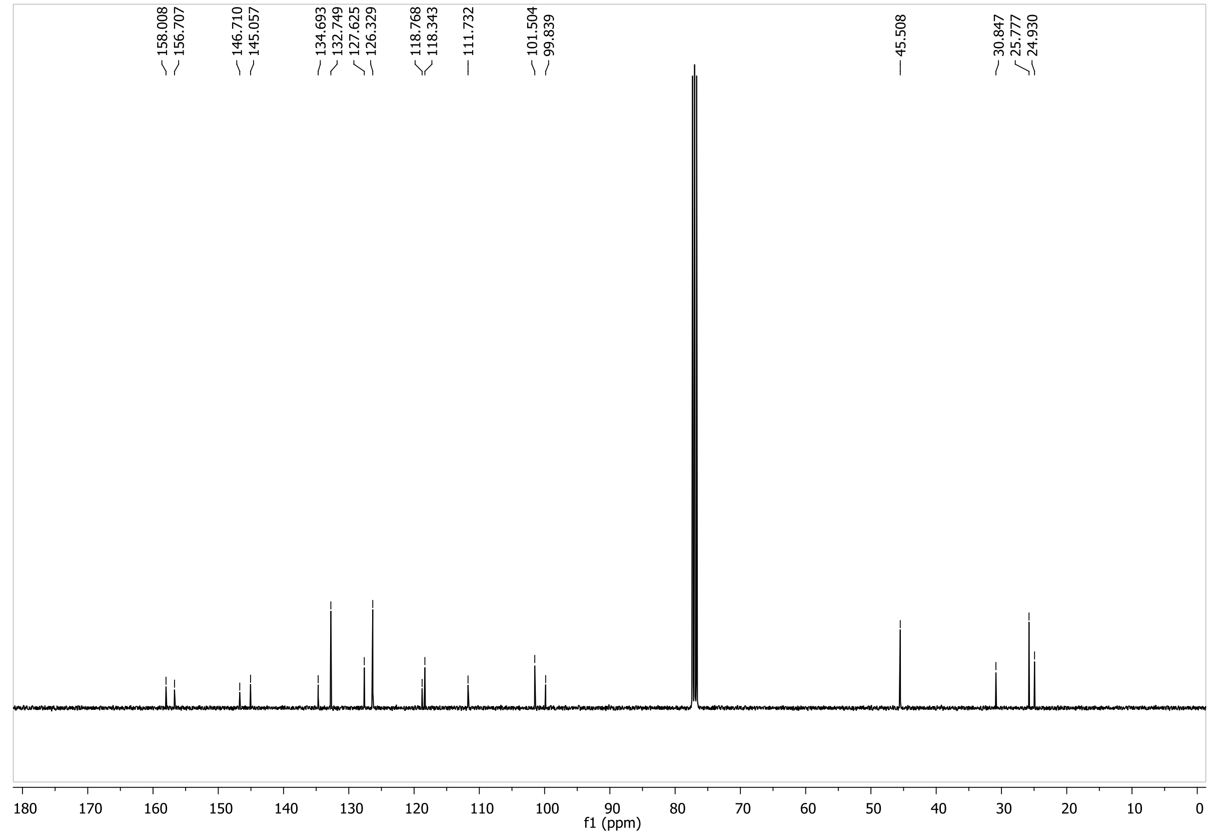

Figure S80: ^{13}C NMR (100 MHz, $CDCl_3$) spectrum.

Toxicity and cell imaging data

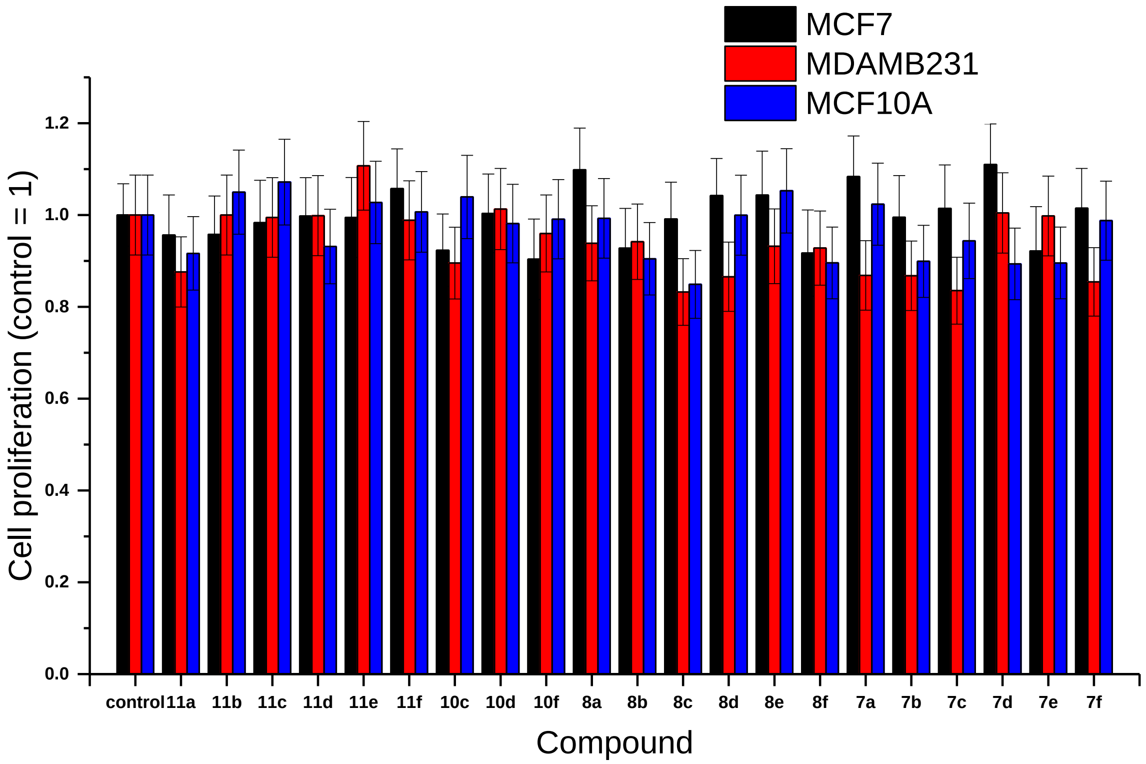

Figure S81: Cytotoxicity effects of the studied compounds to MCF7, MDAMB231 and MCF-10A cell lines.

Compound 11a **Compound 11b**

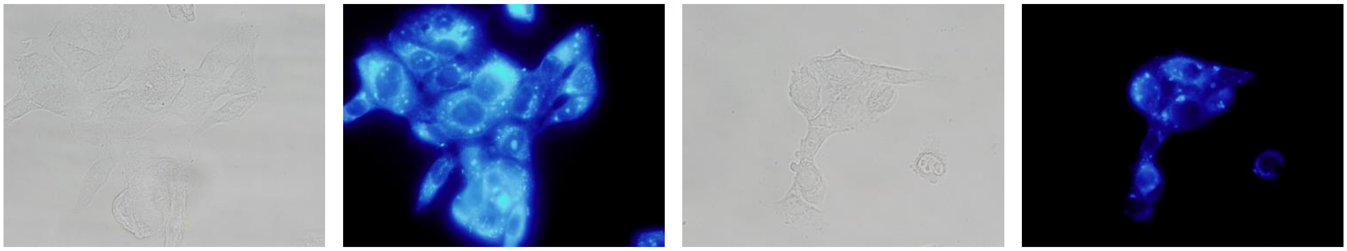

Compound 8c

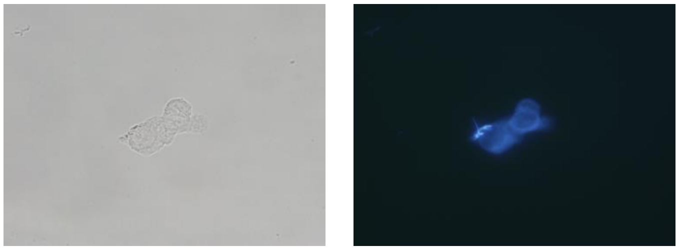

Figure S82: Cell imaging using compounds **11a**, **11b** and **8c** (Material: 100 µM (DMSO 5 %), 1 h, cell: MCF10A Exposure: passed separately through filter (365 ± 20 nm); em >420 nm.

References

[1] Kovaļovs, A.; Novosjolova, I.; Bizdēna, Ē.; Bižāne, I.; Skardziute, L.; Kazlauskas, K.; Jursenas, S.; Turks, M. *Tetrahedron Lett*. **2013**, *54*, 850–853. doi:10.1016/j.tetlet.2012.11.095

[2] Bucevicius, J.; Turks, M.; Tumkevicius, S. *Synlett* **2018**, *29*, 525–529. doi:10.1055/s-0036-159094
